# Electronic and solvent reorganization in proton-coupled electron transfer captured by ultrafast X-rays

**DOI:** 10.1038/s41467-026-75943-4

**Published:** 2026-08-26

**Authors:** Abdullah Kahraman, Michael Sachs, Soumen Ghosh, Benjamin I. Poulter, Estefanía Sucre-Rosales, Elizabeth S. Ryland, Douglas Garratt, Sumana L. Raj, Natalia Powers-Riggs, Subhradip Kundu, Christina Y. Hampton, David J. Hoffman, Giacomo Coslovich, Georgi L. Dakovski, Patrick L. Kramer, Matthieu Chollet, Roberto Alonso-Mori, Tim B. van Driel, Sang-Jun Lee, Kristjan Kunnus, Amy A. Cordones, Robert W. Schoenlein, Eric Vauthey, Amity Andersen, Niranjan Govind, Christopher B. Larsen, Elisa Biasin

**Affiliations:** 1 Physical Sciences Division, Physical and Computational Sciences Directorate, Pacific Northwest National Laboratory, Richland, WA 99354 USA; 2 Stanford PULSE Institute, SLAC National Accelerator Laboratory, Menlo Park, CA 94025 USA; 3 Department of Chemistry, University of Washington, Seattle, WA 98195 USA; 4 Department of Physical Chemistry, University of Geneva, CH-1211 Geneva, Switzerland; 5 Linac Coherent Light Source, SLAC National Accelerator Laboratory, Menlo Park, CA 94025 USA; 6 Stanford Synchrotron Radiation Lightsource, SLAC National Accelerator Laboratory, Menlo Park, CA 94025 USA; 7 Environmental Molecular Sciences Laboratory, Pacific Northwest National Laboratory, Richland, WA 99354 USA; 8 School of Chemical Sciences, University of Auckland, Auckland, 1010 New Zealand; 9 Present Address: Department of Chemistry, Indian Institute of Technology Madras, Chennai, 600036 India

**Keywords:** Photocatalysis, Excited states, Electron transfer

## Abstract

Proton-coupled electron transfer (PCET) is foundational to catalysis, bioenergetics, and energy conversion, yet directly observing the interplay between electronic redistribution, protonation, and solvent reorganization remains challenging. We combine femtosecond optical spectroscopy, ultrafast N K-edge X-ray absorption spectroscopy, and time-resolved X-ray solution scattering to capture the steps of a sequential PCET reaction in water with atomic-site specificity. Using a ruthenium polypyridyl model complex, we resolve the electron redistribution upon photoinduced metal-to-ligand charge transfer and subsequent ( ~ 460 ps) protonation at a ligand nitrogen, as well as the concomitant rearrangement of the first-solvation-shell. Combined with advanced electronic structure and molecular dynamics simulations, our measurements reveal a marked localization of the excited-state electron density at the protonated N site, together with a switch from N···HO to NH···O hydrogen-bonds. These results establish a multimodal X-ray framework for mechanistic insight into PCET and its control in catalysis, artificial photosynthesis, and biological energy flow.

## Abstract (web summary)

Using ultrafast X-ray spectroscopy and scattering, the authors enable atomic-site-specific observations of a sequential proton-coupled electron transfer, revealing electron redistribution and solvent reorganization following photoexcitation and subsequent protonation.

## Introduction

Proton-coupled electron transfer (PCET) lies at the heart of chemical energy conversion, enabling catalytic redox processes, enzyme function, nutrient cycling, and biofuel production by coupling charge transport to proton motion ^1–3^. In solution, particularly in aqueous and biological environments, hydrogen-bond networks and solvation dynamics strongly modulate these reactions, for instance by stabilizing transient intermediates, influencing the thermodynamic favorability and spatial localization of protonation, or even gating proton transfer^4–6^. Despite decades of study, directly observing how electronic redistribution, protonation, and solvent reorganization proceed in real time has remained a fundamental challenge^1,7^. Traditional methods, such as thermodynamic analyses ^8,9^, kinetic isotope effects ^10^, and rate-law studies ^11^, provide indirect evidence that is often insufficient to unambiguously resolve the underlying PCET pathways. Optical and vibrational spectroscopies can infer proton transfer by tracking changes in electronic transitions or characteristic vibrational modes. However, optical spectra generally lack the chemical site specificity needed to identify the specific atomic site of proton transfer, while vibrational spectra are frequently convoluted with bulk-solvent contributions, particularly in hydrogen-bonded environments, making it difficult to disentangle local proton-transfer events from solvent dynamics^12^. As a result, key mechanistic aspects of PCET remain actively debated, including the interplay between electronic and nuclear degrees of freedom and the timing and coupling of electron and proton motions^13,14^. The development of techniques capable of atomic-scale visualization of PCET reactions, considering both electronic and structural dynamics of the solute and the surrounding solvent, would greatly enhance fundamental understanding of PCET mechanisms and help advance the field.

Time-resolved X-ray methods have emerged as powerful tools for overcoming some of these limitations. Core-level spectroscopies provide intrinsic element- and site-specific sensitivity to charge redistribution and local bonding. Recent computational and experimental studies have demonstrated the utility of ultrafast soft X-ray absorption spectroscopy (XAS) for capturing and advancing our understanding of proton transfer dynamics in liquids and small molecules^15–25^. Complementary to X-ray spectroscopy, time-resolved X-ray solution scattering (TR-XSS) has been successfully applied to monitor photoinduced solute structural dynamics and solvent reorganization in real time^26–28^. In particular, TR-XSS has been shown to track solute–solvent hydrogen-bond rearrangements coupled to ultrafast electron transfer^29^. However, to our knowledge, no prior work has combined element-specific X-ray spectroscopy with time-resolved X-ray scattering to simultaneously capture electronic redistribution, excited-state protonation, and coupled solvent reorganization along a PCET reaction coordinate in solution.

**Fig. 1:**
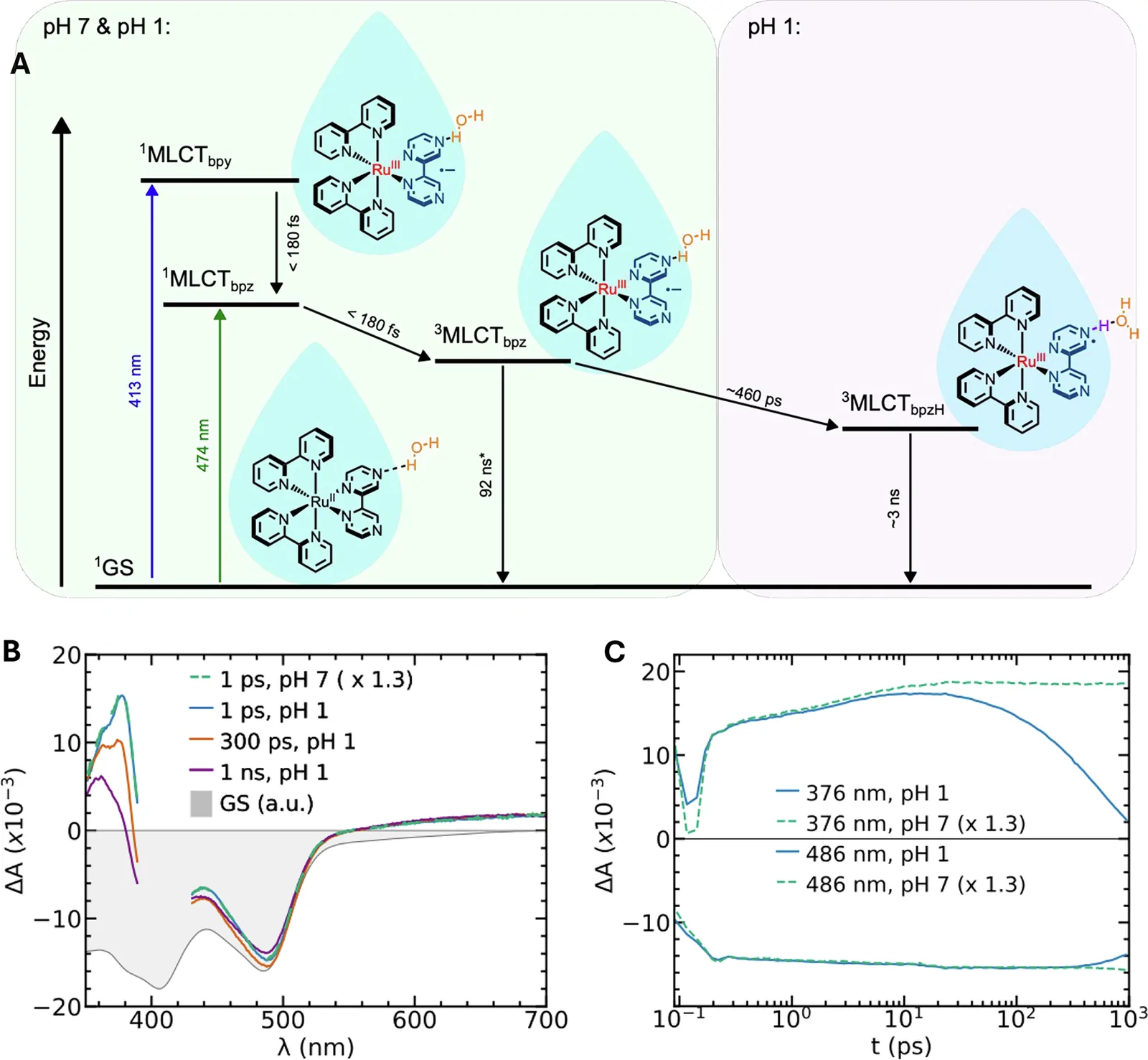
Excited state dynamics of [Ru(bpy)_2_(bpz)]^2+^. **A** Overview of the excited-state dynamics of [Ru(bpy)_2_(bpz)]^2+^ in de-ionized water (pH 7) and 0.1 M HCl solution (pH 1). Time constants are derived from our OTA measurements, except for the 92 ns component, which is taken from ref. ^35^. **B** OTA spectra measured at 1 ps after 400 nm excitation of [Ru(bpy)_2_(bpz)]^2+^ in neutral and acidic conditions. OTA spectra measured at 300 ps and 1 ns after photoexcitation at pH 1, showing spectral evolution due to excited state protonation of the ^3^MLCT_bpz_ state. The gray-shaded area shows the inverted ground-state UV-Vis absorption spectrum. **C** Kinetic traces measured at the excited-state absorption (376 nm) and ground-state bleach (486 nm) show different excited-state dynamics at pH 1 and pH 7.

We herein integrate femtosecond optical spectroscopy, N K-edge ultrafast XAS, and TR-XSS, together with advanced electronic structure and molecular dynamics simulations, to investigate the distinct steps of charge and proton transfer in the model complex [Ru(bpy)_2_(bpz)]^2+^, where bpy is 2,2'-bipyridine, and bpz is 2,2'-bipyrazine (Fig. 1A).

In this model system, the charge and proton transfer steps are sequential and well separated in timescale: the photoinduced electron transfer occurs within our instrument response function, while proton transfer follows on a slower, diffusion-limited timescale in the hundreds of picoseconds regime. Our choice of a system that undergoes sequential PCET, with clear separation of the timescales of electron and proton transfer, makes the spectral signatures associated with each step more interpretable. We further select [Ru(bpy)_2_(bpz)]^2+^ since it undergoes only minimal intramolecular structural changes upon photoexcitation. In combination with the strong X-ray scattering contrast of Ru, this provides a favorable TR-XSS regime: because the intramolecular structural response is weak, the scattering signal is dominated by changes in solute–solvent distances, whose sensitivity is further enhanced by the strong scattering from the Ru center. As a result, this combination simplifies the interpretation of the scattering response and enables a clearer view of solvent reorganization coupled to each step of the reaction.

In this work, we resolve, with element- and site-specific sensitivity, the electronic redistribution following metal-to-ligand charge-transfer excitation and the subsequent excited-state protonation, while simultaneously capturing the solvent reorganization coupled to each step. Together, measurements and simulations reveal how electronic structure, protonation, and solvent reorganization interplay in the intermediate states of [Ru(bpy)_2_(bpz)]^2+^, providing previously inaccessible mechanistic insight into this PCET reaction in solution. This system provides an ideal mechanistic reference point to test our multimodal approach and for extending it to more complex PCET systems, including bimolecular reactions, systems with unknown pathways, and cases in which electronic and/or nuclear degrees of freedom are more strongly coupled.

## Results

### PCET kinetics

[Ru(bpy)_2_(bpz)]^2+^ belongs to a class of transition metal complexes widely used to study light-driven PCET^30–32^, and its optical properties are well-established^33–35^. Its electronic absorption spectrum shows two main MLCT bands: a higher energy transition at 413 nm, where the photoexcited electron localizes on the bpy ligands (MLCT_bpy_), and a lower energy transition at 474 nm, where the electron localizes on the bpz ligand (MLCT_bpz_). Emission occurs exclusively from the lower energy ^3^MLCT_bpz_ state. The ^3^MLCT_bpz_ emission is quenched in the presence of acid due to excited-state protonation, with a reported pKa^*^ of 3.5^33^, whereas the ground-state protonation occurs with a reported pKa of -0.15^34^. Based on these pKa values, pH 1 is ideal for studying photoinduced PCET in [Ru(bpy)_2_(bpz)]^2+^ (Suppl. Fig. 1), offering minimal ground-state protonation, but efficient excited-state protonation, as confirmed by steady-state optical measurements (Suppl. Fig. 2).

Time-resolved emission measurements and single-point transient absorption have been reported for this system^34–36^. Here, we conduct broadband transient absorption to capture the full spectral evolution and establish the excited-state initial conditions for the X-ray experiments. Optical transient-absorption (OTA) measurements in de-ionized water (hereafter pH 7) and 0.1 M HCl aqueous solution (hereafter pH 1) were conducted with an instrument response function (IRF) of  ~ 180 fs. Excitation at 400 nm (into MLCT_bpy_) and 500 nm (into MLCT_bpz_) produces identical OTA spectra and kinetics, demonstrating that inter-ligand electronic redistribution occurs within the IRF under all conditions (Suppl. Fig. 4). Thus, regardless of the excitation wavelength or solution acidity, the system rapidly evolves to the long-lived ^3^MLCT_bpz_ state. Figure 1B shows the OTA spectrum of the ^3^MLCT_bpz_ state measured at 1 ps after excitation. The spectrum exhibits ground-state bleach (GSB) of both MLCT bands, an excited-state absorption (ESA) centered at  ~ 350 nm, and a weak positive transient extending into the near-infrared region. In related complexes, these features have been attributed to the formally reduced polypyridyl ligand and oxidized metal in the ^3^MLCT excited state, respectively^37–39^.

At pH 7, the ^3^MLCT_bpz_ state persists beyond the experimental time window ( ~ 1.4 ns), consistent with the reported 92 ns emission lifetime of [Ru(bpy)_2_(bpz)]^2+^ in water^35^. Kinetic traces (Fig. 1C) show some minor spectral reshaping consistent with vibrational energy relaxation at early timescales, but no other excited-state dynamics.

At pH 1, the early dynamics mirror those at pH 7, followed by spectral evolution over the first few hundred picoseconds (Fig. 1B, C), indicating formation of a new species. The transient absorption kinetics at the  ~ 350 nm ESA band exhibit a clear change in temporal behavior on a hundreds-of-picoseconds timescale, transitioning from an initial rise to a subsequent decay (Fig. 1C). Global analysis^40^ of the pH 1 OTA data (Suppl. Fig. 3) indicates that this new species forms with a time constant of  ~ 460 ps and decays with an accelerated lifetime of  ~ 3.3 ns. The latter is consistent with a previous report on the decay of the non-emissive excited-state of [Ru(bpy)_2_(bpz)]^2+^ in acidic solution^36^. The OTA spectra at 300 ps and 1 ns in Fig. 1B show that this new species is spectrally similar to the ^3^MLCT_bpz_ state, but the ESA at  ~ 350 nm blue-shifts and decreases in amplitude. As this ESA is attributed to the reduced bpz, its spectral change is thus consistent with a change in electronic structure of the bpz ligand upon protonation, but provides no further detail on the electronic redistribution. Our time-dependent density functional theory (TDDFT) calculations reproduce the OTA observables (Suppl. Fig. 6). However, valence TDDFT spectra are difficult to interpret because of the high degree of delocalization and mixed character of the excitations. To directly capture electronic redistribution at the ligand level, N K-edge XAS is necessary.

In summary, our OTA measurements reveal rapid population of a long-lived ^3^MLCT_bpz_ state. At pH 7 this state is long-lived (> 1.4 ns), while at pH 1, it is protonated with an observed time-constant of 460 ps. Additional OTA measurements as a function of pH and isotopic substitution (Suppl. Fig. 5) support a diffusion-limited mechanism for the formation of the protonated excited state. In the following X-ray studies, we will probe the ^3^MLCT_bpz_ state at 1 ps, and the protonated MLCT state (^3^MLCT_bpzH_) at 100 ps, 300 ps, and 1 ns, with the protonated population reaching its maximum at 1 ns (73%), and the 100 ps and 300 ps delays probing  ~ 20 % and  ~ 60% of this maximum population, respectively.

### Site-specific electronic signatures of PCET

**Fig. 2:**
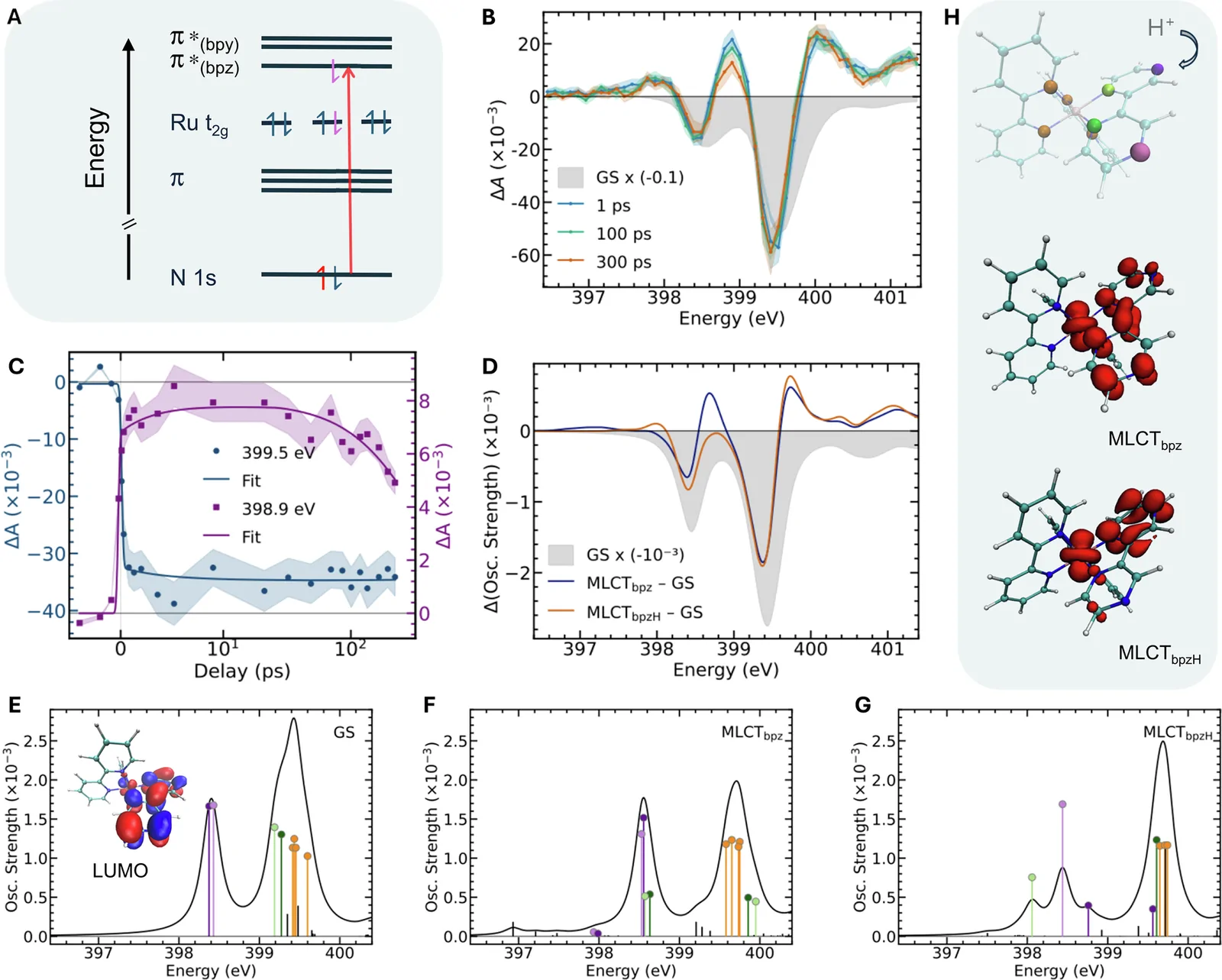
Ultrafast N K-edge response of [Ru(bpy)_2_(bpz)]^2+^ and assignment with TDDFT. **A** Schematic molecular orbital diagram depicting the MLCT process (pink electrons) and a representative N K-edge XAS transition (red electron/arrow). **B** Transient N K-edge spectra recorded at 1, 100, and 300 ps after 400 nm excitation at pH 1, reported with 95% confidence interval calculated from the standard deviation across 16 independent runs (1 and 300 ps) and 8 independent runs (100 ps). The gray-shaded area represents the negatively scaled ground-state absorption. **C** Kinetic traces at 399.5 eV (blue, left axis) and 398.9 eV (magenta, right axis) with 95% confidence intervals calculated from the standard deviation across 13 runs (398.9 eV) and 5 runs (399.5 eV). Solid lines show the fits obtained from a sequential kinetic model, in which the time constants are fixed to the values obtained from the global analysis of the OTA data. For the GSB, the model includes a 4 ps component (vibrational relaxation) and a 3 ns component (decay of the excited-state). For the positive transient, an additional 460 ps component is included to account for excited-state protonation. **D** TDDFT calculations of the ^1^GS N K-edge spectra (gray fill, shown inverted) and transient spectra corresponding to the ^3^MLCT_bpz_ (blue) and ^3^MLCT_bpzH_ (orange) states. **E**–**G** Calculated N K-edge spectra (black, 0.25 eV Lorentzian-broadened) with underlying transitions for ^1^GS (**E**),  ^3^MLCT_bpz_ (**F**), and  ^3^MLCT_bpzH_ (**G**) states. The stick colors identify the nitrogen atom involved in the transition, using the same color scheme as in panel H (top): bpy-N (orange), coordinated bpz-N (green), peripheral bpz-N (purple), and the protonated peripheral bpz-N (dark purple). The inset of panel E shows isosurfaces of the ground-state lowest unoccupied molecular orbitals (with isovalue of  ± 0.03 a.u.). **H** Top: molecular structure indicating the color-coded individual Ns: bpy-N (orange), coordinated bpz-N (green), peripheral bpz-N (purple; dark purple marks the protonated N). Middle/Bottom: spin-density isosurfaces of the corresponding ^3^MLCT_bpz_/^3^MLCT_bpzH_ states, plotted at an isovalue of  ± 0.005 a.u.

The N K-edge XAS spectrum arises from N 1s core-level excitations to unoccupied π* orbitals with N 2p character (Fig. 2A). The steady-state N K-edge spectrum of [Ru(bpy)_2_(bpz)]^2+^, plotted as inverted gray shading in Fig. 2B, features two peaks that clearly differentiate peripheral from Ru-coordinated N environments. The low-energy band at 398.5 eV is assigned to 1s  → π* transitions of the peripheral bpz Ns, while the more intense, higher energy band at 399.5 eV is assigned to transitions involving the six Ns directly coordinated to Ru^41–43^. Transitions at higher energy correspond to excitations into higher-lying unoccupied molecular orbitals and are not considered further in this work.

Figure 2 B also shows transient N K-edge spectra collected upon photoexcitation of [Ru(bpy)_2_(bpz)]^2+^ at pH 1. The transient at 1 ps (blue) site-selectively probes the ^3^MLCT_bpz_ state and exhibits GSBs at 398.4 and 399.5 eV, with positive transients at 398.9 and 400.0 eV. As supported by further analysis (Suppl. Fig. 9), these features arise from a blue-shift and reduced intensity of the high-energy band, as well as a slight blue-shift of the low-energy band. A weaker positive transient at 397.9 eV is also observed.

Transient spectra at 100 ps (green) and 300 ps (orange) show a subsequent decrease in the positive transient at 398.9 eV, corresponding to the blue-shifted low-energy band assigned to transitions of peripheral bpz Ns. We attribute this decrease in intensity to protonation of a peripheral bpz N. Kinetic data (Fig. 2C) are consistent with the reshaping of the 300 ps signal with respect to the 1 ps signal, indicating formation of a new species. The GSB measured at 399.5 eV (assigned to transitions of the Ru-coordinated Ns insensitive to peripheral protonation) appears instantaneously upon photoexcitation and remains constant. In contrast, the positive signal at 398.9 eV decays over time. These dynamics are consistent with the kinetic model derived from the OTA data (solid lines in Fig. 2C). We note that, due to the higher-concentration regime of the XAS experiment, we evaluated the impact of the full second-order kinetics on the protonation timescale, which results in only a modest ( ~ 15 %) deviation from the pseudo-first-order description. This difference remains within the uncertainty of the XAS kinetics, and the data are therefore adequately described using the same effective time constant. Additional measurement at pH 7, where excited-state protonation is not expected, shows no spectral reshaping in the 1-300 ps timescales (Suppl. Fig. 7).

To interpret the N K-edge XAS data, we computed N 1s X-ray absorption spectra for the ground-state (^1^GS), the lowest triplet MLCT state (^3^MLCT_bpz_), and the protonated triplet MLCT state (^3^MLCT_bpzH_) using TDDFT. As detailed in the Methods, each spectrum was averaged over 20 QM/MM snapshots to explicitly account for solute-solvent interactions. The simulated ground-state and transient spectra (Fig. 2D) reproduce the key experimental features, confirming the assignment of the 1-300 ps changes in our XAS data to formation of the protonated excited-state.

Given the agreement between experiment and calculations, the latter can be used to describe the electronic redistribution in both ^3^MLCT_bpz_ and ^3^MLCT_bpzH_ states and rationalize the observed spectral changes. In contrast to valence excitations probed by OTA, core-level spectra primarily probe a few unoccupied molecular orbitals, enabling orbital-level interpretation^44^. Figure 2E–G display the calculated transitions for a representative snapshot of each electronic state. Transitions are color-coded by N type (green for coordinated bpz Ns, purple for peripheral bpz Ns, and orange for bpy Ns) as shown in the top panel of Fig. 2H. The following description of the most intense transitions in each spectrum is based on the detailed analysis provided in Suppl. Note 5.

In the ^1^GS (Fig. 2E), the calculations confirm that the low-energy band arises mainly from N 1s  → LUMO transitions of peripheral bpz Ns, with the LUMO localized on the bpz ligand (shown in the inset). The higher energy band comprises transitions from the 1s orbitals of (i) the coordinated bpz Ns to the LUMO and (ii) bpy Ns to the bpy-localized π* orbitals. Because coordination to Ru stabilizes the N 1s levels, transitions from Ru-coordinated bpz N atoms into the LUMO appear at higher energy than those from peripheral bpz N atoms.

In the ^3^MLCT_bpz/bpzH_ states, an electron is promoted from the Ru-localized HOMO to the LUMO. As shown by the spin-density plots in Fig. 2H, in the ^3^MLCT_bpz_ the electron delocalizes over the bpz ligand, while in the ^3^MLCT_bpzH_ state electron density localizes on the pyrazine ring containing the protonated N. The orbitals remain generally localized on either bpy or bpz, and transitions from bpy N 1s orbitals occur predominantly into bpy-localized π* orbitals, whereas transitions from bpz N 1s orbitals occur predominantly into bpz-localized π* orbitals.

We first examine the excited-state changes of the high-energy XAS peak, which is dominated by the bpy Ns transitions, color-coded in orange. In the ^3^MLCT_bpz_ state (Fig. 2F), the bpy N 1s orbitals are stabilized by approximately 0.7 eV due to loss of electron density because of enhanced *σ*-donation to the formally Ru^III^. The bpy π* orbitals are also stabilized, though less strongly. Accounting for Coulomb and exchange interactions, the net effect is a predicted  ~ 0.2 eV blue-shift of the high-energy XAS peak, consistent with our experiment, as well as with prior measurements on photoexcited [Ru(bpy)_3_]^2+^^41^. In the ^3^MLCT_bpzH_ state (Fig. 2G), the increased positive charge of the complex induces an overall stabilization of all molecular orbitals. Unsurprisingly, due to the distal proximity of the bpy ligands to the protonation site, there is no net change on the bpy N 1s  →  π* transitions between the protonated and unprotonated ^3^MLCT states. Thus, the higher energy XAS peak is mostly unaffected by protonation, consistent with our measurements.

We next examine the evolution of the low-energy XAS feature arising from bpz N 1s transitions. In the ^3^MLCT_bpz_ state (Fig. 2F), increased electron density on the bpz ligand destabilizes the N 1s levels of both Ru-coordinated and peripheral Ns by approximately 0.4 eV. For the coordinated Ns (green), this core-level destabilization primarily determines the red-shift of their transitions. For the peripheral Ns (purple), however, their dominant transitions target π* orbitals higher in energy than the lowest unoccupied bpz-localized π* orbitals. This offsets the N 1s destabilization, yielding a small net blue-shift for these transitions. Although difficult to visualize directly from the spin-density plots, the Löwdin population analysis indicates slightly higher electron density on the coordinated Ns, which likely stabilizes their π* orbitals relative to those of the peripheral Ns. Overall, TDDFT predicts a  ~ 0.1 eV blue-shift of the low-energy XAS peak, in agreement with experiment.

In the ^3^MLCT_bpzH_ state (Fig. 2G), electron density localizes on the pyrazine ring containing the protonated N. Consequently, N atoms on the unprotonated pyrazine ring (dark green and light purple) exhibit transitions similar in energy and intensity to those in the ground state. The protonated N (dark purple) shows blue-shifted transitions due to 1s orbital stabilization by the added positive charge, along with reduced oscillator strengths relative to the ^1^GS and ^3^MLCT_bpz_ states due to the accumulation of the electron density at its site. Although the transition of the protonated N blue-shifts, the simultaneous reduction in oscillator strength renders it too weak to generate a positive transient above the ground-state baseline on the high-energy shoulder of the peak. The net observable effect is therefore a reduction in the overall intensity of the low-energy XAS peak, without an accompanying blue shift. This contrasts with the ^3^MLCT_bpz_ state, where the low-energy XAS peak blue-shifts as a whole and the oscillator strengths remain significant, producing a positive transient on the high-energy shoulder. These trends are consistent with the experimentally and theoretically observed positive transient at 398.9 eV in the ^3^MLCT_bpz_ state and its subsequent decrease upon protonation (Fig. 2B and C).

An additional calculated low-energy transition involving the coordinated bpz N of the protonated ring (light green) is consistent with the positive transient at 397.9 eV. The calculations predict transitions in this energy range also for the ^3^MLCT_bpz_ state, but with intensities too weak to yield an observable transient. A closer comparison of the measured signals at 1 ps (^3^MLCT_bpz_) and 300 ps (^3^MLCT_bpzH_) reveals a reshaping of the 397.9 eV feature, supporting its assignment to distinct electronic origins in the two states. Finally, weak transitions predicted below 398.4 eV correspond to N 1s  → Ru 4d (*π*) excitations that borrow dipole intensity via Ru d-N p orbital mixing^41^, but these are not observed experimentally.

In summary, our combined measurements and TDDFT calculations show that the ground-state N K-edge XAS spectrum of [Ru(bpy)_2_(bpz)]^2+^ clearly distinguishes 1s  →  π* transitions of Ru-coordinated and peripheral bpz Ns. In the ^3^MLCT_bpz_ state, the spectral changes reflect decreased electron density on the bpy Ns, and increased density on the bpz Ns, with slightly higher electron density on the coordinated Ns. In the ^3^MLCT_bpzH_ state, we measure a reduced oscillator strength for the 1s  →  π* transition of the protonated N, indicating electron density localization on the protonated pyrazine ring. Kinetic traces are well described by the same model used for OTA, demonstrating consistency between the two techniques and yielding a unified picture of the excited-state dynamics. Although protonation of the peripheral N is chemically intuitive, the element- and site-specific nature of N K-edge XAS enables direct assignment of the observed spectral changes to the protonated nitrogen site, and reveals how protonation reshapes the electronic distribution of the excited states — a feature that has not been previously experimentally resolved.

### Solvent reorganization coupled to PCET

**Fig. 3:**
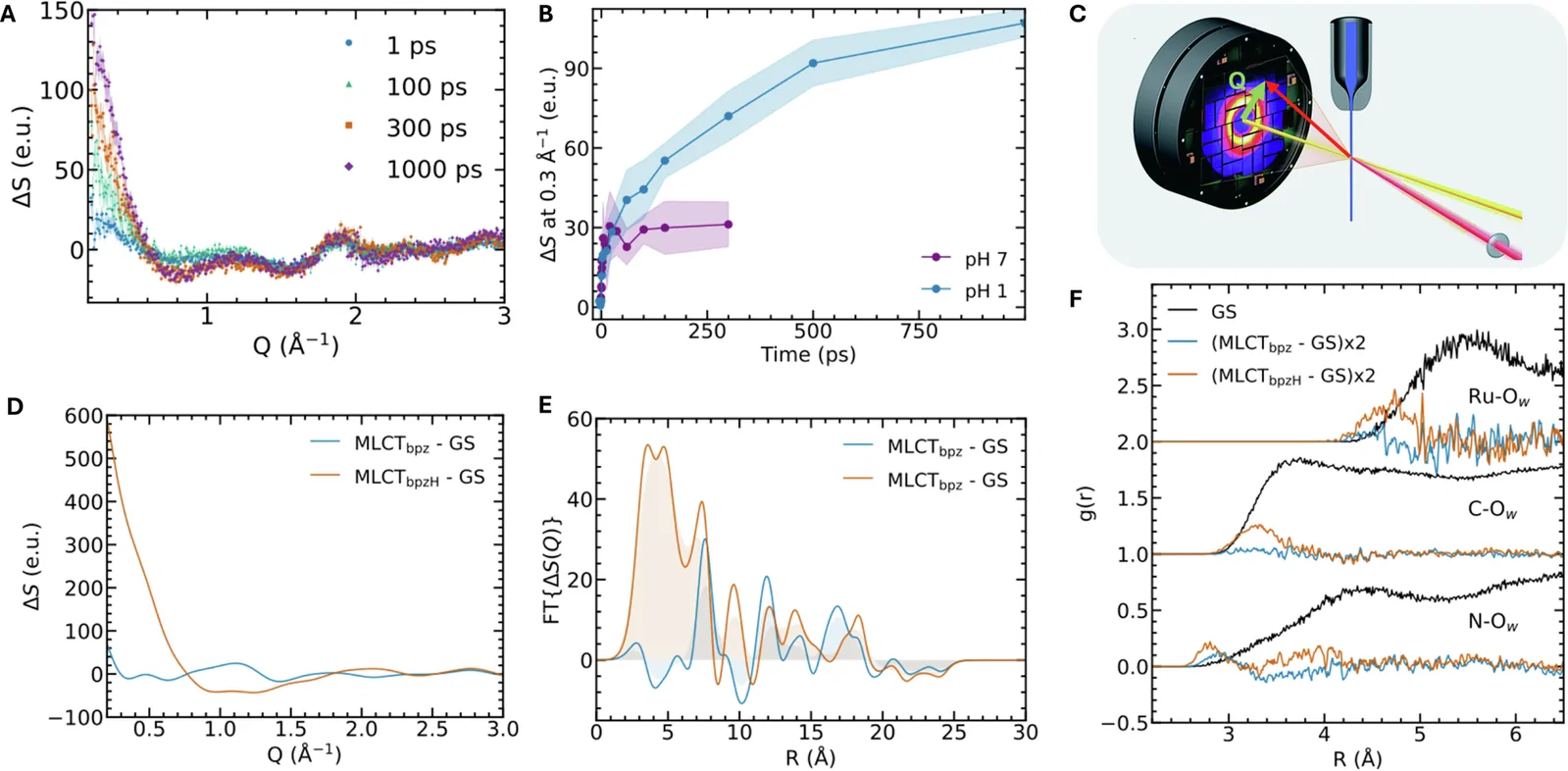
Ultrafast X-ray scattering reporting on solvent reorganization upon photoexcitation of [Ru(bpy)_2_(bpz)]^2+^. **A** Experimental difference scattering signals (*ΔS*) measured at pH 1 at 1, 100, 300 and 1000 ps following photoexcitation of [Ru(bpy)_2_(bpz)]^2+^, showing a growing low-Q feature on the timescale of protonation. **B** Kinetic traces of the low-Q scattering signal (integrated over 0.25-0.35 Å^−1^) at pH 1 (blue) and pH 7 (magenta) with 95% confidence interval calculated from the standard deviation across 5 independent runs (pH 7) and 10 independent runs (pH 1). **C** Schematic illustration of the experimental setup. Following 536 nm excitation, elastic X-ray scattering was recorded on a forward detector and radially integrated as a function of the scattering vector Q. Figure adapted with permission from ref. ^90^, RSC. **D** Simulated difference scattering curves for the ^3^MLCT_bpz_ (blue) and ^3^MLCT_bpzH_ (orange), reproducing the low-Q increase upon protonation. **E** Fourier transform of the simulated scattering curves shown in panel (D), highlighting the largest differences between the protonated and unprotonated states in the 0 < R < 5 Å region. The solid line represents the Fourier transform over a wide Q range (0-10 Å^−1^), while the shaded region shows the transform obtained using a limited Q range (0-3.0 Å^−1^), with the upper Q limit restricted by the experiment. The comparison demonstrates that the available Q range retains sensitivity to changes in the first solvation shell of the complex, although contributions from distinct solute–solvent atom pair distances cannot, in general, be resolved. **F** Radial distribution functions (RDFs) between electron-rich solute atoms (Ru, N, and C) and water oxygens (O_w_), calculated for the ground state (GS, black), MLCT_bpz_, and MLCT_bpzH_ states. The MLCT_bpz_ and MLCT_bpzH_ curves are shown as differences relative to the ground state, scaled by a factor of 2 and smoothed using a 3-point average. Each set of curves is vertically offset by 1 for clarity.

To complement the electronic insights from OTA and N K-edge XAS, we performed a separate TR-XSS experiment (Fig. 3C). Figure 3A shows transient scattering curves measured at 1 ps, 100 ps, 300 ps and 1 ns after 536 nm excitation of [Ru(bpy)_2_(bpz)]^2+^ at pH 1. At this wavelength, we directly excite the MLCT state localized on the bpz ligand and deposit less excess energy into the solvent compared to 400 nm excitation, which is preferred for TR-XSS experiments. As discussed above, the excited-state dynamics on picosecond and longer timescales are independent of excitation wavelength (Suppl. Fig. 4).

As established in previous studies^27,45^, transient XSS signals arise from photoinduced changes in intra-molecular (solute–solute) and inter-molecular (solute–solvent) atomic pair distances, as well as structural changes in the bulk solvent due to transient heating from excited-state relaxation. The latter contribution was subtracted using standard procedures^46^. As typical for Ru-polypyridyl complexes and confirmed by TDDFT (Suppl. Note 6.1), the intra-molecular bond distances of [Ru(bpy)_2_(bpz)]^2+^ remain nearly unchanged between the ground-state and lowest ^3^MLCT_bpz/bpzH_ states. Thus, the observed transient scattering signal mainly reflects changes in solute–solvent distances, indicating reorganization of the solvation shell.

At 1 ps, the signal captures the initial solvent reorganization upon population of the ^3^MLCT_bpz_ state. The positive transient at low momentum transfer (Q) suggests a shortening of solute–solvent distances, as previously observed^27–29,47^, likely reflecting strengthened hydrogen-bonding between the solvent and the reduced bpz ligand^48^. Notably, the low-Q transient signal continues to grow over 1-1000 ps, consistent with the timescale of excited-state protonation. This continued growth at pH 1 is further shown in the kinetic trace in Fig. 3B. In contrast, at pH 7, the low-Q signal rapidly plateaus after an initial rise. These observations support assigning the evolving low-Q signal to structural changes driven by excited-state protonation and are consistent with increased local electron density relative to the ground and unprotonated states.

To further interpret the measured difference scattering signals, we performed classical molecular dynamics (MD) simulations, which provide the statistical sampling necessary for an accurate description of solvation dynamics. Solute–solvent radial distribution functions (RDFs) and corresponding difference scattering signals were calculated for the ground-state ^1^GS, ^3^MLCT_bpz_, and ^3^MLCT_bpzH_ state, following an established procedure^49^. As shown in Fig. 3D, the calculated transient scattering signals reproduce the increase in the low-Q positive feature upon protonation. Figure 3E shows the Fourier transform of the simulated difference scattering curves, providing a real-space representation of how scattering-weighted atom-atom pair correlations vary as a function of interatomic distance (R). Positive contributions correspond to an increase in correlations at a given distance, whereas negative contributions indicate a reduction. The protonated state shows a marked enhancement of correlations in the 0 < R < 5 Å region relative to both the ground and ^3^MLCT_bpz_ states. This indicates that the calculated (and measured) signal is primarily sensitive to solute-solvent distances within the first solvation shell.

Having verified that the simulations reproduce our experimental observables, we next examine the changes in RDFs relative to the ground state to gain atomistic insight into solvation-shell changes across the three electronic states. Figure 3F shows the difference RDFs of atom pairs expected to contribute most strongly to the scattering signal based on their electron density and abundance: N-O_w_, C-O_w_ and Ru-O_w_, where N and C comprises all N and C atoms of [Ru(bpy)_2_(bpz)]^2+^, while O_w_ represents the oxygen atoms in water.

In the ^3^MLCT_bpz_ state, a small positive feature appears in the N-O_w_ transient RDF at 2.8 Å, consistent with strengthened hydrogen bonding to the reduced bpz ligand. Only minor changes are observed in the Ru-O_w_ and C-O_w_ RDFs, indicating limited solvation-shell changes in the MLCT state, in agreement with the weak transient signal observed experimentally and in simulations. Upon protonation, the N-O_w_ feature further increases, reflecting hydrogen-bond formation between the protonated bpz nitrogen and nearby water oxygen atoms. This corresponds to a change from N···H_w_O_w_ to NH···O_w_ interactions (see also Suppl. Fig. 17). While individual atom-pair contributions cannot be resolved experimentally, atomistic simulations provide this structural interpretation. Positive transients in the Ru-O_w_ and C-O_w_ RDFs additionally suggest a general shift of water molecules closer to the solute. This rearrangement increases the effective electron density in the first solvation shell, contributing to the enhanced low-Q signal.

Further analysis of MD simulations (Suppl. Note 6.2) indicates that, although water also approaches the bpy ligands due to their increased positive charge in both MLCT_bpz/bpzH_ states^50^, more than half of the low-Q signal arises from reorganization around the bpz ligand. In the MLCT_bpz_ state, reorientation of water near bpz C atoms – qualitatively consistent with prior reports^51^ – compensates for the strengthening of H-bonding to the bpz Ns, yielding a negligible net solute–solvent signal. This differs slightly from the small positive signal observed experimentally, a point we will examine in future work, which may require a higher level of theory to describe weak solute-solvent interactions involving the reduced bpy ligand. In the MLCT_bpzH_ state, solvent O atoms approach both C and N atoms of the bpz ligand, with most reorganization localized around the protonated pyrazine ring. This is consistent with the electronic picture revealed by the XAS measurements: in the MLCT_bpz_ state, both peripheral Ns exhibit increased interactions with the surrounding water, whereas in the MLCT_bpzH_ state, an asymmetry emerges. Here, the unprotonated bpz N retains solute-solvent interactions similar to the ground state, while significant solvation changes are localized at the protonated pyrazine ring through NH-O_w_ bonding (Suppl. Fig. 19).

In summary, these TR-XSS measurements capture, to our knowledge for the first time, solvent reorganization coupled to the steps of a PCET reaction. In the MLCT_bpz_ state, water molecules form stronger hydrogen bonds with the peripheral bpz Ns, contracting the solvation shell and producing a small, low-Q positive signal. On the protonation timescale, the low-Q signal further increases, reflecting enhanced electron density around the solute driven by hydrogen-bond rearrangements at the protonated pyrazine ring. These hydrogen-bonding rearrangements occur faster than the protonation itself, which is diffusion-limited. Nonetheless, this study demonstrates the potential of TR-XSS to probe solvent dynamics coupled to PCET. Future ultrafast experiments, where proton transfer and solvent motion are comparable in timescale, could directly reveal, for instance, whether hydrogen-bond strengthening precedes proton transfer, offering mechanistic insight into solvent-mediated regulation of the reaction.

## Discussion

To capture the coupled reorganization of electronic density and solvent in a model PCET reaction at the atomic scale, we combined femtosecond optical spectroscopy, site-specific N K-edge X-ray absorption, and time-resolved X-ray scattering.

In [Ru(bpy)_2_(bpz)]^2+^, OTA shows that the optically populated ^1^MLCT_bpy_ and ^1^MLCT_bpz_ states rapidly decay to a single ^3^MLCT_bpz_ state. At pH 7, this triplet state is long-lived (>1.4 ns), whereas at pH 1, spectral reshaping with a 460 ps time constant reflects diffusion-limited protonation of the excited state.

Ultrafast N K-edge spectroscopy and TR-XSS reveal kinetics consistent with OTA, providing a site-specific view of electronic structure and solvation. TDDFT analysis shows the photoexcited electron in the ^3^MLCT_bpz_ state is delocalized over the bpz ligand, with slight preferential localization on the coordinated Ns. Ultrafast XSS, supported by MD simulations, captures concomitant shortening of solute-solvent distances, driven mainly by enhanced hydrogen-bonding between the peripheral bpz Ns and water molecules.

In the ^3^MLCT_bpzH_ state, the combination of N K-edge XAS and TDDFT demonstrates that protonation of the ^3^MLCT_bpz_ state occurs at one peripheral N. Less intuitively, we show that protonation results in marked localization of the photoexcited electron on the protonated pyrazine ring, while the non-protonated peripheral N exhibits near-ground-state behavior. XSS and MD further highlight the resulting asymmetric electronic distribution, showing the transition from N···HO to NH···O interactions at the protonated nitrogen, whereas the unprotonated bpz N retains solute-solvent interactions similar to the ground state.

Together, these measurements provide the first integrated view of electronic and solvent reorganization during a model PCET reaction. We show that time-resolved X-ray methods provide additional insights into a well-characterized PCET system: TR-XAS yields an orbital-level description of electronic redistribution, while TR-XSS directly probes solute-solvent structural changes. The X-ray experiments were performed at 120 Hz, and recent upgrades to kHz repetition rates will significantly improve signal-to-noise and enable more advanced experiments, including resonant inelastic soft X-ray scattering and broader applicability of XSS beyond heavy-atom systems. This increasing ability to spectrally distinguish the charge-transfer and protonation steps with site-specificity and direct structural sensitivity offers a compelling proof of concept for addressing long-standing mechanistic questions, including synchronicity of electron and proton motion in concerted PCET, as well as the specific role of solvent in gating such processes. Our multimodal framework establishes a path for future studies of more complex PCET mechanisms as X-ray capabilities continue to advance, and to inform the rational design of PCET in catalysis, artificial photosynthesis, and biological energy conversion.

## Methods

### Sample preparation

For the preparation of [Ru(bpy)_2_(bpz)](PF_6_)_2_, a mixture of Ru(bpy)_2_Cl_2_ (9.85 g, 20.3 mmol) and 2,2'-bipyrazine (3.60 g, 22.8 mmol) in ethylene glycol (100 mL) was heated at 120 ^∘^C under an Ar atmosphere for 16 h. The reaction mixture was allowed to cool, then directly loaded onto a preparative column (SiO_2_, acetone). Residual 2,2'-bipyrazine was eluted using a 9:1 acetone/water mixture, then [Ru(bpy)_2_(bpz)](NO_3_)_2_ using 90:9:1 acetone/water/KNO_3_ (sat. aq.). Acetone was removed under reduced pressure, then KPF_6_ (sat. aq.) was added. The resultant precipitate was filtered, washed with water, then diethyl ether to afford the title compound as a red solid (12.6 g, 72%).

^1^H NMR (400 MHz, CD_3_CN): *δ* 9.72 (d, *J* = 1.3 Hz, 2H), 8.54–8.50 (m, 6H), 8.11 (tt, *J* = 8.0, 1.4 Hz, 4H), 7.86 (dd, *J* = 3.3, 1.2 Hz, 2H), 7.70 (d, *J* = 5.7 Hz, 2H), 7.67 (d, *J* = 5.6 Hz, 2H), 7.47–7.40 (m, 4H) ppm.

For the preparation of [Ru(bpy)_2_(bpz)]Cl_2_, [Ru(bpy)_2_(bpz)](PF_6_)_2_ (6.00 g, 6.96 mmol) was eluted through an Amberlite^®^ IRA-400 chloride ion-exchange column with 10% acetonitrile in methanol, and the solvent removed under reduced pressure to afford the title compound (4.47 g, quant.). The ^1^H NMR spectrum matches previous literature reports^52^.

^1^H NMR (400 MHz, D_2_O): *δ* 9.78 (d, *J* = 1.2 Hz, 2H), 8.59 (d, *J* = 8.3 Hz, 4H), 8.53 (d, *J* = 3.4 Hz, 2H), 8.14 (td, *J* = 8.0, 1.6 Hz, 4H), 8.10 (dd, *J* = 3.4, 1.2 Hz, 2H), 7.79 (ddd, *J* = 5.6, 1.6, 0.7 Hz, 2H), 7.75 (ddd, *J* = 5.6, 1.6, 0.7 Hz, 2H), 7.47–7.42 (m, 4H) ppm.

### Optical Transient Absorption

The setup for fs transient absorption is described in ref. ^53^. In brief, the output of an amplified Ti:Sapphire system (Solstice Ace, Spectra-Physics), producing 35 fs pulses centred at 800 nm with a 5 kHz repetition rate was split into two to produce pump and probe pulses. The probe pulses with  ~ 1 µJ energy were chopped to a repetition rate of 1 kHz, and focused onto a 3 mm CaF2 plate to generate a white-light continuum. These pulses were focused onto the sample to 60 µm diameter spot, and overlapped with the 400 nm pump pulses at 500 Hz (of 100 µm diameter spot) generated by doubling the fundamental with a BBO crystal. Excitation at 500 nm was achieved by doubling the output of a TOPAS-Prime combined with a NirUVis frequency mixer (both from Light Conversion), and keeping the same pump fluence at the sample position. The pump fluence on the sample was 0.25 mJ cm^−2^. All samples were measured at the magic angle in 1 mm quartz cuvettes, and flushed with nitrogen during the entire measurement time. The solvents used for sample preparation were water (purified using a Milli-Q system, Millipore) and a standard solution of 1 M HCl (Fluka). For the KIE measurements, a 0.1 M deuterium chloride solution (prepared dissolving 35 wt. % in D_2_O, ≥99 atom % D in deuterium oxide, 99.9 atom % D) was used. The solutions had a range of concentrations from 1 × 10^−5^ M (10 µM) to 2 × 10 ^−5^ M (20 µM).

### N K-edge x-ray absorption

Time-resolved N K-edge X-ray Absortion Spectroscopy (XAS) measurements of aqueous [Ru(bpy)_2_(bpz)]^2+^ were collected at the ChemRIXS end-station of the Linac Coherent Light Source (LCLS) at the SLAC National Accelerator Laboratory.

The instrument is equipped with a recirculating liquid jet delivery system that utilizes microfluidic nozzle as described in ref. ^54^. We used a colliding nozzle geometry to produce a flat jet of approximately 6 µm thickness, as confirmed by comparing transmission measurements with and without the sample. [Ru(bpy)_2_(bpz)]^2+^ in acidic water was flowed at 3.7 mL min^−1^ with an HPLC pump. Evaporative losses were compensated by adding 0.11 mL min^−1^ of water to the sample reservoir. The sample concentration and pH of the solution were regularly checked during a shift to ensure values between 65 and 75 mM and between 1 and 1.1 for concentration and pH, respectively.

400 nm, 50 fs, 4 µJ (22 mJ/cm^2^) pump pulses (second-harmonic of an 800 nm Ti:sapphire system) were focused to a 150 µm (FWHM) spot at 60 Hz. Relative arrival times of pump and XFEL pulses were monitored upstream with an arrival time monitor. A time jitter of  ~ 600 fs was observed.

LCLS delivered 20 fs X-ray pulses at 120 Hz. The energy was scanned across the N K-edge by synchronously tuning the undulator and a grating monochromator (40 µm exit slit, 0.16 eV bandwidth, scan rate 0.5 µrad s^−1^). Pulse energy and spot size at the sample were 4.18 µJ and 40 µm, respectively. After the sample, the transmitted X-rays pass through a 1.25 μm Co foil and is recorded by an Andor CCD camera (Andor Newton SO DO940P). The incident flux (*I*_0_) was monitored via mirror-fluorescence MCP detectors (FIMs) situated above the KB optics.

The absolute energy was calibrated by setting the high-energy XAS peak of [Ru(bpy)_2_(bpz)]^2+^ to a value of 399.5 eV, in agreement with the published N K-edge peak position of [Ru(bpy)_3_]^2+^^41^.

Shot-to-shot one-dimensional projections of the CCD images recording transmitted X-ray were utilized to calculate integrated transmitted intensity over a region of interest, with (*I*_on_) and without (*I*_off_) optical pumping.

To improve the correlation between the incoming X-ray intensity (*I*_0_) measured on the FIM detectors and the signal from the Andor transmission detector, we used a linear regression model. This process was only applied to data collected between 393-394 eV, to avoid distortions due to sample absorption. The result is a set of coefficients that were then utilized to predict *I*_0_ based on the FIM measurements at all incident X-ray energies. By using this linear regression method, the S/N was improved by roughly 4 times as shown in ref. ^43^.

*I*_on_, *I*_off_ and *I*_0_ were binned according to the incident X-ray energies in bins of 0.1 eV, and the data normalized as:

$${\hat{I}}_{{{\rm{off/on}}}}=\frac{{I}_{{{\rm{off/on}}}}}{{I}_{0}}$$

Finally the total and transient XAS spectra were calculated as

$${A}_{on/off}=-\log \left(\frac{{\hat{I}}_{{{\rm{off/on}}}}}{{I}_{0}}\right),$$

and

$$\Delta A=-\log \left(\frac{{\hat{I}}_{{{\rm{off}}}}}{{\hat{I}}_{{{\rm{on}}}}}\right),$$

respectively. A linear background constructed by fitting the pre-edge region was further removed from the total XAS spectra. Error bars (95 % confidence interval) were obtained from the standard deviation of averaging individual scans.

### X-ray solution scattering

Ultrafast X-ray solution scattering experiments (XSS) were performed at the X-ray Correlation Spectroscopy (XCS) instrument at the LCLS using the Liquid Standard Configuration^55,56^. A 25 mM aqueous solution of [Ru(bpy)_2_(bpz)]^2+^ was pumped through a cylindrical capillary with a 50 µm inner diameter using a HPLC pump. The flowing jet was excited at 536 nm and the solution was recirculated continuously during the experiment. The neutral pH data were collected at a laser power of 4 µJ (72 mJ/cm^2^), while the pH=1 data were collected at a laser power of 2 µJ (36 mJ/cm^2^). The experimental chamber was kept under a helium atmosphere to minimize air scattering.

The sample was probed with self-amplified stimulated emission (SASE) X-ray pulses (45 fs FWHM, 17 µm spot size) at 8 keV in pink beam (non-monochromatized) and at a repetition rate of 120 Hz. The pump speed for the liquid jet was chosen such that a fresh sample was hit for every probe event. For each individual pump-probe event, the time delay between the laser and the X-ray pulse was determined using a timing tool. The pulse sequence was chosen so that every 7th event probed the unexcited sample (laser drop) and every 101st event records the signal in the absence of X-rays (X-ray drop); consequently, every 707th event constituted a measurement of the experimental background in the absence of laser or X-ray light (laser and X-ray drop). These reference measurements were used as a ground-state reference for the calculation of pump-probe difference signals and subtract any detector background.

The XSS data were recorded using an epix10k2M detector in forward scattering geometry. Two-dimensional scattering images were corrected for X-ray polarization and solid angle coverage. One-dimensional isotropic scattering curves were obtained following a previously reported routine^57^. Several corrections were performed to improve the signal-to-noise ratio during further processing of the reduced data: the scattering intensity was corrected for non-linearity in both X-ray energy and the total integrated detector intensity; the total integrated detector intensity was correlated with upstream intensity monitors and events with more than 5 standard deviations outside their linear correlation were filtered out; a total of 1% of shots at the high or low end of total detector intensity were filtered out; transient signals for each laser-on event were calculated with respect to its 100 closest laser-off events to mitigate temporal drifts. Additionally, outliers in time tool parameters (edge jump position, FWHM) were removed to improve the time resolution. The obtained scattering signals were scaled to the scattering signal calculated from a liquid unit cell, containing one [Ru(bpy)_2_(bpz)]^2+^ molecule and 2220 H_2_O molecules. This scaling procedure yields scattering signals in electron units per solute molecule.

### QM/MM calculations

All QM/MM and TDDFT calculations were performed with the NWChem computational chemistry program^58–60^. All basis sets were obtained from the EMSL Basis Set Exchange^61–63^. The 3D molecular model of the [Ru(bpy)_2_(bpz)]^2+^ and its protonated form was solvated in a cubic water box of side 53Å. The 3D molecular model was prepared using the preparation tools and a pre-prepared equilibrated bulk water template available in the NWChem software. To balance the charge of the Ru complex, chloride ions were added in the solvation box. The Ru complex was assigned as the quantum mechanics (QM) region and solvent molecules as well as two chloride ions were assigned as the molecular mechanics (MM) region in the subsequent QM/MM calculations. The extended single point charge water molecule (SPC/E) force-field^64^ was used as the classical force-field. Fe^2+^ van der Waals parameter from CLAYFF^65^ force-field was assigned for the Ru atom of the complex. The system was optimized with the NWChem QM/MM module^66–68^ using B3LYP^69^ exchange-correlation functional and def2-TZVP basis set for Ru atom^70,71^ and 6-31G(d) basis set for C, N, H atoms^72–74^ in the QM region. As described by the SPC/E potential, the SHAKE algorithm^75^ was only applied to the water molecules to constrain the bond lengths and bond angle. The Coulombic interactions cut off was set to 1.2 nm. The QM/MM interaction zone surrounding the complex was set to 1.2 nm. After creation of the molecular model, QM/MM calculations were performed for the ground-state, ^3^MLCT and protonated ^3^MLCT states. Geometry optimization was performed in a cyclic way with a maximum of 100 QM region Broyden-Fletcher-Goldfarb-Shanno (BFGS) iterations^76^ followed by a maximum of 3000 MM region steepest-descent (SD) iterations. After the optimization, QM/MM dynamics was performed. First, the water solvent was allowed to equilibrate while the complex held fixed over 10 ps with a time step of 2 fs and using the Berendsen’s thermostat^77^ for NVT simulations at 298.15 K. After the solvent equilibration step, the complex was allowed to equilibrate with the solvent for 1 ps and a time step of 0.25 fs. After the equilibration process, QM/MM dynamics of the entire system were ran.

### TDDFT calculations

TDDFT calculations were performed on 20 QM/MM snapshots of the ground-state (S_0_), ^3^MLCT_bpz_ and protonated ^3^MLCT_bpzH_ states of [Ru(bpy)_2_(bpz)]^2+^ within QM/MM framework^29,78,79^ using B3LYP^69^ exchange-correlation functional, def2-TZVP basis set for Ru^70,71^ and 6-31G(d) basis set for C, N and H^72–74^. The choice of functionals and basis set was based on prior work on related ruthenium polypyridyl complexes^80,81^, and validated by agreement between computed and experimental UV-vis and X-ray observables. For calculating K-edge XAS for N centers, restricted energy window (REW) approach^82–84^ was used, where excitations were only allowed from nitrogen 1s orbitals. Discrete transitions (roots) have been convolved with 0.25 eV (FWHM) Lorentzian functions to generate the spectra. A global shift of 11.75 eV is applied to the calculated spectra. When plotting discrete roots and the continuous spectrum together, a global scaling factor (0.04) is applied to the root intensities. The spectra shown in the main text are constructed by a weighted average of the 20 QM/MM snapshots, with weights constructed based on calculated total DFT energy as illustrated in Suppl. Fig. 12. Spin density plots were generated using the structures of one of the snapshots for the ^3^MLCT_bpz_ and its protonated state. Calculations were also performed with the larger 6-311G** basis set for C, N, and H, and this did not produce a difference in the computed X-ray spectra.

### Classical MD simulations

For these simulations, we use the Desmond software package developed at D. E. Shaw Research ^85^. DFT-optimized structures of the S_0_, ^3^MLCT_bpz_ and ^3^MLCT_bpzH_ states were obtained from QM/MM snapshots and solvated in a water box. The solute structures were kept frozen during 2 ns simulations, allowing the solvent to respond to the altered electronic distribution of each state. The complex is frozen by applying harmonic positional restraints with force constant of 500 kcal/mol, and solvated in a cubic box with 50 Å side length containing 4011 water molecules using the TIP4P-Ew potential ^86^. Changes in solute partial charges, computed at the electrostatic potential (ESP) level, were used to describe the Coulombic interactions involving atoms of the solute, while other nonbonded interactions are modeled with the Lennard-Jones potential using parameters for the complex from the OPLS2005 force field ^87^. The propagation is performed in the NVT ensemble using a Nose-Hoover thermostat at 300 K, with a time step of 1 fs for a total length of 2 ns. The cut-off for the complete treatment of the coulombic interactions was set to 9 Å, and the Ewald summation method was used for the long-range electrostatic interaction. From the resulting trajectories, radial distribution functions (RDFs) were computed and used to simulate difference scattering signals following established procedures from the literature^49^. The RDFs are sampled in 0.01 Å using the VMD software^88^.

## Supplementary information

Supplementary Information

Transparent Peer Review file

## Source data

Source Data

## Data Availability

Source data are provided with this paper. Large datasets are available in a Figshare repository^89^. Additional experimental and theoretical data supporting the findings of this study are available from the corresponding authors. Raw X-ray spectroscopy and scattering data generated at the LCLS facility are stored on the facility’s archive servers and are available upon request from the corresponding authors. Source data are provided with this paper.
