# Supplementary material for: Electronic and solvent reorganization in proton-coupled electron transfer captured by ultrafast X-rays
Source: Nat Commun. 2026 Aug 26;17:9053. doi: 10.1038/s41467-026-75943-4 (PMC13518934; doi:10.1038/s41467-026-75943-4)
Supplement: Supplementary file 1 [file 41467_2026_75943_MOESM1_ESM.pdf]

Supplementary Information: Electronic and Solvent Reorganization in Proton-Coupled Electron Transfer Captured by Ultrafast X-rays

Abdullah Kahraman[1,2], Michael Sachs[2], Soumen Ghosh[1,a], Benjamin I. Poulter[3], Estefanía Sucre-Rosales[4], Elizabeth S. Ryland[2], Douglas Garratt[2], Sumana L. Raj[2], Natalia Powers-Riggs[2], Subhradip Kundu[4], Christina Y. Hampton[5], David J. Hoffman[5], Giacomo Coslovich[5], Georgi L. Dakovski[5], Patrick L. Kramer[5], Matthieu Chollet[5], Roberto Alonso-Mori[5], Tim B. van Driel[5], Sang-Jun Lee[6], Kristjan Kunnus[5], Amy A. Cordones[2], Robert W. Schoenlein[2,5], Eric Vauthey[4], Amity Andersen[7], Niranjan Govind[1,3], Christopher B. Larsen *[8], and Elisa Biasin †[1,2]

[1]Physical Sciences Division, Physical and Computational Sciences Directorate, Pacific Northwest National Laboratory, Richland, WA 99354, USA
[2]Stanford PULSE Institute, SLAC National Accelerator Laboratory, Menlo Park, CA 94025, USA
[3]Department of Chemistry, University of Washington, Seattle, Washington 98195, USA
[4]Department of Physical Chemistry, University of Geneva, CH-1211 Geneva, Switzerland
[5]Linac Coherent Light Source, SLAC National Accelerator Laboratory, Menlo Park, CA 94025, USA.
[6]Stanford Synchrotron Radiation Lightsource, SLAC National Accelerator Laboratory, Menlo Park, CA 94025, USA.
[7]Environmental Molecular Sciences Laboratory, Earth and Biological Sciences Directorate, Pacific Northwest National Laboratory, Richland, WA 99354, USA
[8]School of Chemical Sciences, University of Auckland, Auckland 1010, New Zealand
[a]Present Address: Department of Chemistry, Indian Institute of Technology Madras, Chennai, 600036 India

Contents

1 Protonation Conditions for the ^{3}MLCT State **2**

2 Additional Data and Analysis: OTA **3**
- 2.1 OTA data and global analysis . . . 3
- 2.2 Wavelength dependence . . . 4
- 2.3 Dependence on pH and isotope effects . . . 4
- 2.4 TDDFT calculations of valence excitations . . . 5

3 Additional Data and Analysis: N K-edge XAS **6**
- 3.1 Transient Nitrogen K-Edge Response at neutral pH . . . 6
- 3.2 Reconstruction of the $^3MLCT_{bpz}$ N K-edge Spectrum . . . 7

*Corresponding author: christopher.larsen@auckland.ac.nz
†Corresponding author: elisa.biasin@pnnl.gov

4 Additional Data and Analysis: XSS data **8**
4.1 Bulk solvent heating 8
4.2 Neutral pH XSS data 9

5 Transition-Specific TDDFT Analysis of Nitrogen K-Edge X-ray Absorption Spectra **10**
5.1 Orbitals Visualization and Analysis 12
5.2 Site–resolved N K-edge Excitations 16

6 Additional structural insights from MD simulations **19**
6.1 Intramolecular Structural Changes 19
6.2 Individual solute–solvent contributions 20

1 Protonation Conditions for the ^{3}MLCT State

To identify conditions under which the $^3MLCT_{bpz}$ state of $[Ru(bpy)_2(bpz)]^{2+}$ undergoes protonation while the ground-state remains unprotonated, we examined previously reported spectroscopic pH titrations. Prior studies utilizing UV-Vis electronic absorption and emission spectroscopy have determined that the ground-state of $[Ru(bpy)_2(bpz)]^{2+}$ exhibits a pKa of -0.15,[1] whereas the ^{3}MLCT excited-state has a pKa* of 3.5.[2] Based on these reported pKa values, the pH at which the maximum populations of both the unprotonated ground-state and the protonated excited-state coexist is approximately 1.67. A pH range of 1–2 ensures an acceptable population ($> 95\%$) of both states (Suppl. Fig. 1). Consequently, we selected two experimental conditions: distilled water (nominal pH 7), where $[Ru(bpy)_2(bpz)]^{2+}$ remains unprotonated in both the ground and excited-states, and 0.1 M HCl (nominal pH 1), where protonation occurs selectively in the excited-state while the ground-state remains unprotonated.

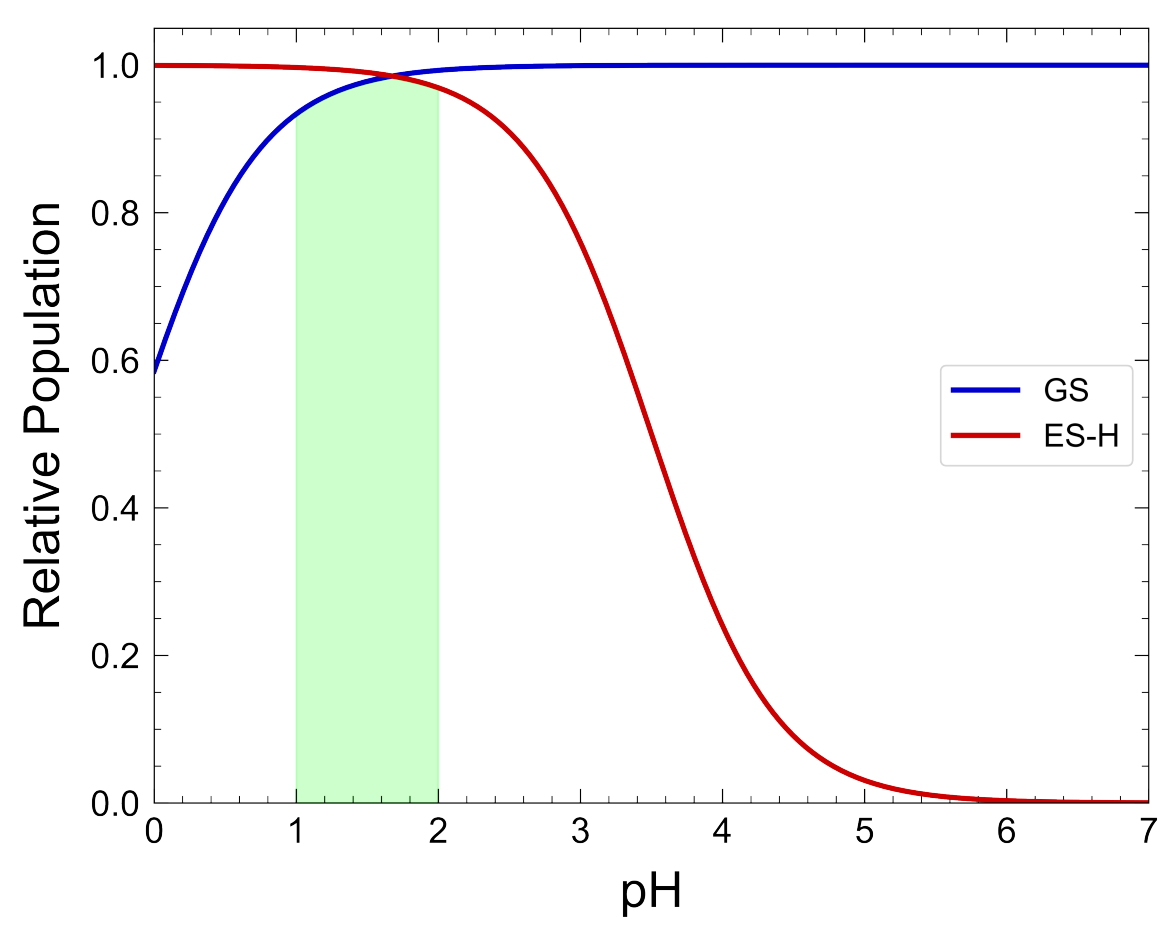

Supplementary Figure 1: Relative populations of ground-state (GS, unprotonated) and protonated excited-state (ES-H) as a function of pH based on reported pKa values.

Suppl. Fig. 2 shows that UV-Vis electronic absorption spectra of $[Ru(bpy)_2(bpz)]^{2+}$ recorded in both water and 0.1 M HCl are virtually identical, indicating minimal protonation of the ground-state. Consistent with prior reports, two distinct metal-to-ligand charge transfer (MLCT) absorption bands are observed: the higher-energy transition at 413 nm corresponds to electron transfer terminating on the bpy ligand ($MLCT_{bpy}$), while the lower-energy transition at 474 nm corresponds to electron transfer terminating on the bpz ligand ($MLCT_{bpz}$).

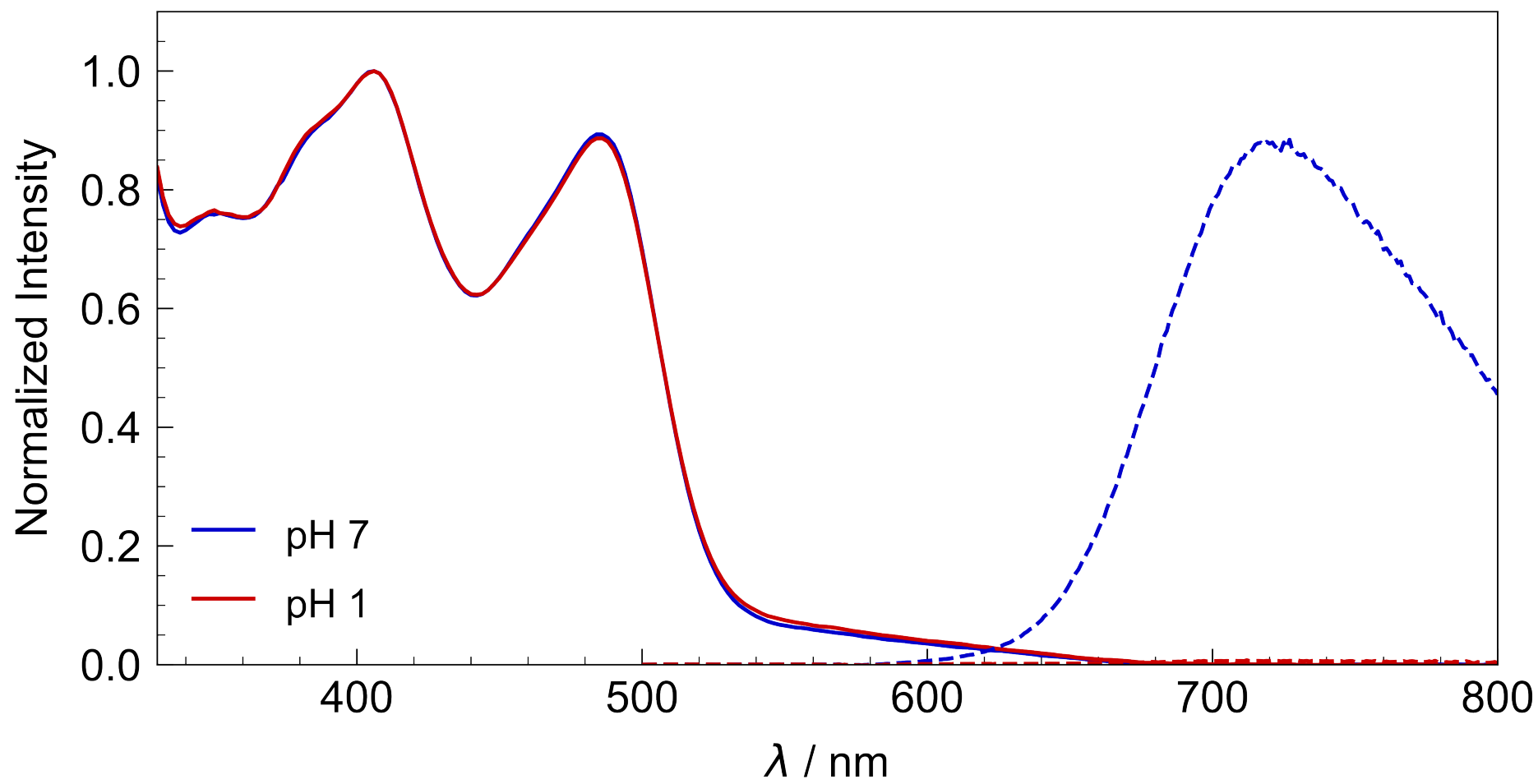

Supplementary Figure 2: UV-Vis absorption spectra (solid) and photoluminescence spectra (dashed) of $[Ru(bpy)_2(bpz)]^{2+}$ at pH 1 (red) and pH 7 (blue).

Excitation into the $MLCT_{bpz}$ transition in water results in characteristic MLCT luminescence at 719 nm. However, in 0.1 M HCl, this luminescence is almost entirely quenched (> 99%), affirming the validity of the chosen experimental conditions.

2 Additional Data and Analysis: OTA

2.1 OTA data and global analysis

The optical transient absorption (OTA) spectra of $[Ru(bpy)_2(bpz)]^{2+}$ were recorded in water and 0.1 M HCl following excitation at 400 nm, targeting the $MLCT_{bpy}$ transition. As shown in the next section, the triplet MLCT state localized on the bpz ligand ($^3MLCT_{bpz}$) is populated within our instrument response function. As shown in Suppl. Fig. 3a, the $^3MLCT_{bpz}$ state is characterized by ground-state bleaching (GSB) of both the $S_0 \rightarrow ^1MLCT_{bpy}$ and $S_0 \rightarrow ^1MLCT_{bpz}$ absorption bands, a pronounced excited-state absorption (ESA) centered at $\sim$ 350 nm, and a broad, weak ESA extending into the near-infrared. In water, this state persists well beyond the instrumental detection window ($\sim$ 1.4 ns, Suppl. Fig. 3b). This long-lived state is consistent with the reported ^3MLCT luminescence lifetime of 92 ns [3].

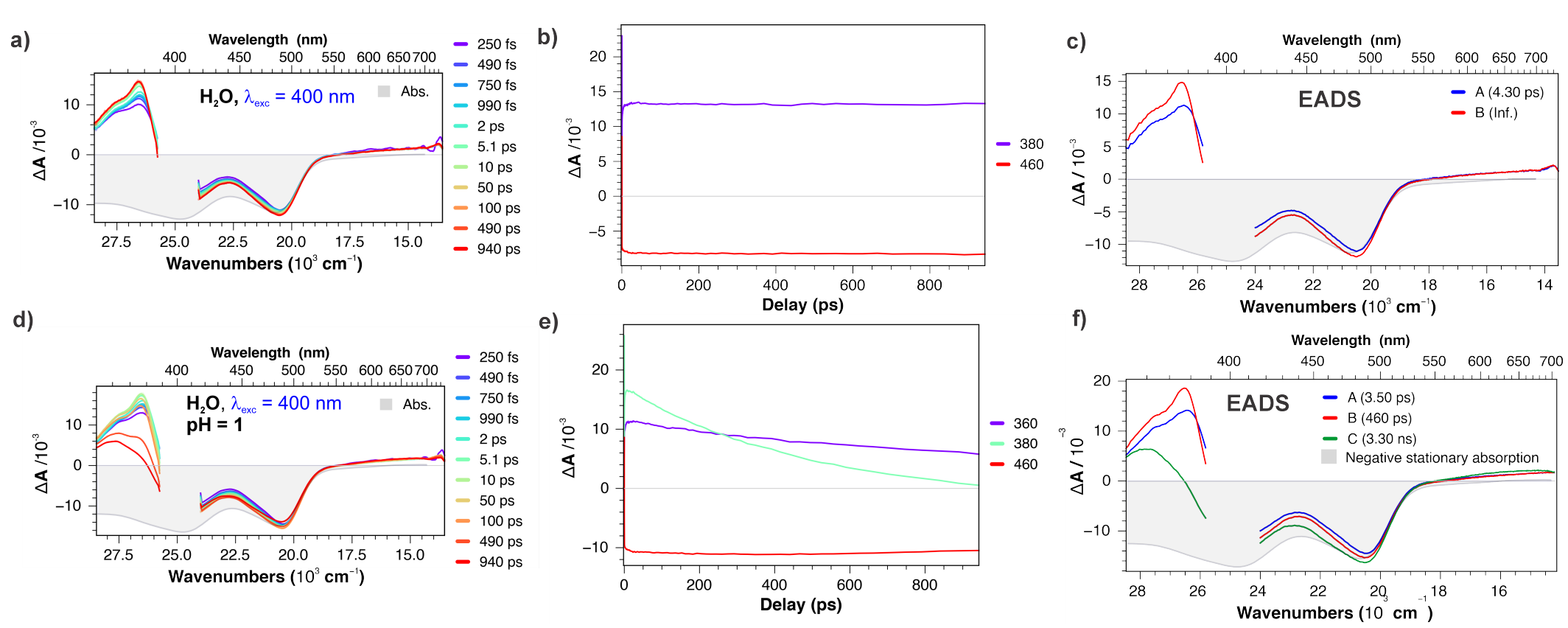

Supplementary Figure 3: Transient absorption response of aqueous $[Ru(bpy)_2(bpz)]^{2+}$ in neutral conditions (pH = 7, top) and in 0.1 M HCl (pH = 1, bottom) (a) Spectral evolution after 400 nm excitation, recorded at delays from 240 fs to 1.4 ns at pH = 7. (b) Corresponding kinetics at 375 and 460 nm. (c) Evolution-associated difference absorption spectra (EADS) extracted from global analysis. (d,e,f) Same sequence for pH = 1.

Suppl. Fig. 3d show the data recorded in 0.1 M HCl. While the initial excited-state evolution mirrors that in water, distinct spectral modifications emerge on longer timescales (10 ps – 1000 ps). Specifically, the ESA at ∼ 350 nm exhibits a reduction in intensity and shifts to higher energy, suggesting modifications in the electronic structure of the reduced bpz ligand upon protonation. Additionally, between 0.5 ns and 1.0 ns, partial ground-state recovery is observed, signifying a substantially shortened lifetime of the protonated excited-state.

Global kinetic analysis was performed using a two-step sequential model for water (Suppl. Fig. 3c), and a three-step sequential model for acidic conditions(Suppl. Fig. 3f) [4, 5]. The first step, assigned to ultrafast vibrational and solvent relaxation, occurs in a few ps. In neutral solvent, the second kinetic component extends beyond the detection window. In contrast, in 0.1 M HCl, an additional intermediate state (460 ps rise) is resolved, consistent with protonation of the bpz ligand in the excited-state. This process is followed by a subsequent decay with a lifetime of ∼ 3 ns, confirming rapid excited-state deactivation in acidic media.

2.2 Wavelength dependence

Suppl. Fig. 4 show additional OTA measurements carried out to test the wavelength dependence on the excited state dynamics of $[Ru(bpy)_3]^{2+}$. Excitation at 400 nm targets the $MLCT_{bpy}$ transition, while excitation at 500 nm targets the $MLCT_{bpz}$ transition. Both excitations yield an identical transient response, confirming ultrafast population transfer from $MLCT_{bpy}$ to $MLCT_{bpz}$ occurring on a sub-180 fs timescale, beyond the resolution of the instrumental response function.

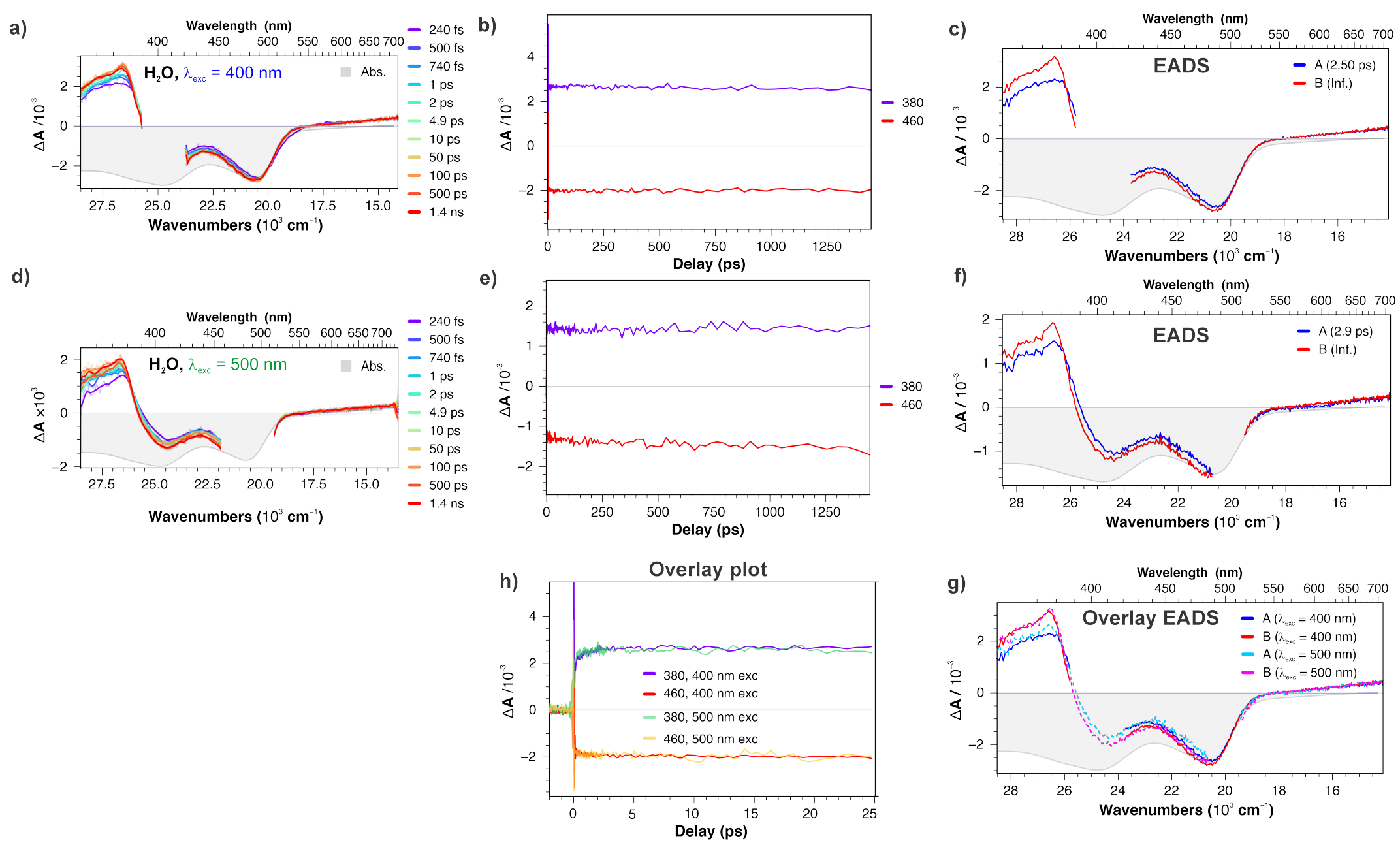

Supplementary Figure 4: Transient absorption response of aqueous $[Ru(bpy)_2(bpz)]^{2+}$. (a) Spectral evolution after 400 nm excitation, recorded at delays from 240 fs to 1.4 ns. (b) Corresponding kinetics at 375 and 460 nm. (c) Evolution-associated difference absorption spectra (EADS) extracted from global analysis. (d,e,f) Same sequence for 500 nm excitation. (g) Overlay of the EADS for the 400 nm and 500 nm data. 400 nm data have been scaled by 1.7. (h) Comparison of kinetic traces recorded upon 400 nm and 500 nm, zoomed in the first 25 ps. 400 nm data have been scaled by 1.7.

2.3 Dependence on pH and isotope effects

Suppl. Fig. 5 shows a comparison between OTA data collected as a function of pH and isotopic substitution. The protonation time constant shortens from ∼460 ps at pH 1 to ∼30 ps at pH 0, consistent with the approximately 10-fold increase in $[H^+]$, and is in line with a first-order dependence on proton concentration. At pH 1, no measurable kinetic isotope effect (KIE) is observed, while at

pH 0 a small KIE of ~ 1.3 is found, consistent with expectations for a diffusion-influenced process. The combined strong pH dependence and weak isotope effect are consistent with a diffusion-limited mechanism for formation of the protonated excited state.

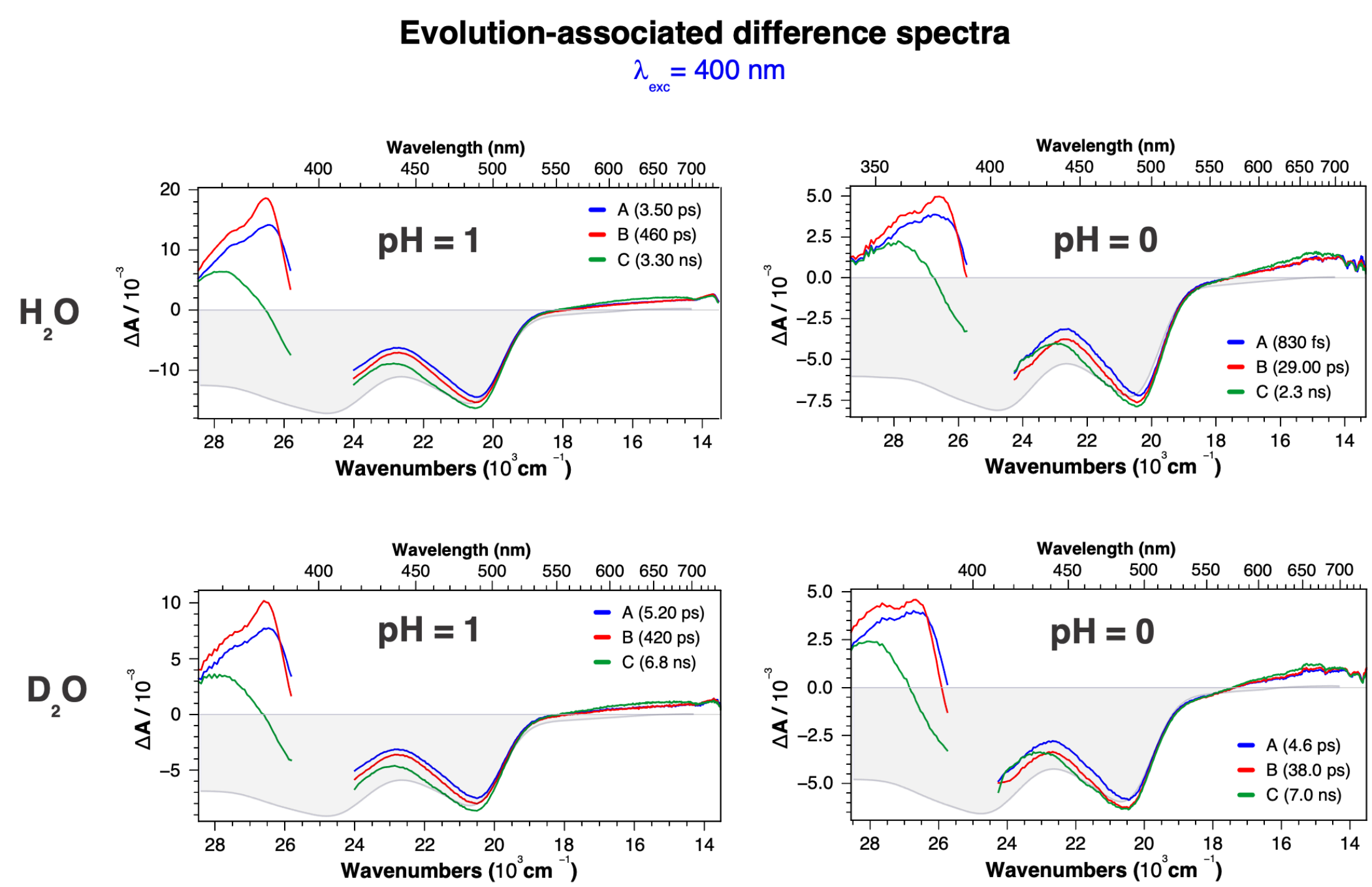

Supplementary Figure 5: Evolution-associated difference absorption spectra (EADS) obtained from global analysis of OTA data for $[Ru(bpy)_2(bpz)]^{2+}$ upon 400 nm excitation as a function of pH and isotopic substitution. Left column: pH 1; right column: pH 0. Top row: HCl/H_2O solutions; bottom row: DCl/D_2O solutions. Colored curves (A, B, C) represent spectra associated with successive kinetic components, with lifetimes from the sequential fit reported in the legends. The gray trace shows the inverted steady-state absorption spectrum for reference.

2.4 TDDFT calculations of valence excitations

UV–Vis spectra for the ground-state (^{1}GS), the lowest triplet MLCT state ($^3MLCT_{bpz}$), and the protonated triplet MLCT state ($^3MLCT_{bpzH}$) were computed using TDDFT on the same QM/MM snapshots employed for the N K-edge calculations. Suppl. Fig. 6 shows that the simulations reproduce the experimentally observed decrease in ESA intensity at 350 nm upon excited-state protonation (see Figure 1C). Interpreting these UV–Vis TDDFT spectra is difficult because the high density of closely spaced states leads to strong spectral congestion, each absorption band contains contributions from multiple transitions of comparable intensity, and the excitations are delocalized over several molecular orbitals. As a result, the excited-states have mixed character, which prevents assignment of a simple orbital picture. In contrast, the nitrogen K-edge spectra overcome these limitations: the localized N 1s orbital enforces site-specific excitation that couples to only a few unoccupied states, providing a much clearer orbital-level interpretation.

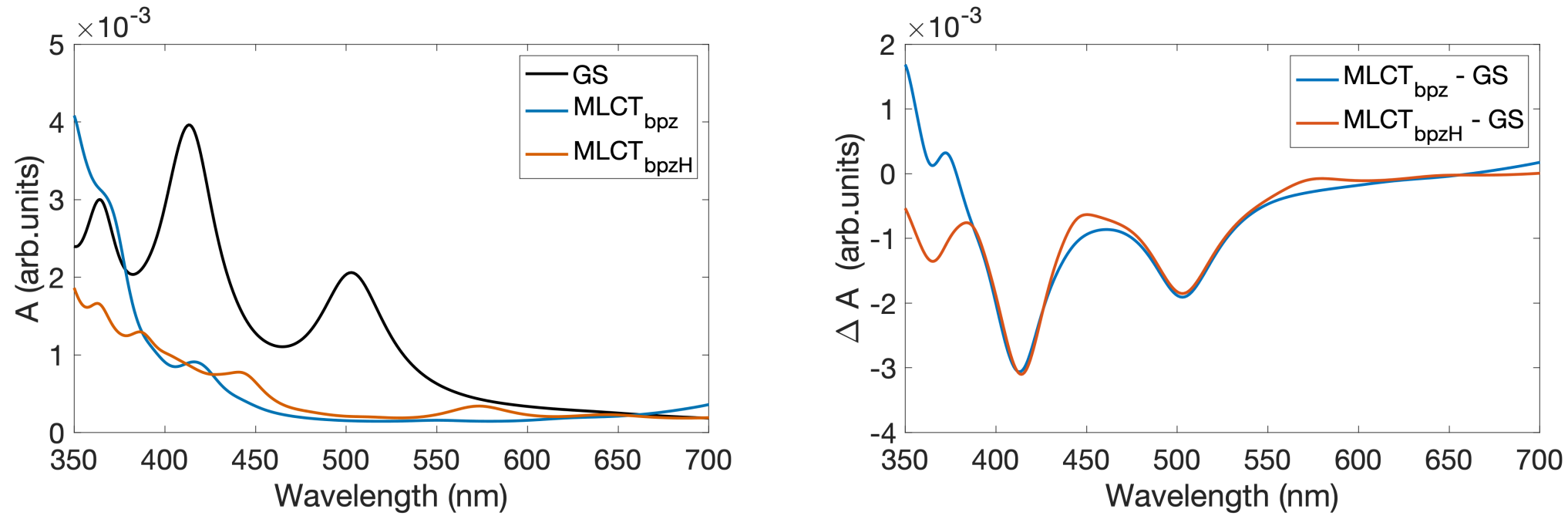

Supplementary Figure 6: (left) TDDFT calculated UvVis spectrum of the ^{1}GS, lowest ^{3}MLCT$_{\text{bpz}}$, and lowest ^{3}MLCT$_{\text{bpzH}}$). The spectra have been globally shifted by 27 nm to align the MLCT$_{\text{bpy}}$ peak with the experimental value (413 nm). (right) Transient spectra (obtained from the calculations on the right panels) reproduce the observed change upon excite-state protonation of $[Ru(bpy)_2(bpz)]^{2+}$, i.e. the decrease in intensity of the ESA at 350 nm.

3 Additional Data and Analysis: N K-edge XAS

3.1 Transient Nitrogen K-Edge Response at neutral pH

Under similar conditions described in the Methods, we recorded time-resolved N K-edge XAS spectra upon photoexcitation of $[Ru(bpy)_2(bpz)]^{2+}$ in water at neutral condition. However, this was done at the beginning of the beamtime, when experimental conditions were not optimized, and the data show a worse S/N with respect to the pH 1 data shown in the main manuscript. Suppl. Fig. 7 shows nitrogen K-edge XAS difference spectra (ΔA) of aqueous $[Ru(bpy)_2(bpz)]^{2+}$ recorded at 1ps, 100 ps, 300 ps after 400 nm excitation. Within the experimental uncertainty, the neutral solution shows no spectral evolution over the 1-300 ps time range, in agreement with population of a long-lived ^{3}MLCT state and no excited-state protonation. Suppl. Fig. 8 shows a comparison of the pH 1 and pH 7 data at each time delay, further supporting the decrease of the transient positive signal at 398.9 eV observed only under acidic conditions.

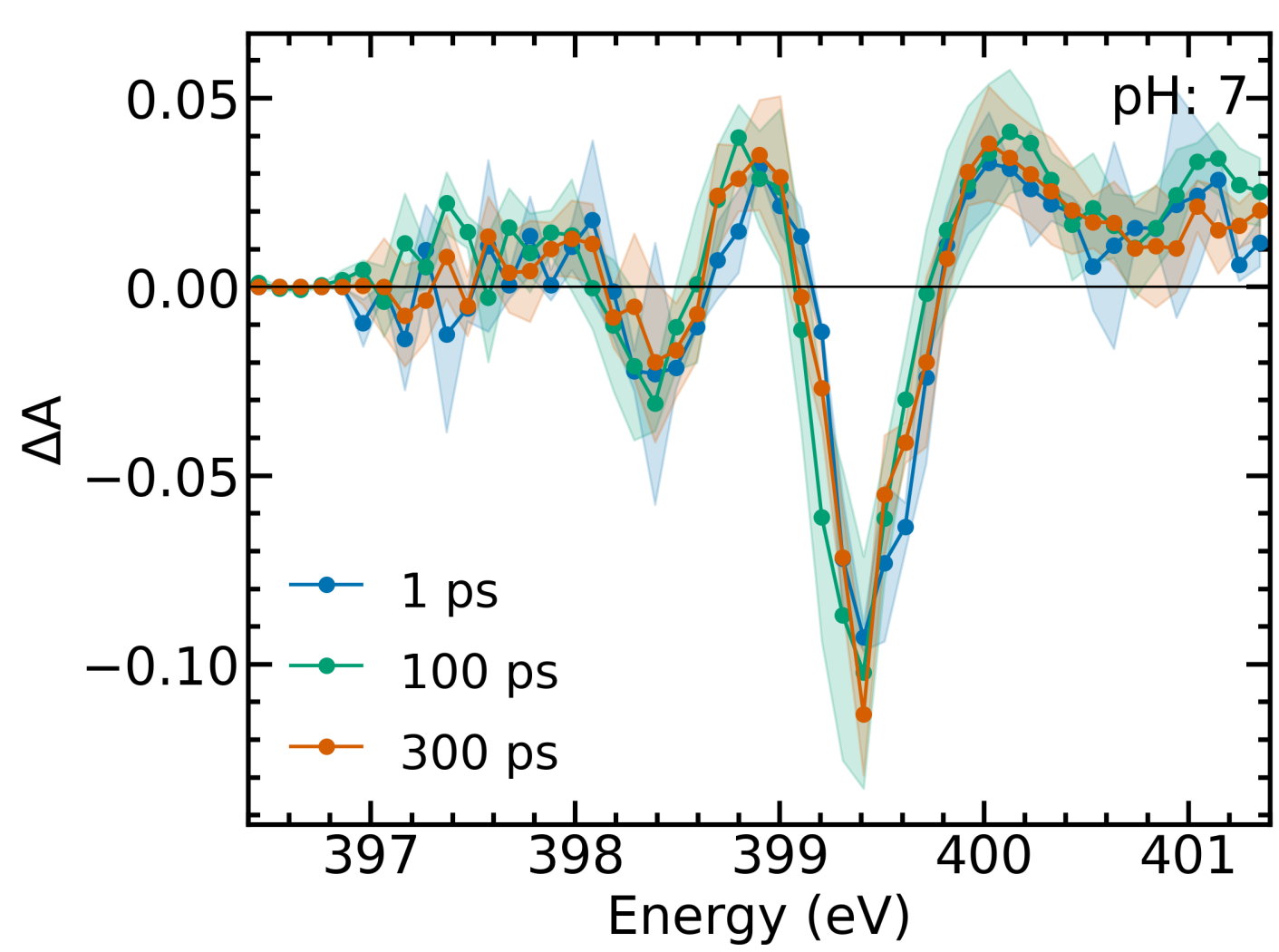

Supplementary Figure 7: Nitrogen K-edge transient-absorption difference spectra (ΔA) of $[Ru(bpy)_2(bpz)]^{2+}$ in neutral pH water at 1 ps (blue), 100 ps (green) and 300 ps (orange) after 400 nm excitation. Shaded envelopes indicate 95 % confidence intervals calculated using the standard deviation across 6 individual runs.

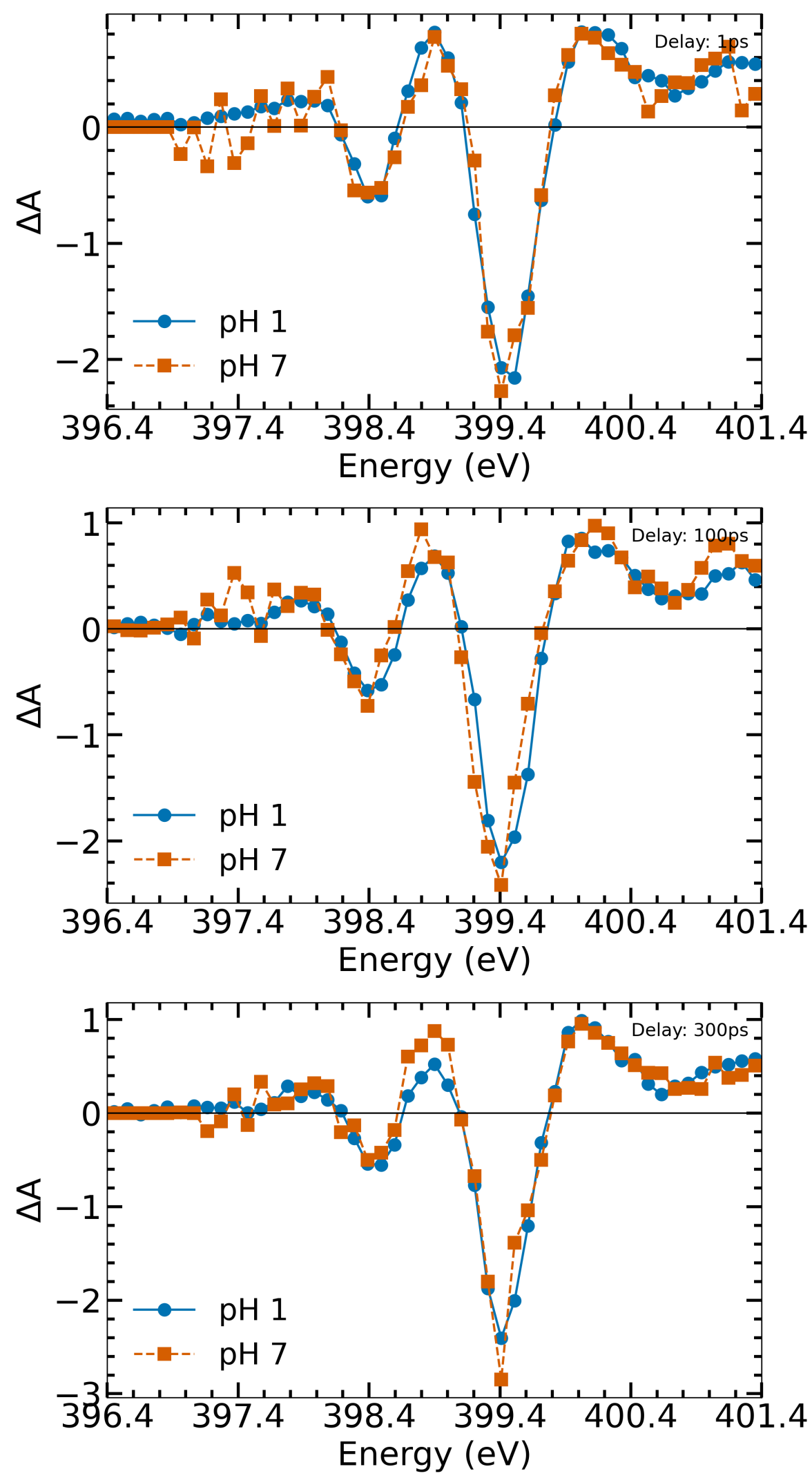

Supplementary Figure 8: Nitrogen K-edge transient-absorption difference spectra (ΔA) of $[Ru(bpy)_2(bpz)]^{2+}$ under neutral and acidic conditions at 1, 100, and 300 ps following 400 nm excitation. The spectra have been normalized to the area of the GSB between 399 and 400 eV.

3.2 Reconstruction of the $^3MLCT_{bpz}$ N K-edge Spectrum

The measured absorbance following photoexcitation of the sample contains a mixture of contributions from the ground and excited-state,

$$A_{\text{on}} = (1-\alpha)\, A_{\text{GS}} + \alpha\, A_{ES},$$

where α is the excited-state fraction, so that the pump–probe difference spectrum is

$$\Delta A = A_{\text{on}} - A_{\text{off}} = \alpha \big[A_{ES} - A_{\text{GS}} \big].$$

Solving for the excited-state absorbance yields

$$A_{ES} = \frac{\Delta A}{\alpha} + A_{\text{GS}}, \tag{1}$$

We use Eq. 1 to reconstruct the spectra of the $^3MLCT_{bpz}$ state using the data measured at 1 ps. Fig. 9 shows the result. The reconstructed spectra confirm a high-energy shift for both XAS peaks upon photoexcitation, as discussed in the main text.

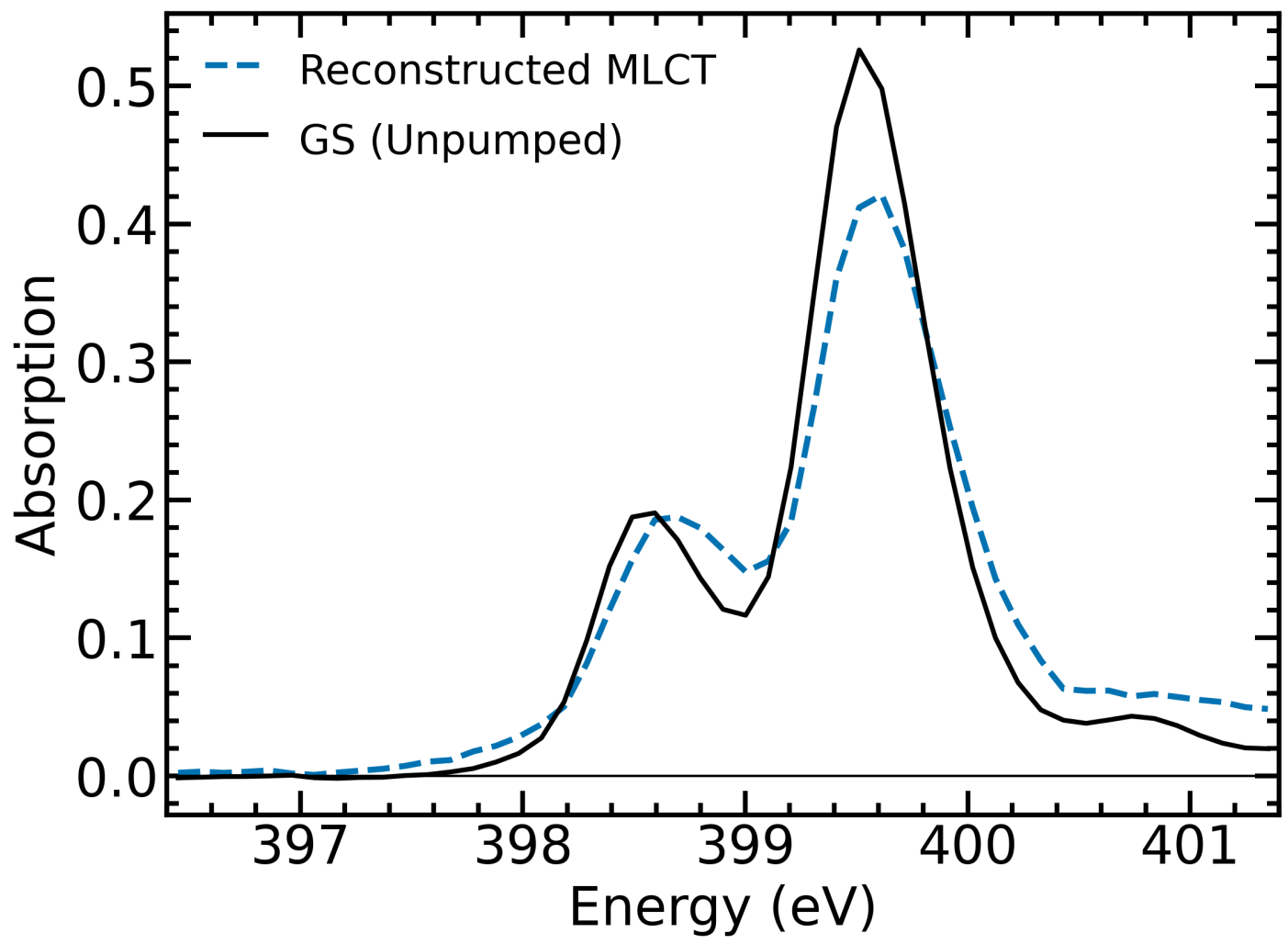

Supplementary Figure 9: Reconstructed $^3MLCT_{bpz}$ N K-edge absorption spectra derived from the static (black) and transient X-ray data measured at 1 ps delay upon photoexcitation of $[Ru(bpy)_2(bpz)]^{2+}$, using Eq. 1 with excitation fraction $\alpha = 0.5$.

4 Additional Data and Analysis: XSS data

4.1 Bulk solvent heating

The solvent–solvent contribution, which arises mainly from the changes in bulk solvent caused by the non-radiative relaxation of the photoexcited solute, was removed from the data as described in the following. At the timescales explored in our measurement (up to 1 ns, before thermal expansion), the main contribution arises from the increase in temperature of water and the resulting difference scattering signal can be described as [6]:

$$\Delta S_{heat}(Q,t) = \Delta T(t) \cdot \frac{\delta S(Q)}{\delta T}\Big|_{\rho} \tag{2}$$

where $\frac{\delta S(Q)}{\delta T}\Big|_{\rho}$ describes a change in temperature at constant density and is well-characterized for water [6], and $\Delta T(t)$ the increase in temperature at each pump-probe time delay t. We fit Supplementary Eq. 2 to the data at each time-delay and then subtract $\Delta S_{heat}(Q,t)$ from the data in order to subtract the contribution arising from bulk solvent heating. Suppl. Fig. 10 show raw scattering curves measured at 1 ps and 300 ps at both acidic and neutral condition and the result of the fit (black line). This subtraction doesn't affect the region below $Q < 1.5$ Å^{-1}, where we observe signatures of protonation. Supplementary Table 1 reports the optimized fit parameters obtained at each time delay. At 1 ps, the result for the neutral pH condition is 2.3 times higher than that for the acidic pH, consistent with the factor-of-two difference in laser power between the two conditions, as described in the Methods. Since the scattering signals measured at 1 ps are nominally identical for both conditions (Fig. 11), the excess heating observed under neutral pH is attributed to multi-photon excitation of the sample. However, the lowest excited-state populated in both cases remains the $^3MLCT_{bpz}$ (see next Section).

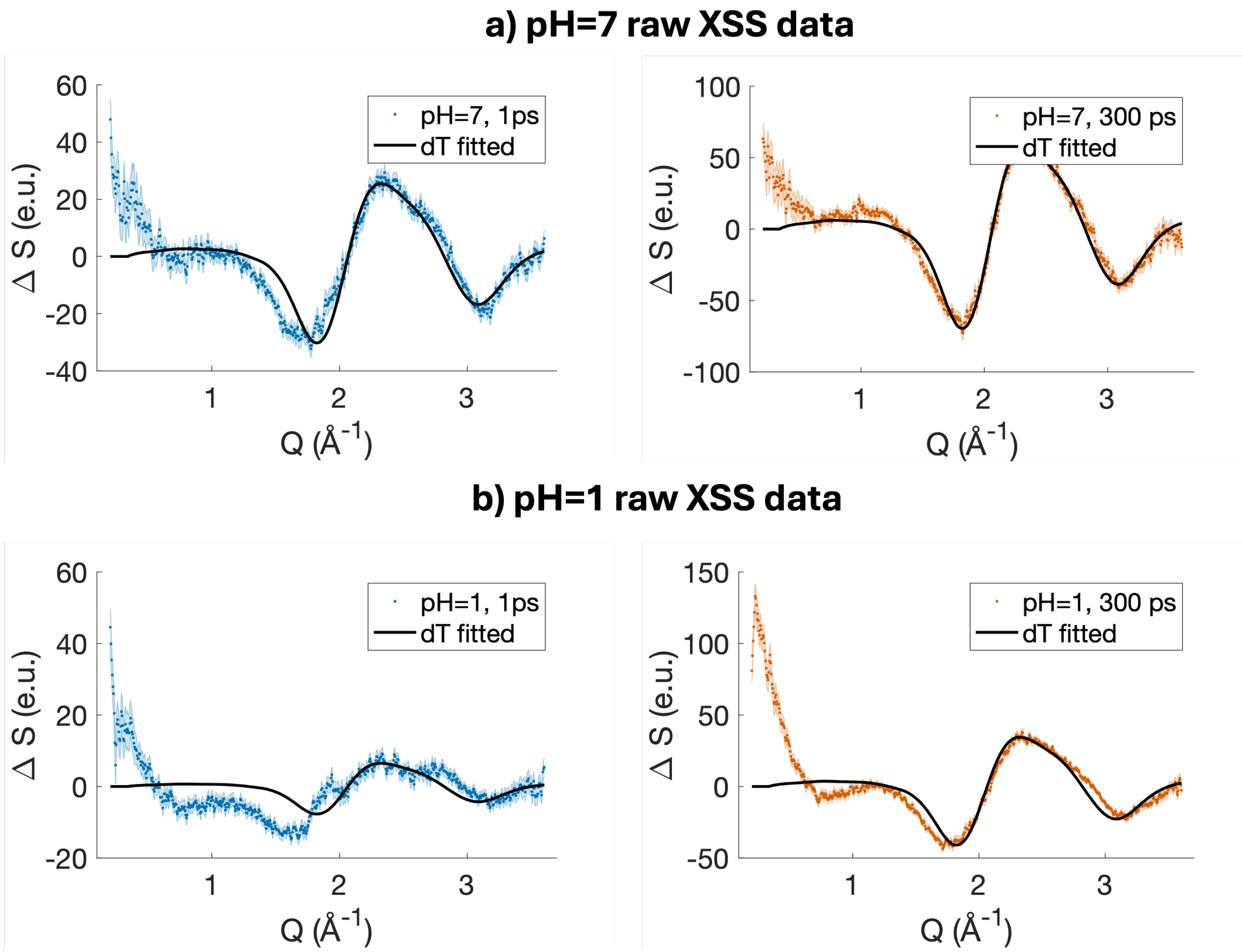

Supplementary Figure 10: Raw difference scattering signals measured at 1 ps (left) and 300 ps (right) after photoexcitation of $[Ru(bpy)_2(bpz)]^{2+}$ under neutral (top) and acidic (bottom) conditions. The black line represents a fit of Suppl. Eq. 2 to the data and corresponds to the contribution from bulk solvent heating following excited-state relaxation. This contribution is subtracted from the data presented in the main text. The subtraction has negligible impact on the signal below 1.5 Å^{-1}, which is the focus of our analysis. The uncertainty in S was obtained from the standard deviation of residuals to a locally fitted third-order polynomial within a 20-point Q-window around each Q point.

	1 ps (K)	100 ps (K)	300 ps (K)
pH7	0.080 ± 0.002	0.294 ± 0.003	0.295 ± 0.003
pH1	0.027 ± 0.002	0.133 ± 0.002	0.156 ± 0.003

Supplementary Table 1: Best fit parameters obtained for ΔT after fitting Suppl. Eq. 2 to the data. Uncertainties are the standard errors of the estimated coefficients from the fit.

4.2 Neutral pH XSS data

Suppl. Fig. 11 shows that: a) the difference scattering signals measured at 1 ps after photoexcitation of $[Ru(bpy)_2(bpz)]^{2+}$ in neutral and acidic conditions are nominally identical after removal of the heating contribution. This is as expected, since both reflect structural changes associated with the population of the $^3MLCT_{bpz}$ state; and b) there is no significant spectral evolution in the data measured under neutral conditions between 1 and 300 ps, in contrast to the 0.1 M HCl data presented in the main text (Figure 3a).

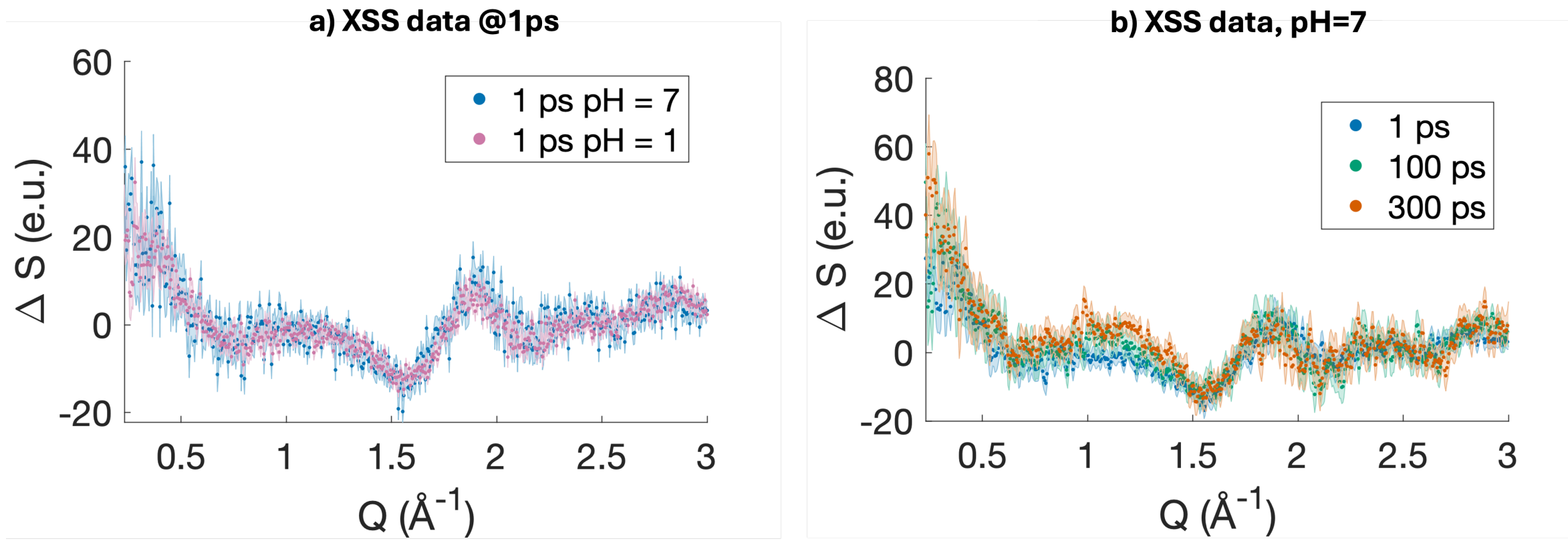

Supplementary Figure 11: a) Difference scattering signal measured at 1 ps after photoexcitation of $[Ru(bpy)_2(bpz)]^{2+}$ in neutral and acidic conditions. b) Difference scattering signal measured after photoexcitation of $[Ru(bpy)_2(bpz)]^{2+}$ in neutral conditions. The uncertainty in ΔS was obtained from the standard deviation of residuals to a locally fitted third-order polynomial within a 20-point Q-window around each Q point.

5 Transition-Specific TDDFT Analysis of Nitrogen K-Edge X-ray Absorption Spectra

Suppl. Fig. 12 shows the spectra of the 20 QM/MM snapshots and their weighted average, with weights constructed based on calculated total DFT energy, as described in the Methods.

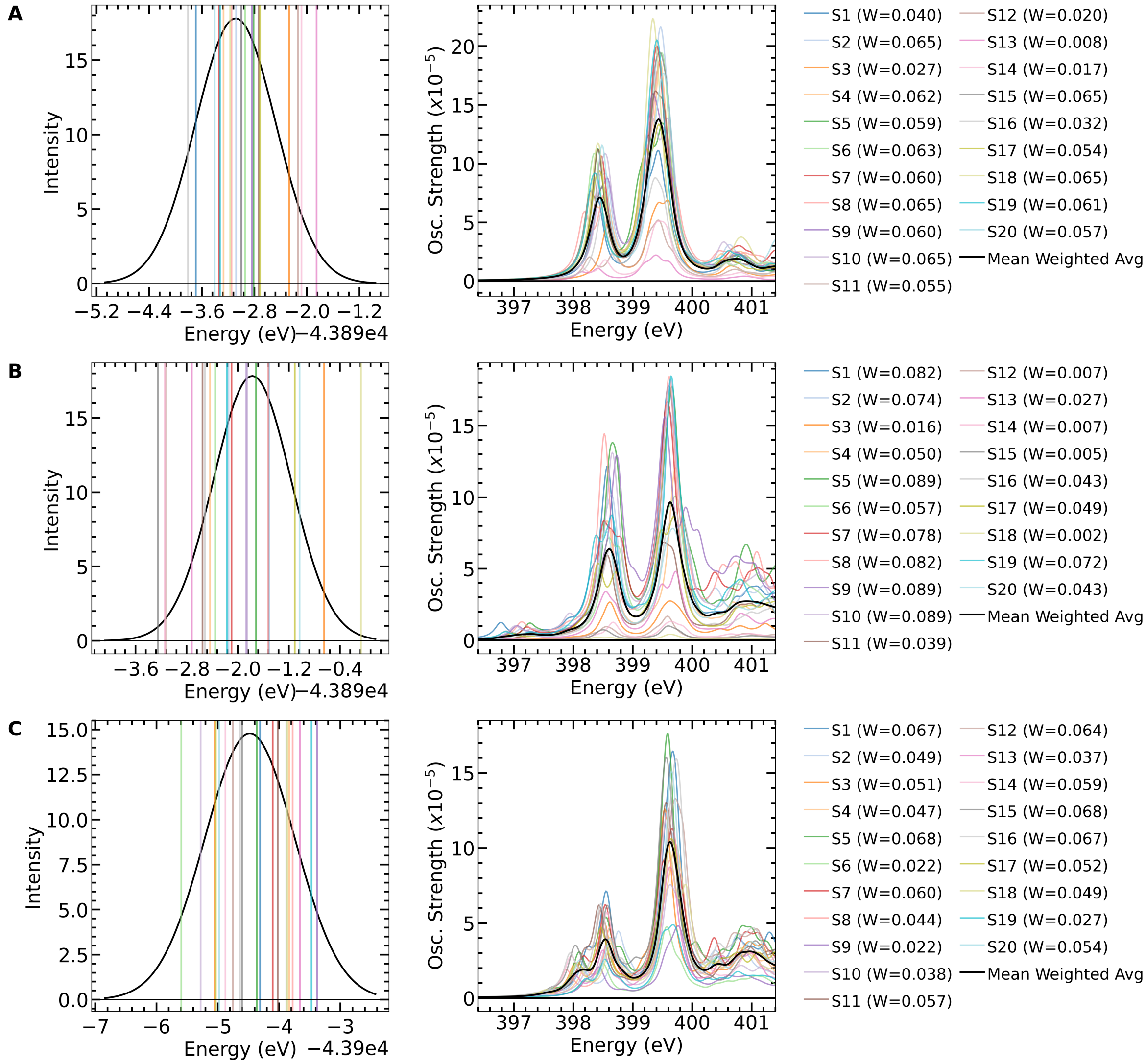

Supplementary Figure 12: Weight distributions and weighted TDDFT N K–edge spectra for the twenty QM/MM snapshots. Left-hand panels: The normalized Gaussian (black curve) fitted to the distribution of total DFT energies obtained from considering all snapshots in a 10 ps QM/MM trajectory. Colored vertical sticks are the Total DFT Energies of the 20 snapshots here considered. The color of each stick is reused in the corresponding right-hand panel. Right-hand panels: Interpolated, energy-shifted TDDFT absorption spectra for every snapshot (colored curves) multiplied by its statistical weight W taken from the left-hand panel. The thick black curve is the mean of all weighted spectra and represents the spectrum ultimately compared with the experiment. Rows correspond to the three electronic states analysed in this work: (A) ground-state S_0 (top row); (B) triplet MLCT state T_1 (middle row); (C) protonated triplet MLCT state p-T_1 (bottom row). The legends list every snapshot with its associated weight W; axes are identical within each column.

Suppl. Fig. 13 (panel B) shows the spectra of individual QM/MM snapshots used for the detailed analysis presented in Figure 3 of the main text. Specifically, these spectra correspond to Snapshot 1 in Suppl. Fig. 12. The transient signals shown in panel C, constructed from the spectra in panel B, are generally consistent with those obtained by averaging over all 20 snapshots. Below, we describe the character of the dominant electronic transitions and the key virtual orbitals responsible for the spectral features.

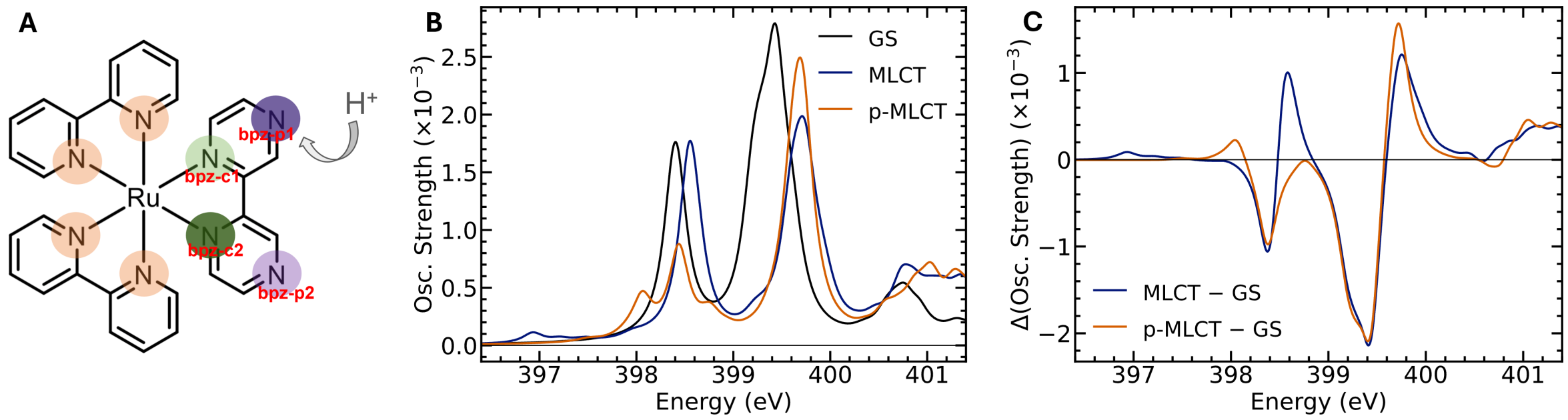

Supplementary Figure 13: (A) $[Ru(bpy)_2(bpz)]^{2+}$ complex with individual nitrogen sites labeled. The bipyrazine (bpz) nitrogens (green and purple) are the primary sites for MLCT and protonation. (B) Computed steady-state absorption spectra for one QM/MM snapshot (Snapshot 1 in Suppl. Fig. 12) of ground-state, $^3MLCT_{bpz}$, and $^3MLCT_{bpzH}$ states. (C) Corresponding Transient absorption difference spectra.

5.1 Orbitals Visualization and Analysis

Supplementary Table 2 shows relevant virtual valence orbitals for the ground-state (S_0), $^3MLCT_{bpz}$ (T_1) and protonated $^3MLCT_{bpzH}$ (p-T_1) states. In the GS, orbital 130 is the highest occupied molecular orbital (HOMO), and 131 the lowest unoccupied molecular orbital (LUMO), which is localized on the bpz ligand. LUMO+1 and LUMO+2 are localized on the bpy ligands. The T_1 and p-T_1 state are open-shell. The lowest energy vacancy is orbitals 130β, while a single electron occupies 131α. The second lowest energy vacancy (orbital 131β) is localized on the bpy ligand in the T_1 state, but on the bpz ligand in the p-T_1 state.

Ground State S0			^{3}MLCT$_{\mathbf{bpz}}$ (T$_1$)		^{3}MLCT$_{\mathbf{bpzH}}$ (p-T$_1$)	
**Orbital Number**	**Orbitals**	**Energy (eV)**	**Orbitals**	**Energy (eV)**	**Orbitals**	**Energy (eV)**
130	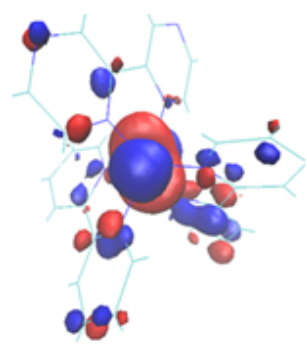	N/A	α 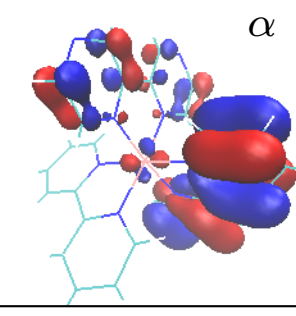	N/A	α 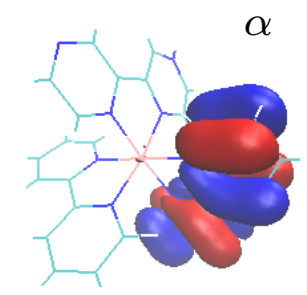	N/A
			β 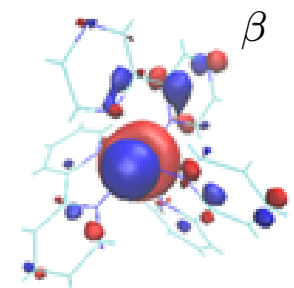	-6.72	β 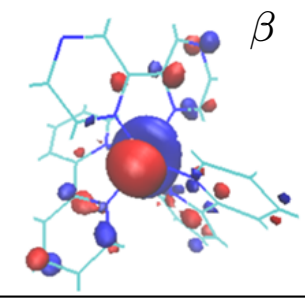	-7.81
131	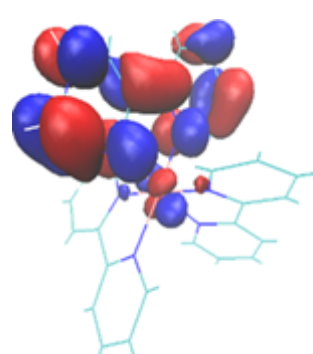	-5.01	α 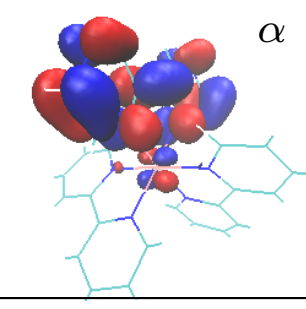	N/A	α 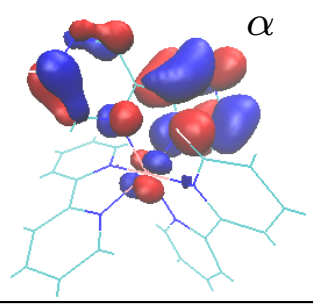	N/A
			β 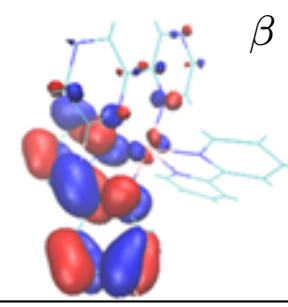	-4.81	β 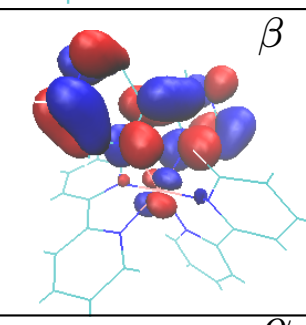	-6.1
132	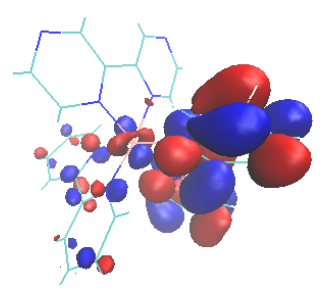	-4.797	α 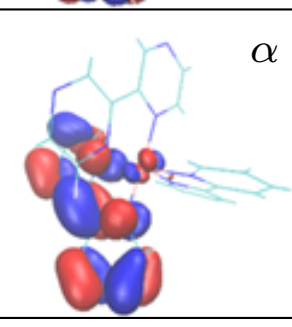	-4.81	α 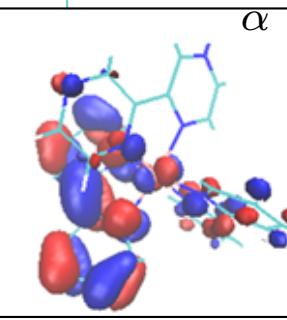	-5.73
			β 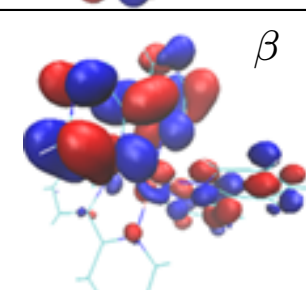	-4.66	β 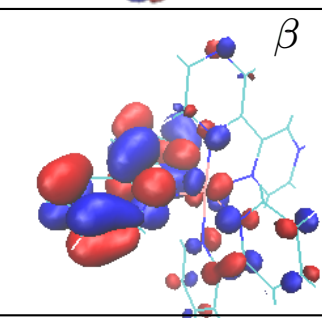	-5.63
133	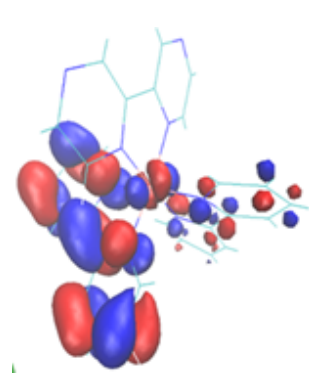	-4.199	α 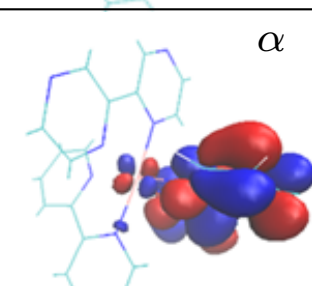	-4.64	α 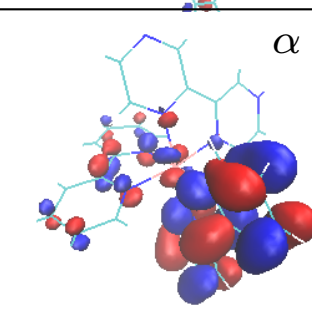	-5.65
			β 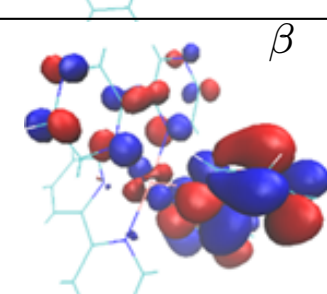	-4.61	β 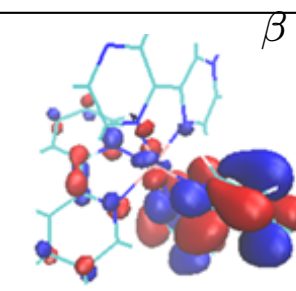	-5.53
134	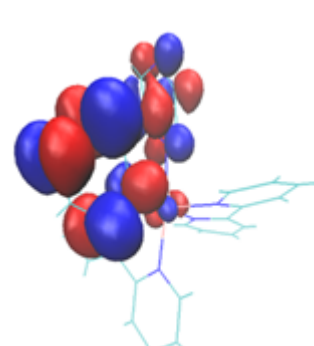	-3.997	α 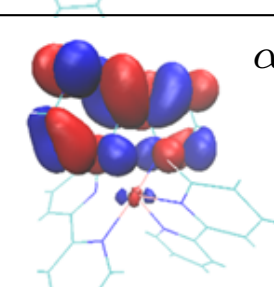	-4.09	α 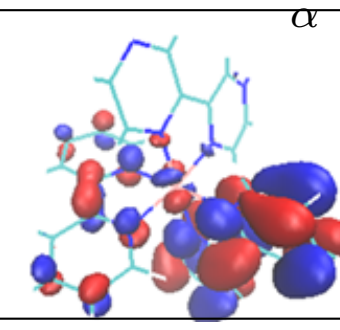	-5.55
			β 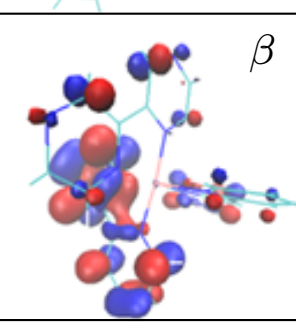	-3.87	β 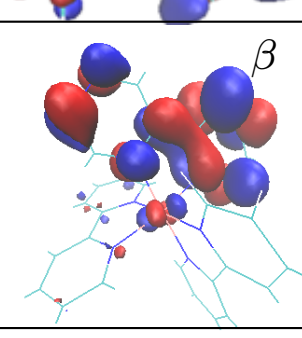	-5.37

Supplementary Table 2: Comparison of selected valence virtual orbitals (indices 130–134) in the ground-state S_0, the ^{3}MLCT$_{\mathrm{bpz}}$ (T$_1$), and the ^{3}MLCT$_{\mathrm{bpzH}}$ state (p-T$_1$). Energies are given in eV; N/A indicates that the orbital is occupied in the corresponding spin manifold. For the open–shell states both α and β channels are shown. Isosurfaces are plotted at ± 0.03 a.u. (red/blue for opposite phases) by using Visual Molecular Dynamics (VMD)[7].

Supplementary Tables 3-7 quantify how the eight lowest virtual vectors (indices 130–137) are built from the atomic basis of $[Ru(bpy)_2(bpz)]^{2+}$ in its three electronic states. For each virtual orbital, only the first 10 most dominant atomic contributions are considered and summed over atoms belonging to carbon atoms, ruthenium atom and six chemically distinct nitrogen groups (bpy, bpz-c1, bpz-c2, bpz-p1, bpz-p2, as shown in Suppl. Fig. 13. Finally, the energy of the core-level orbitals (N 1s) in the ground and MLCT states are shown in Suppl. Table 8. We note that the energies of the protonated MLCT orbitals cannot be directly compared with those of the GS and MLCT states because of the difference in the total charge of the complex.

Vector	C-total	N-bpy-total	N-bpz-c1	N-bpz-c2	N-bpz-p1	N-bpz-p2	Ru
131	15.4	0.0	21.5	38.5	0.0	0.0	0.0
132	33.4	44.7	0.0	0.0	0.0	0.0	0.0
133	70.6	29.7	0.0	0.0	0.0	0.0	0.0
134	53.6	0.0	8.5	0.0	0.0	38.0	0.0
135	80.3	0.0	0.0	0.0	7.6	12.1	0.0
136	90.7	9.3	0.0	0.0	0.0	0.0	0.0
137	84.3	0.0	0.0	0.0	15.7	0.0	0.0

Supplementary Table 3: Percent contributions from carbon, nitrogen, and ruthenium atoms to the TDDFT-calculated vectors 130–137 of the ground-state of $[Ru(bpy)_2(bpz)]^{2+}$, relative to the sum of the ten most dominant contributions for each vector. Only atoms contributing $\geq 1\%$ are included. Nitrogen atoms are classified according to the scheme shown in Suppl. Fig. 13(A).

Vector	C-total	N-bpy	N-bpz-c1	N-bpz-c2	N-bpz-p1	N-bpz-p2	Ru
131	16.5	0.0	44.8	38.7	0.0	0.0	0.0
132	37.6	39.6	22.8	0.0	0.0	0.0	0.0
133	54.1	45.9	0.0	0.0	0.0	0.0	0.0
134	0.0	0.0	16.5	17.5	40.1	25.9	0.0
135	94.2	5.5	0.0	0.0	0.0	0.0	0.0
136	7.7	36.9	0.0	0.0	0.0	0.0	55.4
137	99.1	10.9	0.0	0.0	0.0	0.0	0.0

Supplementary Table 4: Percent contributions from carbon, nitrogen, and ruthenium atoms to the TDDFT-calculated α vectors 130–137 of the $^3MLCT_{bpz}$ state of $[Ru(bpy)_2(bpz)]^{2+}$, relative to the sum of the ten most dominant contributions for each vector. Only atoms contributing $\geq 1\%$ are included. Nitrogen atoms are classified according to the scheme shown in Suppl. Fig. 13(A).

Vector	C-total	N-bpy	N-bpz-c1	N-bpz-c2	N-bpz-p2	Ru
130	2.9	0.0	0.0	0.0	0.0	97.1
131	56.0	40.2	22.0	0.0	0.0	0.0
132	16.0	0.0	39.6	44.4	0.0	0.0
133	54.0	46.0	0.0	0.0	0.0	0.0
134	99.9	0.0	0.0	0.0	0.0	0.0
135	100.0	9.3	0.0	0.0	0.0	0.0
136	92.2	0.0	7.7	0.0	0.0	0.0
137	62.3	0.0	15.1	22.5	0.0	0.0

Supplementary Table 5: Percent contributions from carbon, nitrogen, and ruthenium atoms to the TDDFT-calculated β vectors 130–137 of the ^{3}MLCT$_{\text{bpz}}$ state of $[\text{Ru(bpy)}_2\text{(bpz)}]^{2+}$, relative to the sum of the ten most dominant contributions for each vector. Only atoms contributing $\geq$ 1% are included. Nitrogen atoms are classified according to the scheme shown in Suppl. Fig. 13(A).

Vector	C-total	N-bpy	N-bpz-c1	N-bpz-c2	N-bpz-p1	N-bpz-p2	Ru
131	12.6	0.0	48.5	0.0	38.9	0.0	0.0
132	59.3	0.0	0.0	0.0	40.7	0.0	0.0
133	27.6	48.3	0.0	0.0	24.1	0.0	0.0
134	63.1	0.0	23.1	22.7	0.0	0.0	0.0
135	36.3	0.0	0.0	0.0	0.0	16.0	47.7
136	37.9	43.6	0.0	0.0	9.4	9.2	0.0
137	80.3	0.0	0.0	0.0	7.4	0.0	12.4

Supplementary Table 6: Percent contributions from carbon, nitrogen, and ruthenium atoms to the TDDFT-calculated α vectors 130–137 of the ^{3}MLCT$_{\text{bpzH}}$ state of $[\text{Ru(bpy)}_2\text{(bpz)}]^{2+}$, relative to the sum of the ten most dominant contributions for each vector. Only atoms contributing $\geq$ 1% are included. Nitrogen atoms are classified according to the scheme shown in Suppl. Fig. 13(A).

Vector	C-total	N-bpy	N-bpz-c1	N-bpz-c2	N-bpz-p1	N-bpz-p2	Ru
130	1.1	0.0	0.0	0.0	0.0	0.0	98.9
131	16.1	0.0	50.1	16.8	17.0	0.0	0.0
132	35.7	48.6	0.0	0.0	0.0	0.0	0.0
133	53.5	0.0	23.1	23.5	0.0	0.0	0.0
134	76.0	0.0	0.0	0.0	24.0	0.0	0.0
135	36.1	0.0	0.0	0.0	0.0	15.3	48.7
136	47.7	52.3	0.0	0.0	0.0	0.0	0.0
137	93.3	0.0	0.0	0.0	6.7	0.0	0.0

Supplementary Table 7: Percent contributions from carbon, nitrogen, and ruthenium atoms to the TDDFT-calculated β vectors 130–137 of the ^{3}MLCT$_{\text{bpzH}}$ state of $[\text{Ru(bpy)}_2\text{(bpz)}]^{2+}$, relative to the sum of the ten most dominant contributions for each vector. Only atoms contributing $\geq$ 1% are included. Nitrogen atoms are classified according to the scheme shown in Suppl. Fig. 13(A).

Supplementary Table 8: 1s core-levels energies (in eV) for individual nitrogen atoms in different states.

N type	S_0	T_1a	T_1b	T_1a-S_0	T_1b-S_0	T_1(avg.)-S_0
bpz-p1	-393.31	-392.79	-392.73	0.52	0.58	0.55
bpz-p2	-393.16	-392.88	-392.85	0.28	0.31	0.30
bpz-c2	-394.29	-393.88	-393.74	0.41	0.55	0.48
bpz-c1	-394.28	-393.86	-393.73	0.42	0.55	0.49
bpy (ave.)	-393.65	-394.38	-394.39	-0.73	-0.74	-0.74

5.2 Site–resolved N K-edge Excitations

Supplementary Tables 9-11 collect the dominant core–to–virtual excitations that build the calculated N K-edge spectra of $[Ru(bpy)_2(bpz)]^{2+/3+}$ in the three electronic states and shown in Figure 2g-h of the main text. In TDDFT each excited-state is an eigenstate of the Casida equation and is expressed as a linear combination of single $1s \rightarrow$ virtual promotions; the fractional coefficients shown below are the expansion weights of those eigenstates. For a selected TDDFT root in the 396.4–401.4 eV window we list the dominant single electron transitions: (i) the nitrogen atom involved, (ii) the 1s orbital energy, (iii) the leading unoccupied vectors with their orbital energies, (iv) the total oscillator strength, and (v) the squared coefficient representing the weight of the single electron transition. Rows are color-coded (violet = bpz-p, green = bpz-c, orange = bpy) as in Figure 2 of the main text. Suppl. Fig. 14 illustrates the dominant transitions for the bpz Ns into an energy-level scheme. Specifically, arrows represent 1s to unoccupied orbital transitions that contributes at least 10 % to a root. Arrow thickness is proportional to the squared coefficient.

Roots (eV)	N	Occ. MO En. (eV)	Unocc. MO Vec. #	Unocc. MO En. (eV)	Tot. osc. str.	Coef. (%)
398.37	bpz_p2	-393.16	131	-5.01	0.042	62.4
			134	-4.00		31.4
398.43	bpz_p1	-393.31	131	-5.01	0.042	70.6
			134	-4.00		11.6
			135	-3.89		11.6
399.18	bpz_c1	-394.29	131	-5.01	0.035	79.2
			135	-3.89		9.6
			134	-4.00		7.3
399.27	bpz_c2	-394.28	131	-5.01	0.033	79.2
			134	-4.00		17.6
399.34	bpz_p2	-393.16	134	-4.00	0.007	59.3
			131	-5.01		29.2
399.47 (ave.)	bpy	-393.65	132, 133	-4.50	0.028	70.6
	bpy		131, 132, 133, 136, 137, 138, 139	-3.84	0.028	9.1
399.48	bpz_p1	-393.31	135	-3.89	0.010	72.3
			134	-4.00		19.4

Supplementary Table 9: Dominant ground-state N K-edge excitations of $[Ru(bpy)_2(bpz)]^{2+}$ obtained from TDDFT. For each root the table lists the nitrogen site, the energy of the occupied 1s orbital, the main unoccupied vectors and their orbital energies, the total oscillator strength, and the squared coefficient of the leading single-electron transitions. Rows are color-coded to highlight the three classes of nitrogens: peripheral bpz (violet), coordinated bpz (green), and bpy (orange).

**Roots (eV)**	**N**	α **Occ. MO En. (eV)**	α **Unocc. MO Vec. #**	α **Unocc. MO En. (eV)**	β **Occ. MO En. (eV)**	β **Uncc. MO (Vec.) #**	β **Unocc. MO En. (eV)**	**Tot. osc. Str.**	**Coef. (%)**
396.93	bpz_p1	_	_	_	-392.73	130	-6.72	0.0046	86.5
397.21	bpz_p2	_	_	_	-392.84	130	-6.72	0.0016	92.2
397.47	bpz_c2	_	_	_	-393.72	130	-6.72	0.0011	98.0
397.93	bpz_p2	_	_	_	-392.84	132	-4.66	0.0013	27.0
		_	_	_		137	-3.55		20.3
		-392.87	134	-4.1		_	_		19.4
397.98	bpz_p1	-392.78	134	-4.1	_	_	_	0.0008	20.3
		_	_	_	-392.73	132	-4.7		24.6
		_	_	_		130	-6.72		11.6
398.52	bpz_p1	-392.78	134	-4.1		_	_	0.0320	51.8
		_	_	_	-392.73	132	-4.66		17.6
398.55	bpz_p2	-392.87	134	-4.1		_	_	0.0380	46.2
		_	_	_	-392.84	132	-4.66		18.5
398.57	bpz_c2	_	_	_	-393.73	132	-4.66	0.0128	56.3
		_	_	_		137	-3.55		13.7
		_	_	_		133	-4.61		11.6
398.63	bpz_c1	_	_	_	-393.744	132	-4.66	0.0134	54.8
		_	_	_		133	-4.61		12.25
		_	_	_		137	-3.55		7.8
399.20	bpz_p1	_	_	_	-392.73	132	-4.66	0.0046	28.09
		_	_	_		136	-3.55		22.1
		_	_	_		138	-3.47		12.3
399.28	bpz_p2	_	_	_	-392.84	131	-4.81	0.0031	54.8
		_	_	_		137	-3.55		26.0
399.38		_	_	_		131	-4.81	0.0018	39.7
		_	_	_		132	-4.66		27.0
		_	_	_		137	-3.55		19.4
399.67	bpy (ave.)	-394.37	132, 133	-4.72	_	_	_	0.030	39.1
399.67	bpy (ave.)	_	_	_	-394.36	131, 133	-4.71	0.030	37.9
399.85	bpz_c2	-393.85	134	-4.1	_	_	_	0.012	72.3
399.95	bpz_c1	-393.74	134	-4.1	_	_	_	-0.011	81

Supplementary Table 10: Dominant N K-edge excitations of the triplet MLCT (T_1) state of $[Ru(bpy)_2(bpz)]^{2+}$ extracted from TDDFT. Columns list the nitrogen site, the occupied 1s orbital energy, the principal unoccupied vectors and their energies, the total oscillator strength, and the squared coefficient of the leading single-electron transitions. Color shading matches Fig. S12: peripheral bpz nitrogens (violet), coordinated bpz nitrogens (green), and bpy nitrogens (orange).

**Roots (eV)**	**N**	α **Occ. MO En. (eV)**	α **Unocc. MO Vec. #**	α **Unocc. MO En. (eV)**	β **Occ. MO En. (eV)**	β **Uncc. MO (Vec.) #**	β **Unocc. MO En. (eV)**	**Tot. osc. Str.**	**Coef. (%)**
398.06	bpz_c1	_	_	_	-394.80	131	-6.09	0.019	79.2
398.43	bpz_p2	-394.24	132	-5.73	_	_	_	0.042	30.3
		_	_	_	-394.25	131	-6.09		30.3
398.75	bpz_p1	_	_	_	-395.72	130	-7.81	0.010	70.6
		_	_	_		131	-6.09		15.4
		_	_	_		134	-5.37		10.8
398.92	bpz_c2	_	_	_	-395.54	131	-6.09	0.002	43.6
		-395.57	132	-5.73	_	_	_	_	19.4
399.38	bpz_p2	_	_	_	-394.25	135	-4.92	0.003	49.0
		_	_	_		131	-6.09		32.5
399.56	bpz_p1	_	_	_	-395.72	131	-6.09	0.009	44.9
		_	_	_		130	-7.81		28.1
		_	_	_		134	-5.37		21.2
399.6	bpz_c2	-395.6	132	-5.73	_	_	_	0.031	57.8
399.6		_	_	_	-395.54	131	-6.09		20.8
399.7	bpy (ave.)	-395.34	133, 134	-5.60	_	_	_	0.029	30.0
399.7	bpy (ave.)	_	_	_	-395.27	132, 133	-5.58	0.029	37.8

Supplementary Table 11: Dominant N K-edge excitations of the triplet protonated MLCT (p-T_1) state of $[Ru(bpy)_2(bpz)]^{2+}$ extracted from TDDFT. Columns list the nitrogen site, the occupied 1s orbital energy, the principal unoccupied vectors and their energies, the total oscillator strength, and the squared coefficient of the leading single-electron transitions. Color shading matches Fig. S12: peripheral bpz nitrogens (violet), coordinated bpz nitrogens (green), and bpy nitrogens (orange).

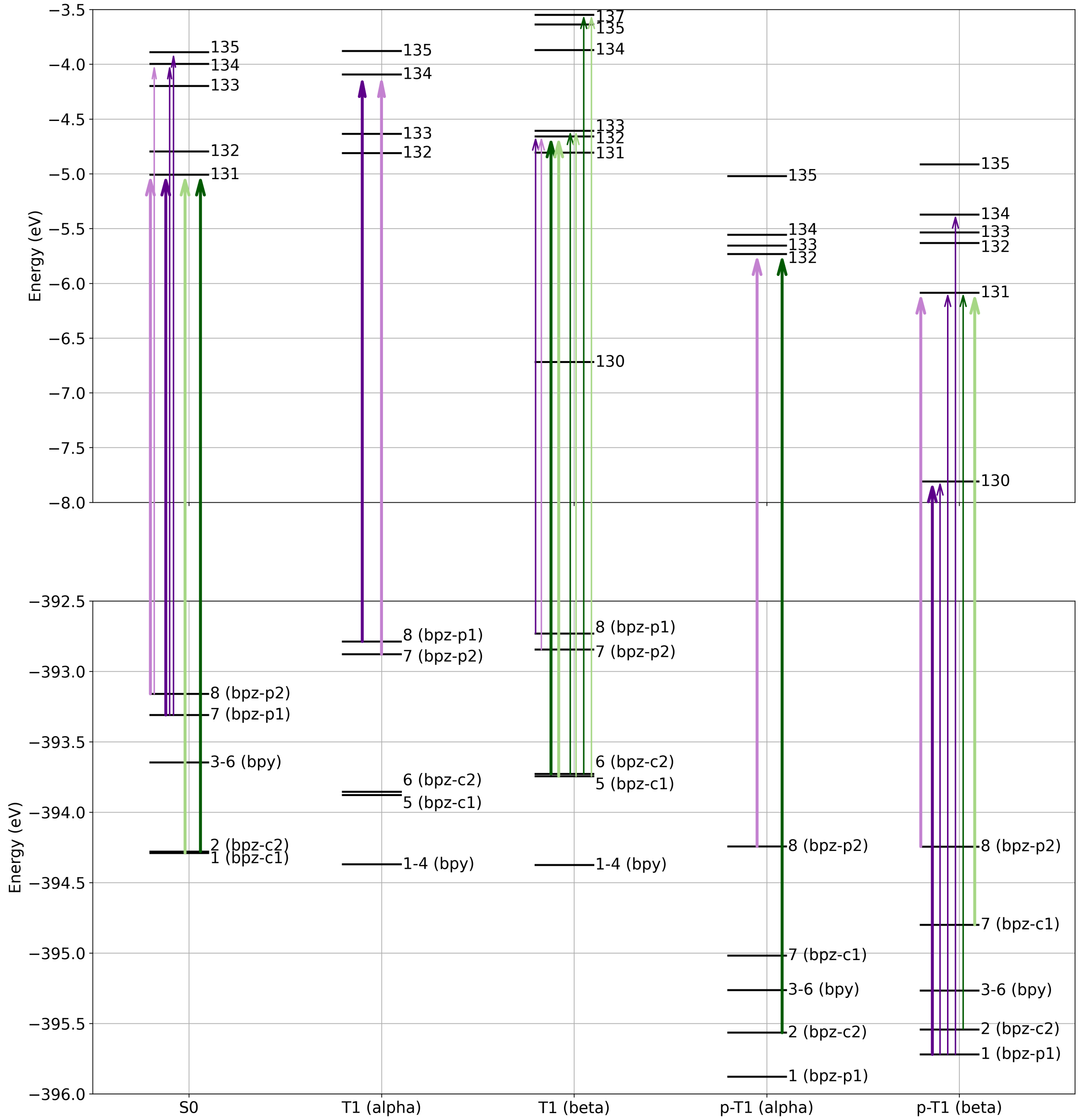

Supplementary Figure 14: One–electron picture of the dominant N 1s → virtual transitions that build up the N K-edge spectra of Figure 2. Horizontal black ticks mark orbital energies for a representative QM/MM snapshot of each electronic state. The lower ticks correspond to the occupied N 1s levels; the upper ticks to the unoccupied levels. Colored arrows show single-electron transitions that contribute at least 10 % to the TDDFT roots with highest oscillators strength. Violet arrows originate from peripheral bpz nitrogens, green arrows from Ru-coordinated bpz nitrogens as shown in Suppl. Fig. 13. Arrow thickness is proportional to the squared TDDFT configuration coefficient, so thicker arrows represent the largest single -configuration weights in the eigenvectors (see Supplementary Tables 9-11).

6 Additional structural insights from MD simulations

6.1 Intramolecular Structural Changes

Supplementary Tables 12, 13, and 14 report the structure and charges of, respectively, the ^{1}GS, ^{3}MLCT$_{\mathrm{bpz}}$ and ^{3}MLCT$_{\mathrm{bpzH}}$ states of $[\mathrm{Ru(bpy)_2(bpz)}]^{2+/3+}$ that are obtained from optimized QM/MM structures and used in the classical MD simulation. In the ground-state, the average Ru-N bond length is 2.094 Å with a standard deviation of 0.004 Å, indicating a symmetric environment around the Ru.

This symmetry is broken in the ^{3}MLCT$_{bpz}$ state, with the Ru-N bond length distances of the bpy ligand slightly elongated with respect to the ground-state (on average 2.103 Å), while the Ru-N bond length distances of the bpz ligand are slightly shorter than in the ground-state (on average 2.065 Å). In the protonated MLCT states, the symmetry is reinstalled with Ru-N bond length distances of 2.103 Å and 2.097 Å in the bpy and bpz ligands respectively. Overall, the average distance change in the ^{3}MLCT$_{bpz}$ and ^{3}MLCT$_{bpzH}$ with respect to the ground-state is of -0.004 and 0.008 Å, making intramolecular structural changes negligible for our purposes. Suppl. Fig. 15 shows the difference scattering signal arising from intramolecular (solute–solute) and intermolecular (solute–solvent) changes in the MLCT and protonated MLCT states.

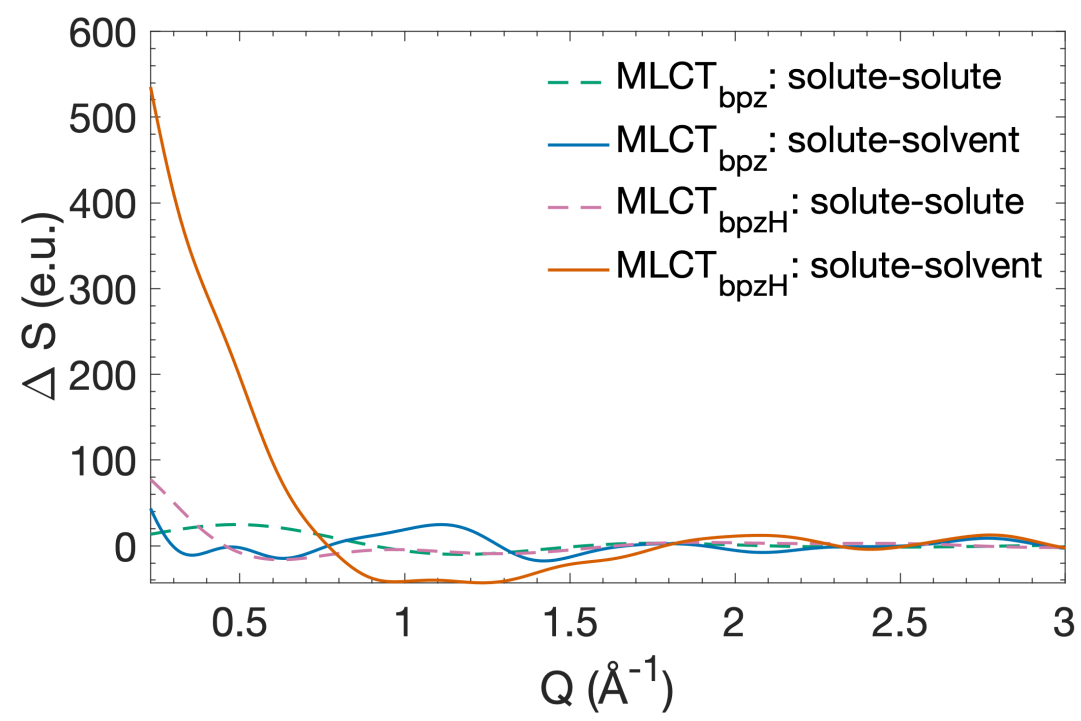

Supplementary Figure 15: Difference scattering signal predicted by MD simulations arising from the changes in intra-molecular (solute–solute) and inter-molecular (solute–solvent) structure in the ^{3}MLCT$_{bpz}$ and ^{3}MLCT$_{bpzH}$ states. In the protonated state, the intra-molecular contribution is negligible compared to the pronounced changes in the solvation shell structure.

6.2 Individual solute–solvent contributions

Suppl. Fig. 16 shows the contributions of individual solute atom groups to the total solute–solvent scattering signal for the ^{3}MLCT$_{bpzH}$ state. The magnitude of each contribution depends on the scattering power of the element, its abundance in the complex, and the extent of the structural change. In the low-Q region of interest (0.2 Å $<$ Q $<$ 0.6 Å), where we observe signature of solvent-shell reorganization upon protonation , more than half of the signal arise from local changes around the bpz ligands, i.e. from the C$_{bpz}$ and N$_{bpz}$ contributions, as well as the corresponding contribution from the Ru. The latter cannot be disentangled along the three directions using the RDFs. Same consideration is valid for the ^{3}MLCT$_{bpz}$ state (Suppl. Fig. 17).

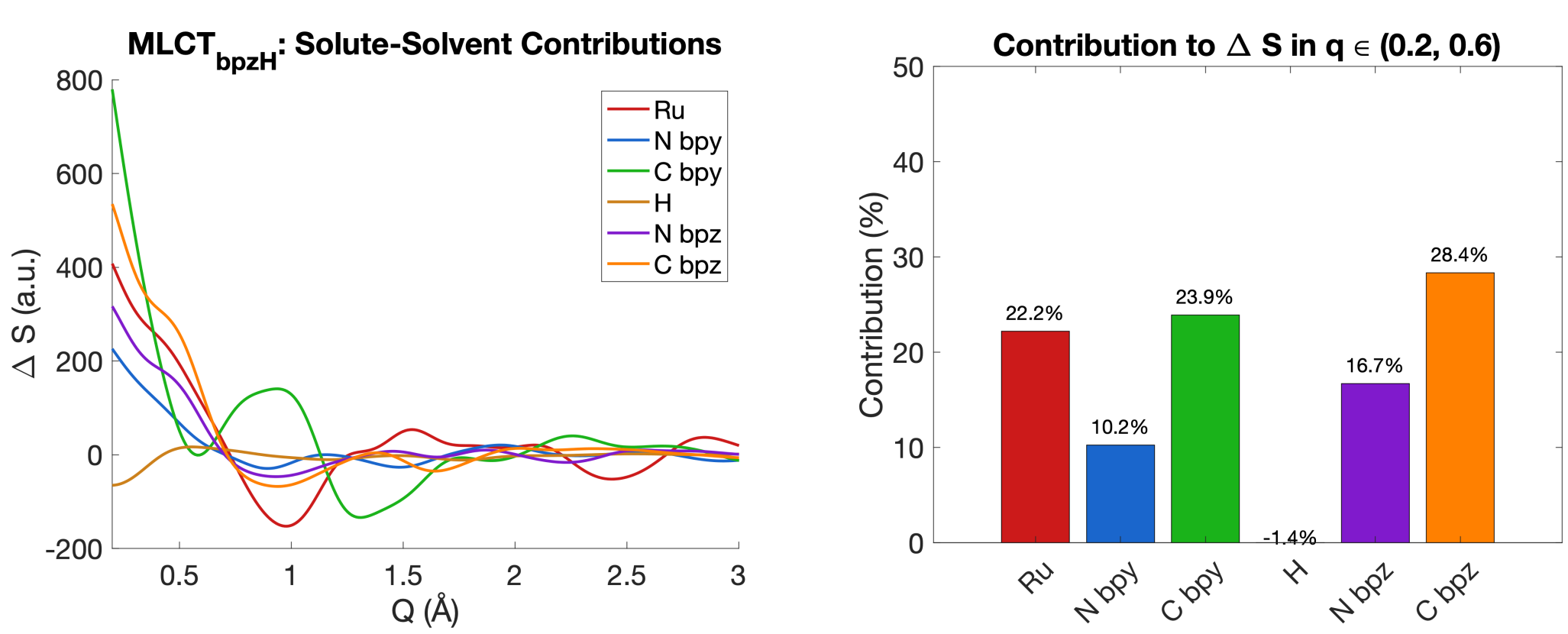

Supplementary Figure 16: (left) ^{3}MLCT$_{bpzH}$ difference scattering signals arising from changes in X-H$_w$ and X-O$_w$ pair distances, where X denotes each individual solute atom group. (right) % contribution to the total solute–solvent difference scattering signal in the (0.2 Å $<$ Q $<$ 0.6 Å) region.

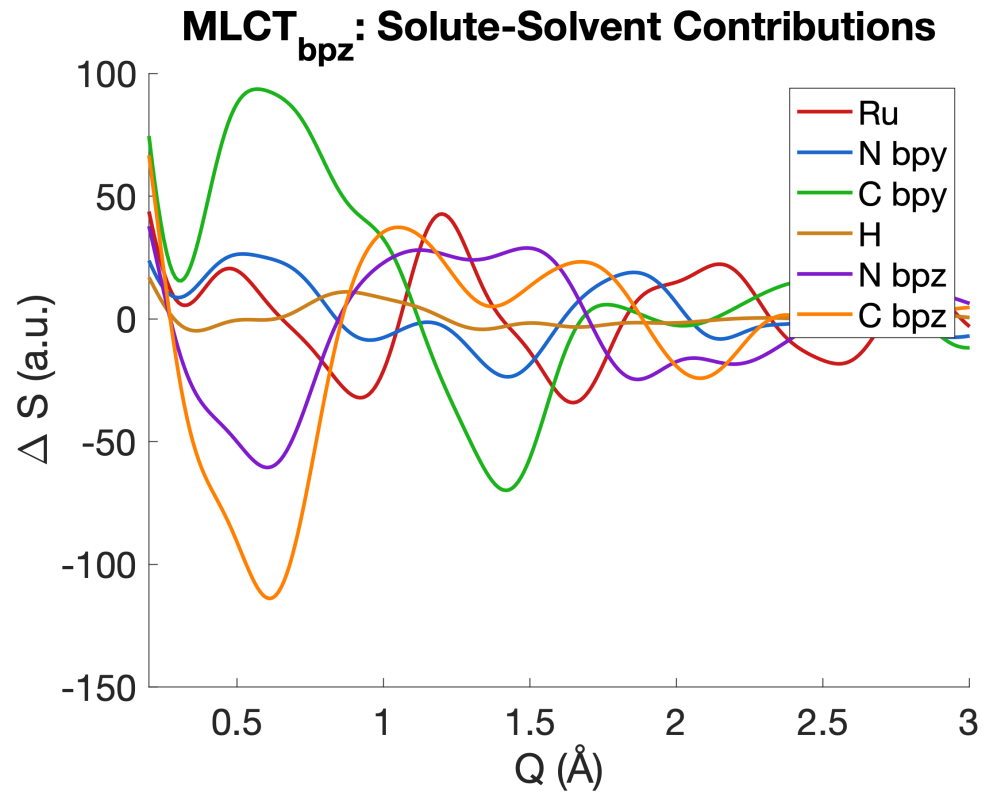

Supplementary Figure 17: (left) ^{3}MLCT$_{\mathrm{bpz}}$ difference scattering signals arising from changes in X-H$_{\mathrm{w}}$ and X-O$_{\mathrm{w}}$ pair distances, where X denotes each individual solute atom group.

Suppl. Fig. 18 shows solute–solvent RDFs calculated for selected groups of solute atoms, aimed at disentangling changes around the bpy and bpz ligands.

In the ^{3}MLCT$_{\mathrm{bpz}}$ state, the MD simulations predict strengthened hydrogen bonding between water molecules and the peripheral nitrogens of the bpz ligand (see N$_{\mathrm{bpz}}$ RDFs). At the same time, there is also a hint of hydrogen bonding between water and the carbons of the bpz ligand (see C$_{\mathrm{bpz}}$-H$_{\mathrm{w}}$ RDF), a feature previously reported in MD simulations of reduced bpy ligands [8]. However, while the hydrogens of water move closer to the carbons, the oxygens shift farther away, implying a reorientation of water molecules in the vicinity of the bpz ligand. This contrasts with Ref. [8], where both hydrogens and oxygens move closer.

In our MD simulations, the displacement of oxygen away from the carbons cancels the density accumulation around the peripheral bpz ligand, yielding a nominally zero low-Q solute–solvent signal. This result does not reproduce the experimental data, which show a small but positive increase. Given the limited amplitude of the ^{3}MLCT$_{\mathrm{bpz}}$ solute–solvent signal and the challenges in reproducing scattering contributions from MLCT populations, we defer a more detailed investigation of this discrepancy to future studies.

In the ^{3}MLCT$_{\mathrm{bpzH}}$ state, both the carbons and nitrogens of the bpz ligand experience increased electron density due to the approach of solvent oxygens. The majority of the solvent-shell reorganization is localized around the protonated pyrazine ring, as shown in Suppl. Fig. 19 and supported by changes in partial charges (Suppl. Tables 12–14). At the RDF level, it is difficult to disentangle solvation contributions around carbons from those around nitrogens because of their close spatial proximity. Furthermore, such distinctions would not be experimentally resolvable.

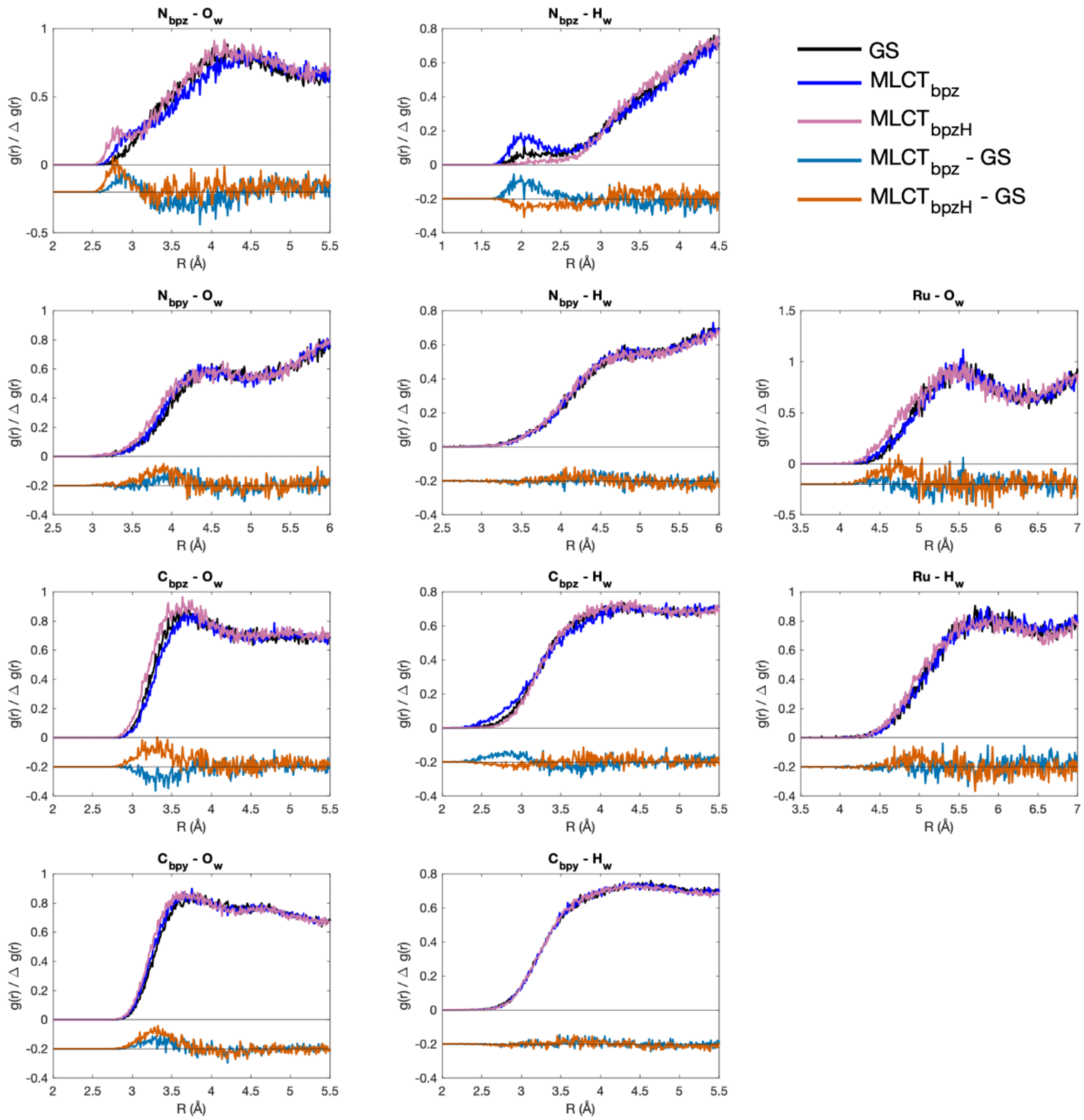

Supplementary Figure 18: Solute–solvent RDFs from the MD simulations in the three different electronic states of $[Ru(bpy)_2(bpz)]^{2+}$: ground-state (black), $^3MLCT_{bpz}$ (blue) and $^3MLCT_{bpzH}$ (pink). Light blue and orange lines represent difference RDFs (Δg(r)) between excited and ground states, offset by 0.2 for clarity.

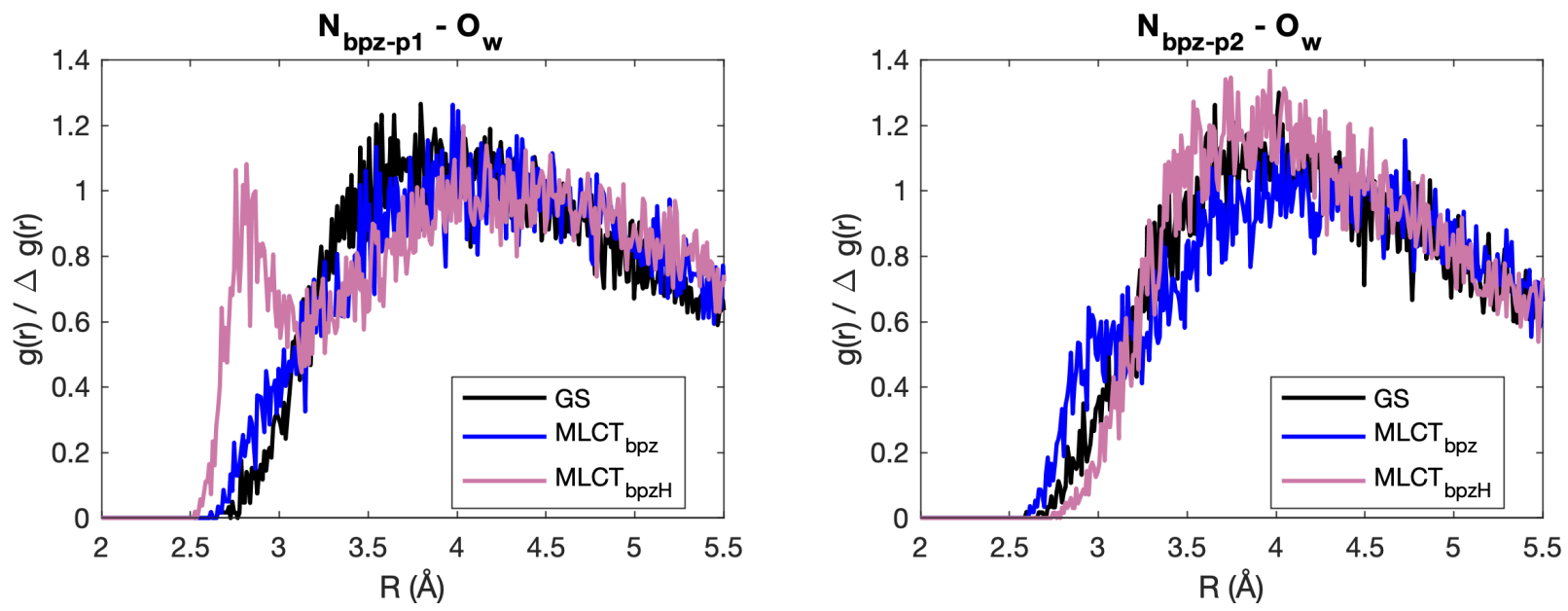

Supplementary Figure 19: RDFs between the peripheral nitrogens of the bpz ligand and the water oxygens in the three electric states. (Left/Right) N that is protonated/unprotonated in the $MLCT_{bpzH}$ state.

Supplementary Table 12: $[Ru(bpy)_2(bpz)]^{2+}$ S_0 structure and ESP partial charges used for the MD simulations.

Atom	Position	x	y	z	ESP
Ru	1	-0.665363	0.867954	-0.757584	-0.328879
N	2	0.582978	0.562566	-2.409811	0.039437
N	3	-0.305522	-1.189985	-0.643775	0.057323
C	4	0.953694	-0.732621	-2.635993	0.080358
C	5	1.071379	1.530741	-3.209994	-0.073182
C	6	1.949499	1.263751	-4.256815	-0.104341
C	7	2.325312	-0.056071	-4.499505	-0.003112
C	8	1.815530	-1.063419	-3.683843	-0.124870
C	9	0.428251	-1.714817	-1.669434	0.069317
C	10	-0.787701	-2.022655	0.302504	-0.091069
C	11	-0.580294	-3.397526	0.273745	-0.068867
C	12	0.148450	-3.943910	-0.783731	-0.059562
C	13	0.653677	-3.092053	-1.762397	-0.121868
H	14	0.770487	2.543535	-2.975675	0.165465
H	15	2.342053	2.082995	-4.848081	0.151324
H	16	3.028258	-0.303306	-5.287092	0.129569
H	17	2.117100	-2.089631	-3.851230	0.154133
H	18	-1.364838	-1.565484	1.096599	0.150464
H	19	-0.991673	-4.012514	1.067570	0.132049
H	20	0.320641	-5.013152	-0.851708	0.143291
H	21	1.204605	-3.497264	-2.601783	0.154140
N	22	0.894359	1.265368	0.587476	0.083501
N	23	-1.687532	0.999105	1.071824	0.029149
C	24	0.521495	1.454223	1.887590	0.093912
C	25	2.200348	1.363847	0.269164	-0.120623
C	26	3.181063	1.658477	1.209188	-0.082475
C	27	2.807118	1.880703	2.532625	0.035426
C	28	1.461280	1.777005	2.870025	-0.173830
C	29	-0.915543	1.280997	2.161927	0.089155
C	30	-3.008150	0.798238	1.252350	-0.046395
C	31	-3.613937	0.850059	2.501825	-0.095396
C	32	-2.830855	1.129451	3.620073	0.019415
C	33	-1.469486	1.352673	3.443621	-0.163421
H	34	2.454206	1.206815	-0.770601	0.179006
H	35	4.216756	1.716584	0.896408	0.115576
H	36	3.540464	2.139597	3.288174	0.124813
H	37	1.146273	1.955956	3.890189	0.157768
H	38	-3.582033	0.574803	0.360491	0.098703
H	39	-4.674771	0.665615	2.591019	0.156379
H	40	-3.268214	1.163635	4.611686	0.134287
H	41	-0.846457	1.565740	4.302939	0.161767
N	42	-1.155140	2.868766	-1.115022	0.310483
N	43	-2.310746	0.673811	-2.029336	0.309620
C	44	-2.248408	3.059413	-1.910229	-0.173494
C	45	-0.576482	3.966312	-0.594467	-0.353820
C	46	-1.096527	5.239810	-0.836001	0.454171
N	47	-2.179822	5.436269	-1.595432	-0.526464
C	48	-2.737943	4.351393	-2.132790	0.318597
C	49	-2.848896	1.846955	-2.464959	-0.098358
C	50	-2.848340	-0.462974	-2.516465	-0.370382
C	51	-3.902076	-0.433698	-3.426885	0.430127
N	52	-4.429271	0.713614	-3.873888	-0.524257
C	53	-3.901265	1.836939	-3.392861	0.322854
H	54	0.306331	3.825755	0.015032	0.205077
H	55	-0.633355	6.114611	-0.391854	0.057388
H	56	-3.615487	4.513478	-2.752400	0.080186
H	57	-2.429299	-1.399165	-2.169999	0.175269
H	58	-4.326433	-1.359210	-3.802968	0.051743
H	59	-4.320016	2.767969	-3.763409	0.083423

Supplementary Table 13: $[Ru(bpy)_2(bpz)]^{2+}$ T_1 structure and ESP partial charges used for the MD simulations.

Atom	Position	x	y	z	ESP
Ru	1	-0.071313	0.096102	-0.069956	0.041986
N	2	0.048618	0.070721	-0.239029	-0.039037
N	3	-0.023635	-0.109719	-0.059020	-0.044352
C	4	0.094697	-0.056209	-0.260560	0.057000
C	5	0.088445	0.170758	-0.319853	0.038574
C	6	0.178435	0.149929	-0.424055	-0.072304
C	7	0.226853	0.021265	-0.446342	-0.032086
C	8	0.183546	-0.083056	-0.364602	-0.112116
C	9	0.047714	-0.157820	-0.164839	0.137415
C	10	-0.067602	-0.195236	0.035502	-0.005139
C	11	-0.044700	-0.332225	0.028602	-0.135472
C	12	0.025830	-0.382849	-0.080746	0.015649
C	13	0.072517	-0.294771	-0.178063	-0.149690
H	14	0.049976	0.269282	-0.297080	0.123708
H	15	0.210861	0.234137	-0.483974	0.146882
H	16	0.299290	0.001707	-0.524541	0.150007
H	17	0.222312	-0.182961	-0.380205	0.161202
H	18	-0.123979	-0.152200	0.117416	0.120739
H	19	-0.082794	-0.396610	0.107164	0.155734
H	20	0.043943	-0.489411	-0.090833	0.131548
H	21	0.125307	-0.332454	-0.264782	0.157545
N	22	0.086193	0.136333	0.065397	0.003560
N	23	-0.172375	0.109221	0.112944	-0.016359
C	24	0.049559	0.146554	0.196361	0.095103
C	25	0.215414	0.153288	0.031321	-0.071407
C	26	0.313815	0.180600	0.125543	-0.059999
C	27	0.277798	0.190938	0.259765	0.015508
C	28	0.144277	0.173939	0.295224	-0.144496
C	29	-0.093978	0.127920	0.223165	0.097262
C	30	-0.305551	0.095644	0.128286	-0.004890
C	31	-0.366010	0.096246	0.253389	-0.085860
C	32	-0.286683	0.111783	0.366833	0.046277
C	33	-0.149448	0.128699	0.351177	-0.132338
H	34	0.239031	0.146803	-0.074098	0.175876
H	35	0.416183	0.194519	0.092948	0.115969
H	36	0.351662	0.213697	0.335808	0.137142
H	37	0.114280	0.183615	0.398794	0.158654
H	38	-0.362874	0.082625	0.037291	0.094593
H	39	-0.472998	0.084106	0.261428	0.159706
H	40	-0.330993	0.110719	0.465759	0.135159
H	41	-0.086422	0.141388	0.438179	0.146944
N	42	-0.127413	0.292320	-0.102266	0.286850
N	43	-0.235182	0.067810	-0.192079	0.297218
C	44	-0.238725	0.306207	-0.184865	-0.210883
C	45	-0.079494	0.404577	-0.042263	-0.434826
C	46	-0.136095	0.527997	-0.065384	0.417376
N	47	-0.244046	0.544572	-0.147673	-0.560105
C	48	-0.292018	0.436698	-0.205014	0.356461
C	49	-0.292845	0.186783	-0.237665	-0.203861
C	50	-0.282251	-0.048238	-0.246547	-0.506295
C	51	-0.384144	-0.048831	-0.338402	0.419674
N	52	-0.443103	0.066660	-0.383991	-0.588516
C	53	-0.397300	0.179022	-0.334256	0.402180
H	54	0.006004	0.393674	0.023285	0.217991
H	55	-0.096119	0.616796	-0.017891	0.044551
H	56	-0.378553	0.449565	-0.269568	0.062469
H	57	-0.237532	-0.140704	-0.212022	0.199764
H	58	-0.422332	-0.142277	-0.377878	0.034175
H	59	-0.441882	0.271031	-0.371425	0.051579

Supplementary Table 14: $[Ru(bpy)_2(bpz)]^{3+}$ p-T_1 structure and ESP partial charges used for the MD simulations.

Atom	Position	x	y	z	ESP
Ru	1	-0.110303	-0.013808	-0.054302	0.064095
N	2	-0.013905	0.060829	-0.222817	0.020498
N	3	0.084116	-0.089706	-0.029210	-0.024570
C	4	0.121195	0.039258	-0.227855	0.066892
C	5	-0.072471	0.139346	-0.315307	-0.073288
C	6	0.000446	0.204326	-0.414327	-0.034639
C	7	0.138591	0.186696	-0.417914	-0.000786
C	8	0.198971	0.102220	-0.324631	-0.095885
C	9	0.173701	-0.052536	-0.125450	0.108210
C	10	0.123099	-0.174803	0.068105	0.005607
C	11	0.251047	-0.228674	0.073020	-0.065784
C	12	0.343314	-0.192115	-0.025121	0.027466
C	13	0.303993	-0.102775	-0.124544	-0.119317
H	14	-0.179429	0.151349	-0.306550	0.182309
H	15	-0.050719	0.268423	-0.485315	0.147814
H	16	0.199261	0.236465	-0.492763	0.161319
H	17	0.306148	0.087062	-0.327277	0.151694
H	18	0.048531	-0.201186	0.142169	0.087457
H	19	0.276117	-0.298965	0.151619	0.147916
H	20	0.443888	-0.233093	-0.024834	0.151712
H	21	0.373869	-0.073857	-0.202068	0.145961
N	22	-0.050880	0.144391	0.069892	0.061508
N	23	-0.188110	-0.073353	0.131620	-0.053102
C	24	-0.076874	0.126998	0.202828	-0.040003
C	25	0.016712	0.253878	0.029066	-0.077554
C	26	0.062378	0.350182	0.118251	-0.040156
C	27	0.038542	0.332201	0.254394	0.019581
C	28	-0.032119	0.219970	0.296969	-0.101565
C	29	-0.154221	0.006490	0.237058	0.238370
C	30	-0.265626	-0.181498	0.153191	0.020994
C	31	-0.309524	-0.217397	0.280003	-0.102873
C	32	-0.272196	-0.138818	0.388917	0.069030
C	33	-0.194353	-0.025558	0.366884	-0.195581
H	34	0.033681	0.264441	-0.077275	0.168751
H	35	0.116068	0.436457	0.080569	0.139359
H	36	0.074590	0.404657	0.326710	0.121159
H	37	-0.051760	0.204789	0.402402	0.159167
H	38	-0.291697	-0.240078	0.065874	0.083583
H	39	-0.372615	-0.304369	0.292840	0.143792
H	40	-0.303605	-0.164557	0.489441	0.133350
H	41	-0.165820	0.036903	0.450438	0.188428
N	42	-0.304279	0.048768	-0.100871	0.014585
N	43	-0.179782	-0.174259	-0.169532	0.256521
C	44	-0.377976	-0.042716	-0.178068	0.032330
C	45	-0.366456	0.165653	-0.067134	-0.085392
C	46	-0.492396	0.197451	-0.109276	-0.016457
N	47	-0.561347	0.106214	-0.187760	-0.100582
C	48	-0.505427	-0.012210	-0.220696	-0.023319
C	49	-0.309019	-0.164860	-0.212984	-0.130188
C	50	-0.107791	-0.282742	-0.206063	-0.369113
C	51	-0.164645	-0.382880	-0.283122	0.430431
N	52	-0.293090	-0.377888	-0.323116	-0.512988
C	53	-0.363329	-0.271300	-0.288702	0.373906
H	54	-0.311295	0.235263	-0.005359	0.156482
H	55	-0.540653	0.291427	-0.086463	0.168121
H	56	-0.564515	-0.077887	-0.283202	0.192845
H	57	-0.005168	-0.288763	-0.171965	0.182126
H	58	-0.106223	-0.469466	-0.312732	0.051188
H	59	-0.466791	-0.268549	-0.321995	0.067196
H	60	-0.652248	0.129600	-0.226365	0.321387

Supplementary References

[1] Kazuteru Shinozaki, Youkoh Kaizu, Hiroyuki Hirai, and Hiroshi Kobayashi. Protonation and complex formation of [(2,2'-bipyrazine)bis(2,2'-bipyridine)ruthenium(ii)](2+) in the lowest excited state. *Inorganic Chemistry*, 28(19):3675–3679, 1989.

[2] D. Paul Rillema, G. Allen, T. J. Meyer, and D. Conrad. Redox properties of ruthenium(ii) tris chelate complexes containing the ligands 2,2'-bipyrazine, 2,2'-bipyridine, and 2,2'-bipyrimidine. *Inorganic Chemistry*, 22(11):1617–1622, 1983.

[3] Hai Sun and Morton Z. Hoffman. Protonation of the excited states of ruthenium(ii) complexes containing 2,2'-bipyridine, 2,2'-bipyrazine, and 2,2'-bipyrimidine ligands in aqueous solution. *The Journal of Physical Chemistry*, 97(19):5014–5018, 1993.

[4] Ivo H.M. van Stokkum, Delmar S. Larsen, and Rienk van Grondelle. Global and target analysis of time-resolved spectra. *Biochimica et Biophysica Acta (BBA) - Bioenergetics*, 1657(2):82–104, 2004.

[5] Ricardo J. Fernández-Terán, Estefanía Sucre-Rosales, Lorenzo Echevarria, and Florencio E. Hernández. A sweet introduction to the mathematical analysis of time-resolved spectra and complex kinetic mechanisms: The chameleon reaction revisited. *Journal of Chemical Education*, 99(6):2327–2337, Jun 2022.

[6] Kasper Skov Kjær, Tim B. van Driel, Jan Kehres, Kristoffer Haldrup, Dmitry Khakhulin, Klaus Bechgaard, Marco Cammarata, Michael Wulff, Thomas Just Sørensen, and Martin M. Nielsen. Introducing a standard method for experimental determination of the solvent response in laser pump, x-ray probe time-resolved wide-angle x-ray scattering experiments on systems in solution. *Phys. Chem. Chem. Phys.*, 15:15003–15016, 2013.

[7] William Humphrey, Andrew Dalke, and Klaus Schulten. VMD – Visual Molecular Dynamics. *Journal of Molecular Graphics*, 14:33–38, 1996.

[8] Severin Polonius, Leticia González, and Sebastian Mai. Ultrafast solvent migration in an iron complex revealed by nonadiabatic dynamics simulations. *Chem. Sci.*, 16:11128–11137, 2025.
