# Supplementary material for: Electronic and solvent reorganization in proton-coupled electron transfer captured by ultrafast X-rays
Source: Nat Commun. 2026 Aug 26;17:9053. doi: 10.1038/s41467-026-75943-4 (PMC13518934; doi:10.1038/s41467-026-75943-4)
Supplement: Supplementary file 2 [file 41467_2026_75943_MOESM2_ESM.pdf]

nature portfolio

Peer Review File

Electronic and Solvent Reorganization in Proton-Coupled Electron Transfer Captured by Ultrafast X-rays

Corresponding Author: Dr Elisa Biasin

This file contains all reviewer reports in order by version, followed by all author rebuttals in order by version.

Attachments originally included by the reviewers as part of their assessment can be found at the end of this file.

Version 0:

Reviewer comments:

Reviewer #1

(Remarks to the Author)
Please find the attached review report.

Reviewer #2

(Remarks to the Author)
The publication from Kahraman et al. claims to measure proton-coupled electron transfer using several ultrafast techniques which they trigger by light-activating a metal-centred complex (Ru(bpy)2(bpz))2+ that creates an MLCT state which results in excited-state protonation under acidic pH conditions. The paper includes ultrafast optical measurements (OTA), time-resolved N K-edge XAS and ultrafast solution scattering (TR-XSS) results, and is supported with simulations. I generally find the results interesting, well presented and they have compiled support from simulations for their conclusions, which is all good. But there are still some aspects I think should be addressed in the manuscript. One significant challenge is that the SI contains a lot of information, and much of it is relevant to be able to support the conclusions in the main text.

Perhaps the most general comment is that the three experiments were done under different excitation conditions (fluence and concentration primarily, but also wavelength for the TR-XSS) and as this directly affects the excited state concentration it will also affect the kinetics of the excited-state protonation reaction. This makes it more difficult to directly compare the different measurements. For example I think this means that forcing the N K-edge XAS data to use a 97 ps component, which is extracted from the optical measurements, isn't completely justified ? There is an excitation fraction of 0.5 mentioned in the SI for the XAS data, which is quite high and should then result in higher ES protonated concentration in the XAS measurements ? It might be useful to estimate some excitation values for the three different measurements, and the use this information to justify the kinetics. I mention this again in the TR-XSS comments below.

For the N K-edge measurements the experimental data and simulations match well, though the data shown in S7 that should not contain the excited-state protonation is less convincing, partially because the S/N is not as good. One recommendation to address this might be to overlay the different pH transient XAS data time points (1, 100, 300 ps) to emphasize the differences ? At first glance the data in Fig. 2B and S7 look quite similar so this additional presentation in the SI might help show that there are significant (i.e. outside the error bars) differences at late times. It would be even better if there was a time trace taken at 398.9 eV for the pH7 data that could be shown, though that may not have been collected. Another comment, which is somewhat cosmetic, is that I don't particularly like the presentation of the GS XAS as an inverted spectrum in Fig 2B, I find that a bit confusing. Presumably this is because the authors want to emphasize the changes in shape of the spectrum, I'm not so sure it makes things any clearer.

As my last comment from my perspective the TR-XSS is the least well-supported of the data shown. This is at least partially because I don't see an explanation as to how scattering in the 0.25-0.35 1/Å range can be directly related to protonation of the bpz ligand. The length scales for the pump-probe signals seem very long, while the RDFs in Fig. 3F seem mostly <3 Å which presumably means the changes being probed are over several solvation shells (few nm) ? There's a decomposition of the solute-solvent scattering in the SI (S15) but this doesn't really clarify very much since it appears the almost all parts of the solute contribute to the increase in low-q scattering signal (including the bpys). The authors also explicitly mention that they

can't explain the MLCT bpz data (pH 7, unprotonated ES) which shows an increase in signal as well and defer this analysis to a 'future work' which is very unsatisfying for the reader since it means that at least some of this signal can't be explained. Finally the TR-XSS data shows a continual increase in the low-q scattering signal out to 300 ps, which is not really consistent with the 97 ps component from the OTA. There are fits in Fig. 3C though they're mentioned as 'guide to the eye' but I don't think there are any time constants reported ? This isn't what I would necessarily expect the signal to do, though if the kinetic model I suggest above was included it might be explained by a lower excited-state concentration resulting in a slower reaction rate ? Or perhaps simply presenting the TR-XSS data using the 'log' time-scale used for the OTA and XAS measurements would help ? For the moment I would be most skeptical about this data supporting the ES protonation results, at least with the current presentation.

Reviewer #3

(Remarks to the Author)
This study presents a combined ultrafast investigation of PCET dynamics in a Ru complex. The authors used transient absorption in the optical regime (OTA), ultrafast X-ray absorption at the N K-edge (XAS), as well as time-resolved X-ray solution scattering (TRXSS) to probe the same system, aiming to reveal a complete picture of both the chemical kinetics and the solvent reorganization of the studied system. Considerable experimental and theoretical effort has been devoted to this work, and it indeed demonstrates that combining three different techniques, each with its own unique capabilities, can provide a detailed understanding of a specific system. I believe this work is worthy of publication in Nat Commun; however, I have several concerns that should be addressed first:

1. OTA and XAS appear to have been carried out with 400 nm excitation, which excites the system to MLCT_bpy, whereas TRXSS was performed with 530 nm excitation, which I believe excites to MLCT_bpz. In principle, the molecule therefore starts from a different initial state in the TRXSS experiment compared to OTA and XAS. How would this difference affect the conclusions, which are currently drawn as if all measurements probe the same photoexcited dynamics? I recognize that MLCT_bpy rapidly undergoes internal conversion to MLCT_bpz within 180 fs, making it potentially reasonable to assume similar dynamics at long delay times. However, it remains unclear whether the internal conversion and the different excess kinetic energy introduced by the pump lasers across these measurements influence the dynamics and the final conclusions.

2. Given that the MLCT_bpy → MLCT_bpz process occurring within 180 fs is not investigated, what is the motivation for exciting the molecule at 400 nm instead of 474 nm or 530 nm in the OTA and XAS experiments?

3. The authors conclude that XAS is more sensitive to protonation than OTA, which is not entirely clear to me. When examining the time-resolved spectra from both measurements, neither shows clear peak shifts; instead, the primary change from 1 ps to 100 ps is in peak magnitude in both measurements. From a purely experimental perspective, I do not see a clear distinction between the two techniques. It seems that this conclusion is mainly based on the fact that TDDFT calculations of XAS are easier to interpret in terms of protonation than TDDFT calculations of OTA. If so, this seems to be more of a computational or theoretical interpretation issue rather than an intrinsic advantage of XAS.

Overall, I think this study would make a valuable contribution to the field, but the concerns above can be clarified to improve its credibility and accessibility to the community.

Reviewer #4

(Remarks to the Author)
I have reviewed a previous version of this manuscript for a different journal. I find only small changes in wording compared to the previous version, and therefore use a modified version of my previous review.
The manuscript presents a study of a well-known photo-basic Ru(II)(bpy)2(bpz)-complex in aqueous solution by time-resolved (TR) optical and X-ray absorption spectroscopy (TR-XAS), TR X-ray scattering and calculations. The photobasic complex is known to become protonated after excitation, if the solution pH is below pKa* = 3.5. Thus, the authors could confirm the cycle of excitation-protonation-ground state recovery at pH = 1 with TR-optical spectroscopy, with a time constant for protonation in the excited state of ca. 100 ps. Their TR-XAS data showed significant instantaneous changes due to excitation, followed by only small further changes up to the last spectrum recorded at 300 ps. With the help of TDDFT, the authors propose those changes are due to the expected protonation of the bpz ligand. In separate experiments, TR X-ray scattering experiments showed increased scattering at low Q-values for the excited state, and even more so for the protonated excited state, which was supported by simulations.
The scientific questions underlying the study are of high interest, and the TR-experiments are performed, analyzed and computationally supported by experts. I am nevertheless not convinced that the present manuscript is suitable for Nature Comm.

1. A major weakness is that the TR-XAS data does not clearly show that PT occurs. This is in clear contrast to the stated aims of the study. The 398 eV peak does not change during PT, and the 399 eV peak goes down very little in comparison to the TDDFT results; with a 97 ps lifetime, most of the decay would have taken place already at 100 ps. As these are the only changes that are supposed to report on PT, this is a significant weakness. I have seen much more convincing evidence for PT in PCET reactions by TR optical TA and photoluminescence data. Thus, the method fails to follow PT, which is the main point of the manuscript.

In addition, there are strong peaks at 400.5-401 eV in the GS as well as at all times after excitation, but these are not

discussed.

2. The authors state on p. 3 that there are no previous time-resolved (TR) optical studies of this complex. This is clearly incorrect. The authors themselves cite data from TR-PL and optical transient absorption studies in ref 35 and 40 and there are many more of exactly this complex, and closely related Ru(II) photobases, e.g. J. Am. Chem. Soc. 129 (2007) 6968 and J. Phys. Chem. A 2022, 126, 27, 4349–4358. Moreover, the processes and time scales reported in the manuscript are already known, meaning that any novelty must lie in the method, not in the conclusions.

3. Photobases have been studied in detail before, including ultrafast studies with TRIR and TR-PL (see e.g. JACS, 2023, 145, 6612) and have shown considerable mechanistic complexity, far beyond what is reported here by TR-XAS/-XSS. These studies have typically used strong photobases (pKa of H+Base* >13) so that H2O or weak acids are the primary proton donors. In the present study, the weak basicity (pKa of H+Base* = 3.5) and acidic pH means that H3O+(aq) must be the primary donor, and the ca. 100 ps time constant for the proposed protonation fits well with the expected diffusion-controlled protonation by H3O+ at pH = 1 (kp = 1.4E11 M-1s-1). The nature of the primary donor is not discussed, nor if this makes an important difference to the previous studies.

4. The authors motivate the study by the challenges to distinguish different PCET pathways, referring to the degree of concertedness of PT and ET. The problem is typically not the lack of unique signal of the proposed intermediate, however, but that the intermediate often does not accumulate to a detectable degree in an incoherent ensemble measurement. Then TR-XAS is not better than TR in any other spectral regime. It is also difficult to prove the non-existence of an intermediate by direct methods; as far as I am aware, it can only be inferred from comparisons. Moreover, in the current study, the authors choose a photo-base as system, where the protonation cannot be concerted with the excitation, because of the large difference in time scales, so the distinction of mechanisms is not relevant for the present study. Therefore, this study does not convince this reader that progress is made on this specific point either.

Version 1:

Reviewer comments:

Reviewer #1

(Remarks to the Author)
The authors performed additional experiments that were suggested, significantly improved the writing and explanations throughout the manuscript following the reviewers' suggestions. Considering all changes that have been made, I think this article can now be accepted.

Reviewer #2

(Remarks to the Author)
I thank the manuscript authors for their efforts in addressing my comments in their resubmission. I think the paper is very much improved in the revised form and I have no further comments or recommendations to make. I think this draft is suitable for publication in Nat Comm.

Reviewer #3

(Remarks to the Author)
All my previous comments have been addressed in the revision. I support the publication of the paper.

Reviewer #4

(Remarks to the Author)
The authors have taken the comments and suggestions form all reviewers very seriously, and made significant improvements, both with additional data and modified text. I therefore agree that the manuscript is ready for publication.

I do agree that TR-Xray spectroscopy and scattering are very important tools.
I still find the present data XAS to be a bit weak in showing the protonation that we know must occur. I realize that the manuscript is on method development, and I am sure the author group will contribute both more convincing and more challenging results in the near future.

Open Access This Peer Review File is licensed under a Creative Commons Attribution 4.0 International License, which permits use, sharing, adaptation, distribution and reproduction in any medium or format, as long as you give appropriate credit to the original author(s) and the source, provide a link to the Creative Commons license, and indicate if changes were made.
In cases where reviewers are anonymous, credit should be given to 'Anonymous Referee' and the source.
The images or other third party material in this Peer Review File are included in the article's Creative Commons license, unless indicated otherwise in a credit line to the material. If material is not included in the article's Creative Commons license and your intended use is not permitted by statutory regulation or exceeds the permitted use, you will need to obtain permission directly from the copyright holder.
To view a copy of this license, visit https://creativecommons.org/licenses/by/4.0/

<u>Review report for NCOMMS-26-004071-T</u>

We thank all reviewers for carefully reading our manuscript and for their insightful comments, which we believe have significantly improved the manuscript.

The reviewers' comments prompted us to revisit the optical transient absorption (OTA) measurements. In the original experiments, a calibrated pH meter was not available. New measurements performed under controlled pH 1 conditions show that the protonated species rises with a time constant of 460 ps and decays with a ~3 ns lifetime. We therefore conclude that the previously reported measurements were performed at a more acidic pH than intended.

We believe this clarification resolves the apparent discrepancy between the OTA and X-ray measurements (the latter were carried out under well-controlled pH conditions).

First, the evolution of the N K-edge XAS signal (specifically the decay of the transient positive feature at 398.9 eV) does not reach baseline at 300 ps, in contrast to the simulated response. The revised lifetime indicates that the maximum protonated population is reached on the nanosecond timescale, and that only ~60% of this population is probed at 300 ps. This resolves the discrepancy between measured and calculated XAS spectra, and further supports the assignment of the decrease of the 398.9 eV signal to the protonated species.

Second, the kinetic traces in the X-ray scattering data exhibit a plateau on the nanosecond timescale, which is now fully consistent with the updated OTA results.

Figures 1B,C. Figure 2C and the corresponding captions and discussion in the main text have been updated to reflect the new OTA measurements.

Below, we provide a point-by-point response to the reviewer's comments. We use blue bold text for our responses to the reviewer's comments, and black italic text with red highlights to indicate changes made to the manuscript.

Reviewer 1

The article titled "*Electronic and Solvent Reorganization in Proton-Coupled Electron Transfer Captured by Ultrafast X-rays*" by Kahraman, A., Larsen, C. and Biasin, E *et al*. utilizes transient absorption, time-resolved N K-edge and time-resolved X-ray solution scattering methods to investigate proton transfer step in the triplet metal-to-ligand charge transfer excited state of a Ru(II) chromophore, $[Ru(bpy)_2(bpz)]^{2+}$. The results presented in herein establishes proton transfer timescales from ^{3}MLCT excited state to be ~100 ps – suggesting diffusion-based

protonation step and I am very intrigued by the TR-XSS results and simulations that revealed clear solvent reorganization during the proton transfer step.

This manuscript is well written, and the results are mostly in support to the authors conclusions. I believe this will be an important contribution in this field; however, several clarifications need to be addressed before I can recommend this article for publication at *Nature Communications*.

We thank the reviewer for their insightful comments.

My specific comments as presented below and are not prioritized in terms of significance.

1. Please add characterization data (^{1}H NMR, ^{13}C NMR etc) of the complex even though it was prepared using a reported procedure.

We have added details of the sample preparation, as well as the ^{1}H NMR data, in SI Section 1.1

2. Throughout the manuscript the authors highlight importance of these results for mechanistic understanding of PCET process. From page 2, line 37: "*Our choice of a system that undergoes sequential PCET enables clear separation of the timescales of electron and proton transfer,*" the paper clearly addresses proton transfer step, however the timescale of electron transfer is not obvious and not discussed with clarity, and therefor creates confusion on the PCET introduction. While with proton this is extremely challenging, I wonder if the authors have tried or have any future plan to perform similar experiments with a coupled donor-acceptor type system where electron transfer and proton transfer steps can be distinguished with more clarity.

In $[Ru(bpy)_2(bpz)]^{2+}$, the electron transfer step corresponds to the photoinduced metal-to-ligand charge transfer (MLCT), in which an electron is transferred from the Ru center to the bpz ligand. Our OTA measurements show that this process occurs within our instrument response function of ~180 fs. By the time we probe the system at 1 ps in the X-ray experiments, the electron is already fully localized on the bpz ligand in the ^{3}MLCTbpz state, and the subsequent proton transfer proceeds on a separate timescale.

Regarding the reviewer's suggestion of coupled donor-acceptor systems: we fully agree that this is an important and exciting direction. Indeed, a central motivation of the present work is to establish the multimodal framework on a system where the steps are clearly separated to then extend it to more complex systems where ET and PT are coupled.

We have clarified these points in the Introduction:

We herein integrate femtosecond optical spectroscopy, N K-edge ultrafast XAS, and TR-XSS, together with advanced electronic structure and molecular dynamics simulations, to investigate the distinct steps of ~~sequential PCET~~ charge and proton transfer in the model complex

$[Ru(bpy)_2(bpz)]^{2+}$. In this model system the charge and proton transfer steps are sequential and well separated in timescale: the photoinduced electron transfer occurs within our instrument response function, while proton transfer follows on a slower, diffusion-limited timescale in the hundreds of picoseconds regime. Our choice of a system that undergoes sequential PCET , ~~enables~~ with clear separation of the timescales of electron and proton transfer, mak~~ing~~es the spectral signatures associated with each step more interpretable.

{…}

This system provides an ideal mechanistic reference point to test our multimodal approach and for extending it to more complex PCET systems, including bimolecular reactions, systems with unknown pathways, and cases in which electronic and/or nuclear degrees of freedom are more strongly coupled.

3. On page 3, line 59, the authors claim that time-resolved studies have not been carried out on this Ru(II) system, however reference 40 performed time-resolved emission measurements on this system. While I am aware of the distinction of this work as compared to the ref 40, for broader audience, a necessary adjustment to the sentence is requested.

We have modified the sentence as followed:

~~Time-resolved optical studies, which have not been previously reported for this system, are necessary to set the initial conditions for the X-ray mechanistic studies.~~

Time-resolved emission measurements and single point transient absorption have been reported for this system[34,35,36]. Here, we conduct broadband transient absorption to capture the full spectral evolution and establish the excited-state initial conditions for the X-ray experiments.

4. Figure 1(A): The authors assign the 2-3 ps time constant for singlet-to-triplet conversion, which do not align well with previous reports on Ru(II) polypyridyl complexes (McCusker, J. et al. Science 1997, Okada, T. et al. JACS 2002 and many others). In fact, the authors commented on this time component in the main text and assigned it to a vibrational cooling process, so it just may be a printing error and should be fixed.

The reviewer is correct, and we thank them for pointing this out. The 2–3 ps time constant was incorrectly assigned to intersystem crossing in Figure 1A, whereas it corresponds to vibrational relaxation, as discussed in the main text. We have corrected this assignment in the revised figure.

5. The authors assign the ~97 ps component as a new species based on the change in lifetimes and ESA bands. To me, the kinetic data is more evident for such assignment and

need to be discussed in the main paper. At pH 1, the kinetics monitored at 376 nm, shows a change in sign with a similar time constant (~100 ps) [*also in the Kinetic data probed at 380 nm*
in Figure S4]. A change in sign in a TA kinetics can only be explained by change in electronic state or evolution of a different species with different molar extinction coefficient.

We agree with the reviewer that the change in sign in the TA kinetics is indicative of formation of a new species. We have clarified our language on this point to show that the overall spectral reshaping is what evidence formation of a new species, with the global fitting then providing the time constants for formation and decay of this new species:

At pH 1, the early dynamics mirror those at pH 7, followed by spectral evolution over the first few hundred picoseconds (Figure 1B,C), indicating formation of a new species. The transient absorption kinetics at the ~350 nm ESA band exhibit a clear change in temporal behavior on a hundreds-of-picoseconds timescale, transitioning from an initial rise to a subsequent decay (Figure 1C). Global analysis [39] of the pH 1 OTA data (Figure S3) indicates that this new species forms with a time constant of ~~~97~~ 460 ps and decays with an accelerated lifetime of ~~~2.9~~ 3.3 ns. The latter is consistent with a previous report on the decay of the non-emissive excited-state of $[Ru(bpy)_2(bpz)]^{2+}$ in acidic solution [40]. The OTA spectra at ~~100 ps and~~ 300 ps and 1 ns in Figure 1B show that this new species is spectrally similar to the $^3MLCT_{bpz}$ state, but the ESA at ~350 nm blue-shifts and decreases in amplitude. As this ESA is attributed to the reduced bpz, its spectral change is thus consistent with a change in electronic structure of the bpz ligand upon protonation, but provides no further detail on the electronic redistribution. Our time-dependent density functional theory (TDDFT) calculations reproduce the OTA observables (Figure S6). However, valence TDDFT spectra are difficult to interpret because of the high degree of delocalization and mixed character of the excitations. To directly capture electronic redistribution at the ligand level, N K-edge XAS is necessary.

6. While it is unrealistic to ask to perform XAS experiments in D_2O/DCl, have the authors performed TA experiments to further support diffusion-based proton transfer assignment to the 100 ps time constant?

We thank the reviewer for this suggestion. We have now performed additional transient absorption measurements as a function of isotopic substitution, included in the revised Supporting Information (new Figure S5). We find no measurable kinetic isotope effect at pH 1, while at pH 0 a small KIE (~1.3) emerges. This behavior is consistent with a diffusion-limited proton transfer process.

We have modified the main text as following:

~~At pH 1, this corresponds to a quenching constant kq of ~1×1011 M−1 s−1, consistent with diffusion limited kinetics.~~ Additional OTA measurements as a function of pH and isotopic

substitution (Figure S5) support a diffusion-limited mechanism for the formation of the protonated excited state.

7. In Figure S3, it seems like the relative intensity of ESA and GSB bands for 400 nm and 510 nm photoexcitation changes. This is further evident from the amplitudes of the kinetic plots. Can the authors comment of this observation? A zoomed in plot presenting the dynamics in the 0 – 20 ps timescales would be a good addition to the paper.

We have added the requested plot, along with an overlay of the evolution-associated difference absorption spectra for the two datasets in Figure S4. The new plots confirm that the transient response at >180 fs is identical regardless of excitation wavelength.

8. One would expect to observe blue shift in the XAS after protonation as compared to the MLCT state – as also predicted by the computation, however from the XAS data this is not obvious. Can the authors discuss why the blue shift is not seen experimentally?

The reviewer is correct that TDDFT predicts a blue-shift of the N 1s→π* transition at the protonated nitrogen, arising from stabilization of the N 1s core level upon protonation. However, this shift is only apparent when considering the individual transitions of the protonated nitrogen in isolation (dark purple trace in Figure 2G). When examining the total low-energy XAS feature, which contains contributions from multiple nitrogen atoms, no net blue-shift is observed, either in the calculations or in the experiment.

This behavior arises from two competing effects at the protonated site: while the transition energy shifts to higher energy, the corresponding oscillator strength is significantly reduced due to localization of the excited-state electron density at the protonated nitrogen. As a result, the protonated site contributes a weak, blue-shifted transition that does not produce a discernible positive transient on the high-energy side of the peak. This contrasts with the MLCT state, where the low-energy feature shifts as a whole.

We have revised the manuscript to clarify this point:

The protonated N (dark-purple) shows blue-shifted transitions due to 1s orbital stabilization by the added positive charge, along with reduced oscillator strengths relative to the 1GS and 3MLCTbpz states due to the accumulation of the electron density at its site. ~~*Collectively, these effects reduce the intensity of the low-energy XAS peak without generating a positive transient on its high-energy shoulder, whereas in the 3MLCTbpz state, a positive transient appears due to the peak's blue-shift. These trends are consistent with the experimentally observed reduction of the 398.9 eV transient upon protonation (Figure 2C).*~~
Although the transition of the protonated N blue-shifts, the simultaneous reduction in oscillator strength renders it too weak to generate a positive transient above the ground-

state baseline on the high-energy shoulder of the peak. The net observable effect is therefore a reduction in the overall intensity of the low-energy XAS peak, without an accompanying blue-shift. This contrasts with the ^{3}MLCTbpz state, where the low-energy XAS peak blue-shifts as a whole and the oscillator strengths remain significant, producing a positive transient on the high-energy shoulder. These trends are consistent with the experimentally and theoretically observed positive transient at 398.9 eV in the ^{3}MLCTbpz state and its subsequent decrease upon protonation (Figure 2C).

9. ChemDraw perspective structures presented in Figure 1A and 3D are not consistent and should be consistent throughout the manuscript.

We thank the reviewer for pointing out this inconsistency. In response to a comment from Reviewer 2, we have removed the ChemDraw cartoon previously shown in Figure 3D, which resolves the issue.

10. Could the authors elaborate the choice of this chromophore for this particular study (beyond the one sentence mentioned in the last paragraph of the introduction of the paper).

In addition to the text already added regarding the separation of timescales, we have further clarified why this system is a particularly suitable candidate for X-ray scattering experiments by adding the following text in the Introduction:

We further select $[Ru(bpy)_2(bpz)]^{2+}$ since it undergoes only minimal intramolecular structural changes upon photoexcitation. In combination with the strong X-ray scattering contrast of Ru, this provides a favorable TR-XSS regime: weak intramolecular structural response suppresses geometric contributions to the signal, while the Ru center enhances sensitivity to subtle solute–solvent distance changes. As a result, this combination simplifies the interpretation of the scattering response and enables a clearer view of solvent reorganization coupled to each step of the reaction.

Reviewer #2 (Remarks to the Author):

The publication from Kahraman et al. claims to measure proton-coupled electron transfer using several ultrafast techniques which they trigger by light-activating a metal-centred complex (Ru(bpy)2(bpz))2+ that creates an MLCT state which results in excited-state protonation under acidic pH conditions. The paper includes ultrafast optical measurements (OTA), time-resolved N K-edge XAS and ultrafast solution scattering (TR-XSS) results, and is supported with simulations. I generally find the results interesting, well presented and they have compiled support from simulations for their conclusions, which is all good. But there are still some aspects I think should be addressed in the manuscript.

One significant challenge is that the SI contains a lot of information, and much of it is relevant to be able to support the conclusions in the main text.

Perhaps the most general comment is that the three experiments were done under different excitation conditions (fluence and concentration primarily, but also wavelength for the TR-XSS) and as this directly affects the excited state concentration it will also affect the kinetics of the excited-state protonation reaction. This makes it more difficult to directly compare the different measurements. For example I think this means that forcing the N K-edge XAS data to use a 97 ps component, which is extracted from the optical measurements, isn't completely justified ? There is an excitation fraction of 0.5 mentioned in the SI for the XAS data, which is quite high and should then result in higher ES protonated concentration in the XAS measurements ? It might be useful to estimate some excitation values for the three different measurements, and the use this information to justify the kinetics. I mention this again in the TR-XSS comments below.

We thank the reviewer for the insightful comment.

Indeed, the OTA and X-ray experiments operate in fundamentally different concentration regimes. In the OTA experiments (~0.01 mM, ~ 0.02 excitation fraction), we showed that the protonation rate is consistent with diffusion-limited kinetics, implying a near-unity probability of reaction per bimolecular collision.

In contrast, the XAS experiment is performed at much higher solute concentration (~65 mM) and an excitation fraction of ~0.5, resulting in an excited-state concentration of ~32.5 mM. Under these conditions, the excited-state concentration becomes comparable to $[H^+]$ = 0.1 M, and the pseudo-first-order approximation is no longer strictly valid. The kinetics are therefore better described by a second-order rate law:

$$\frac{d[\mathbf{Ru}^*]}{dt} = -k_q[\mathbf{Ru}^*][\mathbf{H}^+]$$

with analytical solution:

$$[\mathbf{Ru}^*](t) = [\mathbf{Ru}^*]_0 \cdot \frac{[\mathbf{H}^+]_0 - [\mathbf{Ru}^*]_0}{[\mathbf{H}^+]_0 \exp\left(k_q([\mathbf{H}^+]_0 - [\mathbf{Ru}^*]_0)t\right) - [\mathbf{Ru}^*]_0}$$

where
$[\mathbf{Ru}^*]_0 = 0.0325$ **M,**
$[\mathbf{H}^+]_0 = 0.1$ **M,**
$k_q = 2.163 \times 10^{10}$ $\mathbf{M^{-1}\ s^{-1}}$ **(obtained from the pH 1 OTA data)**

Solving numerically for the condition $[\mathrm{Ru}^*] = [\mathrm{Ru}^*]_0/e$ yields an effective protonation timescale of approximately 530 ps under the XAS conditions. This is ~15% longer than the ~460 ps obtained in the OTA and remains well within the experimental uncertainty.

We also note that the XAS kinetic trace is intrinsically less well constrained than the OTA kinetics due to the lower signal-to-noise ratio. As a result, independent fitting of the XAS kinetics is not robust. The revised fit using a fixed 460 ps time constant (new panel C in Figure 2) reproduces the data equally well as before. Importantly, the primary role of the XAS kinetics is not precise extraction of a time constant (which is more reliably obtained from the OTA data) but rather to demonstrate the different spectral evolution at the ESA and GSB, showing population of the protonated species at pH 1.

In conclusion, we believe that fixing the 460 ps time constant in the XAS kinetic analysis is a justified approximation. We have modified the main text as following:

These dynamics are consistent with the kinetic model derived from the OTA data (solid lines in Figure 2C). We note that, due to the higher-concentration regime of the XAS experiment, we evaluated the impact of the full second-order kinetics to the protonation timescale, which results in only a modest (~15%) deviation from the pseudo-first-order description. This difference remains within the uncertainty of the XAS kinetics, and the data are therefore adequately described using the same effective time constant.

For the TR-XSS data, we use a concentration of 25 mM and a ~0.3 excitation fraction. The XSS kinetics are discussed in the following response. Regarding excitation wavelength, TR-XSS uses 536 nm excitation instead of 400 nm as in OTA and XAS. We performed OTA measurements at both 400 and 500 nm (Figures S4) and confirmed that there is no measurable excitation-wavelength dependence of the excited-state kinetics.

We have added the following in the XSS section:

To complement the electronic insights from OTA and N K-edge XAS, we performed a separate TR-XSS experiment (Figure 3~~A~~C). Figure 3~~B~~A shows transient scattering curves measured at 1 ps, 100 ps, ~~and~~ 300 ps and 1 ns after 536 nm ~~photo~~excitation of $[Ru(bpy)_2(bpz)]^{2+}$ at pH 1. At this wavelength, we directly excite the MLCT state localized on the bpz ligand and deposit less excess energy into the solvent compared to 400 nm excitation, which is preferred for TR-XSS experiments. As discussed above, we show that the excited-state dynamics on picosecond and longer timescales are independent of excitation wavelength (Figure S4).

For the N K-edge measurements the experimental data and simulations match well, though the data shown in S7 that should not contain the excited-state protonation is less convincing, partially because the S/N is not as good. One recommendation to address this might be to overlay the different pH transient XAS data time points (1, 100, 300 ps) to emphasize the differences ? At first glance the data in Fig. 2B and S7 look quite similar so this additional presentation in the SI might help show that there are significant (i.e. outside

the error bars) differences at late times. It would be even better if there was a time trace taken at 398.9 eV for the pH7 data that could be shown, though that may not have been collected. Another comment, which is somewhat cosmetic, is that I don't particularly like the presentation of the GS XAS as an inverted spectrum in Fig 2B, I find that a bit confusing. Presumably this is because the authors want to emphasize the changes in shape of the spectrum, I'm not so sure it makes things any clearer.

In response to the suggestion regarding Figure S7, we have added new plots in the SI comparing the pH 7 and pH 1 data for all three time delays (1, 100, and 300 ps). This is new Figure S8. These plots confirm that, for pH 1, a decrease of the signal at 399 eV occurs on the timescale of protonation, despite the lower S/N of the pH 7 data.

Regarding the kinetic traces at 398.9 eV for pH 7, these were unfortunately not collected, as the pH 7 measurements were taken at the beginning of the experimental run for alignment purposes.

Finally, we note the comment about the presentation of the ground-state XAS as an inverted spectrum in Figure 2B. We appreciate the feedback and have considered it; however, we believe that the current presentation is most illustrative and have thus retained it.

As my last comment from my perspective the TR-XSS is the least well-supported of the data shown. This is at least partially because I don't see an explanation as to how scattering in the 0.25-0.35 1/Å range can be directly related to protonation of the bpz ligand. The length scales for the pump-probe signals seem very long, while the RDFs in Fig. 3F seem mostly <3 Å which presumably means the changes being probed are over several solvation shells (few nm) ? There's a decomposition of the solute-solvent scattering in the SI (S15) but this doesn't really clarify very much since it appears the almost all parts of the solute contribute to the increase in low-q scattering signal (including the bpys). The authors also explicitly mention that they can't explain the MLCT bpz data (pH 7, unprotonated ES) which shows an increase in signal as well and defer this analysis to a 'future work' which is very unsatisfying for the reader since it means that at least some of this signal can't be explained. Finally the TR-XSS data shows a continual increase in the low-q scattering signal out to 300 ps, which is not really consistent with the 97 ps component from the OTA. There are fits in Fig. 3C though they're mentioned as 'guide to the eye' but I don't think there are any time constants reported ? This isn't what I would necessarily expect the signal to do, though if the kinetic model I suggest above was included it might be explained by a lower excited-state concentration resulting in a slower reaction rate ? Or perhaps simply presenting the TR-XSS data using the 'log' time-scale used for the OTA and XAS measurements would help ? For the moment I would be most

skeptical about this data supporting the ES protonation results, at least with the current presentation.

We thank the reviewer for this insightful and critical assessment of the TR-XSS data. The points raised have helped improve both the analysis and the presentation, in particular regarding what structural information can and cannot be reliably extracted from the data.

Regarding the interpretation of the low-Q signal, two aspects are relevant: the sensitivity of the signal to the first solvation shell and the sensitivity to rearrangement of the hydrogen bond network.

The low-Q region is sensitive to changes in solute–solvent correlations and therefore reflects modifications of the first solvation shell of the complex. This interpretation is consistent with previous studies on related transition-metal complexes, where low-Q differences have been assigned to first-solvent-shell reorganization around the solute.

We have added more references supporting this claim in the text:
The positive transient at low momentum transfer (Q) suggests a shortening of solute--solvent distances, as previously observed [27,28,29,47], likely reflecting strengthened hydrogen-bonding between the solvent and the reduced bpz ligand~~, as observed previously~~ [~~29~~,48].

The common heuristic relation r ~ 2π/Q should not be interpreted as a one-to-one mapping between a specific Q value and a unique interatomic distance. Instead, scattering features in Q-space and real space are connected through a Fourier transform relationship, meaning that each real-space contribution is distributed over a broad range in reciprocal space, and vice versa.

To make this connection more explicit, Fourier transforms of the simulated difference scattering curves (new Fig. 4E) show that the dominant structural changes are localized within 0 < R < 5 Å, consistent with modifications of the first solvation shell. In particular, the protonated state shows increased intensity in this range, indicating enhanced scattering-weighted correlations relative to the ground and MLCT states. Fourier transforms computed over both the full and experimentally limited Q ranges further show that, even with the restricted Q window (up to 3 $Å^{-1}$), the same qualitative sensitivity to first-shell structural changes is preserved. This is consistent with the differential radial distribution functions (new Fig. 4F), which show broad features in the 0 < R < 5 Å region.

We have added the following text to the manuscript:
Figure 3E shows the Fourier transform of the simulated difference scattering curves, providing a real-space representation of how scattering-weighted atom–atom pair correlations vary as a function of interatomic distance (R). Positive contributions correspond to an increase in

correlations at a given distance, whereas negative contributions indicate a reduction. The protonated state shows a marked enhancement of correlations in the 0 < R < 5 Å region relative to both the ground and ^{3}MLCTbpz states. This indicates that the calculated (and measured) signal is primarily sensitive to solute–solvent distances within the first solvation shell.

At the same time, we fully agree with the reviewer that the TR-XSS data does not allow for a unique decomposition into contributions from individual solute–solvent atom pairs. Even when using the full Q range in simulations, the resulting real-space signal in the region 0<R<5 Å remains a broad envelope with only weakly resolved features. In particular, contributions associated with changes around the protonated N (~ 3 Å) are not uniquely separable and are partially obscured by overlapping contributions from other atom pairs.

For this reason, we have revised the manuscript to avoid claiming local assignment from the data. Specifically, we (i) have removed the previous schematic cartoon that highlighted changes around the protonated N – this is now included in Figure 1A, and (ii) modified the RDF figure to include all relevant solute–solvent distances (new Fig. 4F). However, we also emphasize that the strong agreement between experimental data and simulations provides a robust validation of the underlying structural model. This combined experimental–theoretical approach enables atomistic-level insight, even if individual atom–pair contributions cannot be uniquely disentangled from the scattering data alone.

We have modified the text in the manuscript as following:
Abstract:

Additionally, we directly observe the rearrangement of the ~~solute–solvent hydrogen-bonding networks coupled~~ first-solvation-shell in response to these electronic changes.

Main text:

Having verified that the simulations reproduce our experimental observables, we next examine the changes in RDFs relative to the ground-state to gain atomistic insight into solvation-shell changes across the three electronic states. Figure 3F shows the difference RDFs of atom pairs expected to contribute most strongly to the scattering signal based on their electron density and abundance: ~~N-H_w and~~ N-O_w, C-O_w and Ru-O_w, where N and C comprises all N and C atoms of $[Ru(bpy)_2(bpz)]^{2+}$, while ~~H_w and~~ O_w represent ~~the H and O~~ the oxygen atoms in water, respectively.

~~In the $^3MLCT_{bpz}$ state, both N–H_w (2.0 Å) and N–O_w (2.8 Å) first-shell peaks increase relative to the ground state, consistent with strengthened hydrogen-bonding to the reduced bpz ligand [47, ?]. Upon protonation, the N–H_w peak decreases while the N–O_w peak increases, reflecting H-bond formation between the protonated bpz N and nearby O_w. This transition from N···H_wO_w to NH···O_w interactions (Figure 3D) implies a reorientation of water molecules around the~~

~~*protonated pyrazine. Since O is electron-richer than H, the rearrangement increases local electron density in the first solvation shell, contributing to the enhanced low-Q scattering signal.*~~

In the 3MLCTbpz state, a small positive feature appears in the N–Ow transient RDF at ~2.8 Å, consistent with strengthened hydrogen bonding to the reduced bpz ligand [47, 49]. Only minor changes are observed in the Ru–Ow and C–Ow RDFs, indicating limited solvation-shell changes in the MLCT state, in agreement with the weak transient signal observed experimentally and in simulations. Upon protonation, the N–Ow feature further increases, reflecting hydrogen-bond formation between the protonated bpz nitrogen and nearby water oxygen atoms. This corresponds to a change from N···HwOw to NH···Ow interactions (see also Figure S17). While individual atom–pair contributions cannot be resolved experimentally, atomistic simulations provide this structural interpretation. Positive transients in the Ru–Ow and C–Ow RDFs additionally suggest a general shift of water molecules closer to the solute. This rearrangement increases the effective electron density in the first solvation shell, contributing to the enhanced low-Q signal.

With respect to the discrepancy between the calculated and measured MLCT difference scattering signal, we note that the experimental signal associated with this state is extremely small (on the order of ~20 e.u./molecule), which is, to our knowledge, among the smallest reported in the literature. In this respect, the calculations reproduce the overall weak transient response, but underestimate part of the positive intensity at low Q.

To our knowledge, there are no studies that fully reproduce experimental solution scattering signals for MLCT states in polypyridyl complexes. Intramolecular structural changes in these systems are subtle compared to, for example, metal-centered states involving significant metal–ligand bond elongation, and solvation rearrangements are comparatively diffuse in the absence of strong hydrogen-bond restructuring around bpy ligands. From a computational perspective, quantitatively reproducing such weak signals requires accuracy beyond classical MD, while QM/MM or ab initio approaches remain limited by the extensive sampling required to converge scattering observables. For these reasons, we view the partial discrepancy as reflecting a broader, unresolved challenge in the field rather than a limitation specific to this work. Addressing it rigorously will require dedicated methodological development beyond the scope of the present study, which we intend to pursue in future work.

We have modified the main manuscript as following:

In the MLCTbpz state, reorientation of water near bpz C atoms -- qualitatively consistent with prior reports -- compensates for the strengthening of H-bonding to the bpz Ns, yielding a negligible net solute-solvent signal.~~This is in contrast with the small positive signal observed experimentally, and this discrepancy will be addressed in future work.~~ This differs slightly from the small positive signal observed experimentally, a point we will examine in future work, potentially requiring a higher level of theory to describe weak solute–solvent interactions

involving the reduced bpy ligand.

Finally, regarding the kinetic traces, we agree that presenting them as “guides to the eye” without clarification was not sufficiently clear, and we have removed the fitted curves from the figure. The kinetics extracted from TR-XSS are ‘consistent’ but not directly comparable to the time constants obtained from OTA and XAS, as the latter probe electronic-state population dynamics, whereas TR-XSS reflects nuclear (structural) rearrangements.

Reviewer #3 (Remarks to the Author):

This study presents a combined ultrafast investigation of PCET dynamics in a Ru complex. The authors used transient absorption in the optical regime (OTA), ultrafast X-ray absorption at the N K-edge (XAS), as well as time-resolved X-ray solution scattering (TRXSS) to probe the same system, aiming to reveal a complete picture of both the chemical kinetics and the solvent reorganization of the studied system. Considerable experimental and theoretical effort has been devoted to this work, and it indeed demonstrates that combining three different techniques, each with its own unique capabilities, can provide a detailed understanding of a specific system. I believe this work is worthy of publication in Nat Commun; however, I have several concerns that should be addressed first:

1. OTA and XAS appear to have been carried out with 400 nm excitation, which excites the system to MLCT_bpy, whereas TRXSS was performed with 530 nm excitation, which I believe excites to MLCT_bpz. In principle, the molecule therefore starts from a different initial state in the TRXSS experiment compared to OTA and XAS. How would this difference affect the conclusions, which are currently drawn as if all measurements probe the same photoexcited dynamics? I recognize that MLCT_bpy rapidly undergoes internal conversion to MLCT_bpz within 180 fs, making it potentially reasonable to assume similar dynamics at long delay times. However, it remains unclear whether the internal conversion and the different excess kinetic energy introduced by the pump lasers across these measurements influence the dynamics and the final conclusions.

We thank the reviewer for raising this important point. Indeed, the OTA and XAS experiments were performed with 400 nm excitation, which initially populates MLCTbpy, whereas the TR-XSS measurements at 536 nm directly excite MLCTbpz. As noted by the reviewer, however, the MLCTbpy state undergoes rapid internal conversion to MLCTbpz on a sub-180 fs timescale.

All measurements discussed here probe dynamics on picosecond timescales and longer, well after this internal conversion is complete. As such, the subsequent dynamics originate from the same MLCTbpz state in all cases.

We have further verified with OTA measurements (Figures S4) that the excited-state kinetics are independent of excitation wavelength (400 nm vs 500 nm) within our experimental uncertainty, indicating that differences in initial excess energy do not measurably affect the observed dynamics. Similarly, differences in excess energy deposition are not expected to significantly affect XAS measurements, which primarily probe electronic-state population dynamics. In contrast, TR-XSS is intrinsically sensitive to nuclear and solvent degrees of freedom, and include contributions from energy dissipation into the solvent. In this context, excitation at 536 nm, which directly populates the MLCTbpz state with reduced excess energy compared to 400 nm excitation, minimizes such contributions and allows for a more direct observation of structurally relevant solute–solvent correlations. This 'heating' signal is removed from the XSS data as described in the text, following well-established procedures.

We have added the following in the XSS section:

To complement the electronic insights from OTA and N K-edge XAS, we performed a separate TR-XSS experiment (Figure 3~~A~~C). Figure 3~~B~~A shows transient scattering curves measured at 1 ps, 100 ps, ~~and~~ 300 ps and 1 ns after 536 nm ~~photo~~excitation of [Ru(bpy)$_2$(bpz)]$^{2+}$ at pH 1. At this wavelength, we directly excite the MLCT state localized on the bpz ligand and deposit less excess energy into the solvent compared to 400 nm excitation, which is preferred for TR-XSS experiments. As discussed above, we show that the excited-state dynamics on picosecond timescales and longer are independent of excitation wavelength (Figure S4).

2. Given that the MLCT_bpy → MLCT_bpz process occurring within 180 fs is not investigated, what is the motivation for exciting the molecule at 400 nm instead of 474 nm or 530 nm in the OTA and XAS experiments?

At the time the N K-edge XAS experiment was performed at the chemRIXS endstation of LCLS, only harmonics of an 800 nm Ti:sapphire system were available, restricting the excitation wavelength to 400 nm. To ensure consistency, we carried out the OTA measurements under the same excitation conditions to directly support and compare with the X-ray experiment.

3. The authors conclude that XAS is more sensitive to protonation than OTA, which is not entirely clear to me. When examining the time-resolved spectra from both measurements, neither shows clear peak shifts; instead, the primary change from 1 ps to 100 ps is in peak magnitude in both measurements. From a purely experimental perspective, I do not see a clear distinction between the two techniques. It seems that this conclusion is mainly

based on the fact that TDDFT calculations of XAS are easier to interpret in terms of protonation than TDDFT calculations of OTA. If so, this seems to be more of a computational or theoretical interpretation issue rather than an intrinsic advantage of XAS.

Overall, I think this study would make a valuable contribution to the field, but the concerns above can be clarified to improve its credibility and accessibility to the community.

We thank the reviewer for this important comment. We agree that, in both OTA and XAS measurements, the dominant experimental change between early and late time delays is a modulation in signal amplitude rather than clear peak shifts. Our statement regarding "sensitivity to protonation" was not intended to imply a qualitative difference in the presence of spectral changes, but rather to refer to how directly each observable can be linked to the protonation event.

The key distinction between the two techniques lies in their degree of chemical and spatial specificity. N K-edge XAS is element- and site-specific, directly probing electronic structure changes at the nitrogen atoms, and we show here that we can distinguish the spectral changes due to the protonated nitrogen specifically. In contrast, OTA measures broad electronic absorption features that arise from multiple overlapping transitions and are not intrinsically site-specific, making direct assignment of individual spectral components to a given atomic site less straightforward.

We therefore clarify that the difference lies not in the presence of spectral changes, but in the extent to which those changes can be directly attributed to protonation at specific atomic sites. We have clarified this in the text, at the end of the XAS section:

Although protonation of the peripheral N is chemically intuitive, ~~our site-specific XAS measurements directly verify this assignment~~ the element- and site-specific nature of N K-edge XAS enables direct assignment of the observed spectral changes to the protonated nitrogen site, and reveal how protonation reshapes the electronic distribution of the excited states — a feature that has not been previously experimentally resolved.

Reviewer #4 (Remarks to the Author):

I have reviewed a previous version of this manuscript for a different journal. I find only small changes in wording compared to the previous version, and therefore use a modified version of my previous review.

The manuscript presents a study of a well-known photo-basic Ru(II)(bpy)2(bpz)-complex in aqueous solution by time-resolved (TR) optical and X-ray absorption spectroscopy (TR-XAS), TR X-ray scattering and calculations. The photobasic complex is known to become protonated after excitation, if the solution pH is below pKa* = 3.5. Thus, the authors could confirm the cycle of excitation-protonation-ground state recovery at pH = 1 with TR-optical spectroscopy, with a time constant for protonation in the excited state of ca. 100 ps. Their TR-XAS data showed significant instantaneous changes due to excitation, followed by only small further changes up to the last spectrum recorded at 300 ps. With the help of TDDFT, the authors propose those changes are due to the expected protonation of the bpz ligand. In separate experiments, TR X-ray scattering experiments showed increased scattering at low Q-values for the excited state, and even more so for the protonated excited state, which was supported by simulations.
The scientific questions underlying the study are of high interest, and the TR-experiments are performed, analyzed and computationally supported by experts. I am nevertheless not convinced that the present manuscript is suitable for Nature Comm.

We thank the reviewer for reviewing our manuscript. We are pleased that they find the scientific questions of high interest, and we hope that the modifications made to the manuscript will convince them that it is suitable for publication in *Nature Communications*.

1. A major weakness is that the TR-XAS data does not clearly show that PT occurs. This is in clear contrast to the stated aims of the study. The 398 eV peak does not change during PT, and the 399 eV peak goes down very little in comparison to the TDDFT results; with a 97 ps lifetime, most of the decay would have taken place already at 100 ps. As these are the only changes that are supposed to report on PT, this is a significant weakness. I have seen much more convincing evidence for PT in PCET reactions by TR optical TA and photoluminescence data. Thus, the method fails to follow PT, which is the main point of the manuscript.

In addition, there are strong peaks at 400.5-401 eV in the GS as well as at all times after excitation, but these are not discussed.

We thank the reviewer for highlighting this important point regarding the apparent discrepancy between the OTA, TDDFT and XAS data.

From the new OTA measurements performed under controlled pH = 1 conditions, the protonation time constant is approximately 460 ps. As a result, at 300 ps the protonated population has reached only about 60% of its final value, where the final protonated population is approximately 73% at 1 ns. Therefore, the XAS measurements at 300 ps probe an incomplete population of the protonated species, which explains why the spectral changes do not reach the full amplitude predicted by the TDDFT calculations.

Regarding the spectral features at 400.5–401 eV, we have added the following sentence to the main manuscript, at the beginning of the XAS section:

Transitions at higher energy correspond to excitations into higher-lying unoccupied molecular orbitals and are not considered further in this work.

2. The authors state on p. 3 that there are no previous time-resolved (TR) optical studies of this complex. This is clearly incorrect. The authors themselves cite data from TR-PL and optical transient absorption studies in ref 35 and 40 and there are many more of exactly this complex, and closely related Ru(II) photobases, e.g. J. Am. Chem. Soc. 129 (2007) 6968 and J. Phys. Chem. A 2022, 126, 27, 4349–4358. Moreover, the processes and time scales reported in the manuscript are already known, meaning that any novelty must lie in the method, not in the conclusions.

We have modified the sentence as followed:

~~*Time-resolved optical studies, which have not been previously reported for this system, are necessary to set the initial conditions for the X-ray mechanistic studies.*~~

Time-resolved emission measurements and single point transient absorption have been reported for this system[34,35,36]. Here, we conduct broadband transient absorption to capture the full spectral evolution and establish the excited-state initial conditions for the X-ray experiments.

3. Photobases have been studied in detail before, including ultrafast studies with TRIR and TR-PL (see e.g. JACS, 2023, 145, 6612) and have shown considerable mechanistic complexity, far beyond what is reported here by TR-XAS/-XSS. These studies have typically used strong photobases (pKa of H+Base* >13) so that H2O or weak acids are the primary proton donors. In the present study, the weak basicity (pKa of H+Base* = 3.5) and acidic pH means that H3O+(aq) must be the primary donor, and the ca. 100 ps time constant for the proposed protonation fits well with the expected diffusion-controlled protonation by H3O+ at pH = 1 (kp = 1.4E11 M-1s-1). The nature of the primary donor is not discussed, nor if this makes an important difference to the previous studies.

{JACS, 2023, 145, 6612} does not exist. It appears most likely that the reviewer is referring to {JACS, 2023, 145, 6682}. We recognize that well-established techniques such OTA, TR-PL and TRIR can be used to distinguish different mechanisms in systems that involve products/intermediates with distinct spectral signatures and proton-transfer timescales, such as in the cited work.
The point of this work was not to explore mechanistic complexity, as time-resolved X-ray techniques are not sufficiently established to reliably explore more complex PCET

reactions. This goal of this work was thus to explore the additional information that these techniques can provide on simpler, well characterized systems – for example, neither OTA, TR-PL nor TRIR provide the orbital-level description of electronic redistribution that we report in this work. Additionally, TR-XSS is a direct structural probe sensitive to changes in solute-solvent interactions, and to our knowledge this is the first attempt to directly probe solvent reorganization upon protonation of a solvated molecule. We anticipate that this extra level of understanding will prove useful in the study of more complex mechanisms, but needs to first be established on simpler systems.

Furthermore, both experiments were performed at low repetition rate available at LCLS at the time. Upgraded and upcoming capabilities will significantly enhance data quality and open new opportunities for probing photochemical reactions.

We have clarified these points in the Conclusions to further contextualize our work:

Together, these measurements provide the first integrated view of electronic and solvent reorganization during a model PCET reaction. We show that time-resolved X-ray methods provide additional insights into a well-characterized PCET system: TR-XAS yields an orbital-level description of electronic redistribution, while TR-XSS directly probes solute–solvent structural changes. The X-ray experiments were performed at 120 Hz, and recent upgrades to kHz repetition rates will significantly improve signal-to-noise and enable more advanced experiments, including resonant inelastic soft X-ray scattering and broader applicability of XSS beyond heavy-atom systems. Th~~e~~is increasing ability to spectrally distinguish the charge-transfer and protonation steps with site-specificity and direct structural sensitivity offers a compelling proof of concept for addressing long-standing mechanistic questions […]. Our multimodal framework establishes ~~a path to disentangle PCET mechanisms in more complex systems and to~~ a path for future studies of more complex PCET mechanisms as X-ray capabilities continue to advance, and to inform the rational design of PCET in catalysis, artificial photosynthesis, and biological energy conversion.

4. The authors motivate the study by the challenges to distinguish different PCET pathways, referring to the degree of concertedness of PT and ET. The problem is typically not the lack of unique signal of the proposed intermediate, however, but that the intermediate often does not accumulate to a detectable degree in an incoherent ensemble measurement. Then TR-XAS is not better than TR in any other spectral regime. It is also difficult to prove the non-existence of an intermediate by direct methods; as far as I am aware, it can only be inferred from comparisons. Moreover, in the current study, the authors choose a photo-base as system, where the protonation cannot be concerted with the excitation, because of the large difference in time scales, so the distinction of mechanisms is not relevant for the present study. Therefore, this study does not convince this reader that progress is made on this specific point either.

This is an excellent point, although lack of a unique signal can also play an important role. In the case of synchronous versus asynchronous concerted PCET, there is no distinct intermediate involved. In such cases, techniques that are sensitive to both electron and proton motion, and have sufficient temporal resolution, may distinguish disparate rates of electron- and proton-transfer in a concerted mechanism. Although we agree that time-resolved X-ray techniques are not sufficiently mature to achieve this ambitious goal, this work opens a pathway theretoward, and concurrent technical advancements further enhance the utility of these techniques.
In the case of sequential mechanisms where an intermediate does not sufficiently accumulate due to the rate of the second step being similar to or greater than that of the initial step, we fully agree with the reviewer's statement that no time-resolved technique will be able to capture this intermediate.

As per our above response, the choice of a (well-studied) photobase as the first PCET system to explore using time-resolved X-ray techniques is to establish the extra information that can be gleaned from these techniques, not obtainable through more established time-resolved techniques, that will allow us to dissect and understand more complex mechanisms where electronic redistribution, protonation, and solvent response are strongly coupled or potentially concerted.

In the Introduction we have modified as following:

~~As a result, there remains active debate over whether electron and proton transfer proceed concertedly or sequentially, and whether these processes are synchronous or asynchronous in many systems.~~

As a result, key mechanistic aspects of PCET remain actively debated, including the interplay between electronic and nuclear degrees of freedom and the timing and coupling of electron and proton motions.

In the Conclusions we have modified as following:

This increasing ability to spectrally distinguish the charge-transfer and protonation steps with site-specificity and direct structural sensitivity offers a compelling proof of concept for addressing long-standing mechanistic questions~~, including whether PCET proceeds sequentially or concertedly, and whether proton and electron motions are synchronous or asynchronous~~. including synchronicity of electron and proton motion in concerted PCET, as well as the specific role of solvent in gating such processes.

Review report for NCOMMS-26-004071-T

The article titled "*Electronic and Solvent Reorganization in Proton-Coupled Electron Transfer Captured by Ultrafast X-rays*" by Kahraman, A., Larsen, C. and Biasin, E *et al*. utilizes transient absorption, time-resolved N K-edge and time-resolved X-ray solution scattering methods to investigate proton transfer step in the triplet metal-to-ligand charge transfer excited state of a Ru(II) chromophore, $[Ru(bpy)_2(bpz)]^{2+}$. The results presented in herein establishes proton transfer timescales from ^{3}MLCT excited state to be ~100 ps – suggesting diffusion-based protonation step and I am very intrigued by the TR-XSS results and simulations that revealed clear solvent reorganization during the proton transfer step.

This manuscript is well written, and the results are mostly in support to the authors conclusions. I believe this will be an important contribution in this field; however, several clarifications need to be addressed before I can recommend this article for publication at *Nature Communications*.

My specific comments as presented below and are not prioritized in terms of significance.

1. Please add characterization data (^{1}H NMR, ^{13}C NMR etc) of the complex even though it was prepared using a reported procedure.

2. Throughout the manuscript the authors highlight importance of these results for mechanistic understanding of PCET process. From page 2, line 37: "*Our choice of a system that undergoes sequential PCET enables clear separation of the timescales of electron and proton transfer,*" the paper clearly addresses proton transfer step, however the timescale of electron transfer is not obvious and not discussed with clarity, and therefor creates confusion on the PCET introduction. While with proton this is extremely challenging, I wonder if the authors have tried or have any future plan to perform similar experiments with a coupled donor-acceptor type system where electron transfer and proton transfer steps can be distinguished with more clarity.

3. On page 3, line 59, the authors claim that time-resolved studies have not been carried out on this Ru(II) system, however reference 40 performed time-resolved emission measurements on this system. While I am aware of the distinction of this work as compared to the ref 40, for broader audience, a necessary adjustment to the sentence is requested.

4. Figure 1(A): The authors assign the 2-3 ps time constant for singlet-to-triplet conversion, which do not align well with previous reports on Ru(II) polypyridyl complexes (McCusker, J. et al. Science 1997, Okada, T. et al. JACS 2002 and many others). In fact, the authors commented on this time component in the main text and assigned it to a vibrational cooling process, so it just may be a printing error and should be fixed.

5. The authors assign the ~97 ps component as a new species based on the change in lifetimes and ESA bands. To me, the kinetic data is more evident for such assignment and need to be discussed in the main paper. At pH 1, the kinetics monitored at 376 nm, shows a change in sign with a similar time constant (~100 ps) [*also in the Kinetic data probed at 380 nm*

in Figure S4]. A change in sign in a TA kinetics can only be explained by change in electronic state or evolution of a different species with different molar extinction coefficient.

6. While it is unrealistic to ask to perform XAS experiments in D_2O/DCl, have the authors performed TA experiments to further support diffusion-based proton transfer assignment to the 100 ps time constant?

7. In Figure S3, it seems like the relative intensity of ESA and GSB bands for 400 nm and 510 nm photoexcitation changes. This is further evident from the amplitudes of the kinetic plots. Can the authors comment of this observation? A zoomed in plot presenting the dynamics in the 0 – 20 ps timescales would be a good addition to the paper.

8. One would expect to observe blue shift in the XAS after protonation as compared to the MLCT state – as also predicted by the computation, however from the XAS data this is not obvious. Can the authors discuss why the blue shift is not seen experimentally?

9. ChemDraw perspective structures presented in Figure 1A and 3D are not consistent and should be consistent throughout the manuscript.

10. Could the authors elaborate the choice of this chromophore for this particular study (beyond the one sentence mentioned in the last paragraph of the introduction of the paper).
